# Safety, efficacy, and dose response of the maturation inhibitor GSK3532795 (formerly known as BMS-955176) plus tenofovir/emtricitabine once daily in treatment-naive HIV-1-infected adults: Week 24 primary analysis from a randomized Phase IIb trial

**DOI:** 10.1371/journal.pone.0205368

**Published:** 2018-10-23

**Authors:** Javier Morales-Ramirez, Johannes R. Bogner, Jean-Michel Molina, Johan Lombaard, Ira B. Dicker, David A. Stock, Michelle DeGrosky, Margaret Gartland, Teodora Pene Dumitrescu, Sherene Min, Cyril Llamoso, Samit R. Joshi, Max Lataillade

**Affiliations:** 1 Clinical Research Puerto Rico Inc., San Juan, Puerto Rico; 2 Med IV, Hospital of the University of Munich, Munich, Germany; 3 Hôpital St Louis, Paris, France; 4 Josha Research, Bloemfontein, South Africa; 5 ViiV Healthcare, Branford, Connecticut, United States of America; 6 Bristol-Myers Squibb, Wallingford, Connecticut, United States of America; 7 ViiV Healthcare, Research Triangle Park, North Carolina, United States of America; 8 GlaxoSmithKline, Upper Merion, Pennsylvania, United States of America; Azienda Ospedaliera Universitaria di Perugia, ITALY

## Abstract

GSK3532795 (formerly known as BMS-955176) is a second-generation maturation inhibitor targeting a specific Gag cleavage site between capsid p24 and spacer peptide 1 of HIV-1. Study 205891 (previously AI468038) investigated the efficacy, safety, and dose response of GSK3532795 in treatment-naive, HIV-1-infected participants. Study 205891 (NCT02415595) was a Phase IIb, randomized, active-controlled, double-blind, international trial. Participants were randomized 1:1:1:1 to one of three GSK3532795 arms at doses 60 mg, 120 mg or 180 mg once daily (QD), or to efavirenz (EFV) at 600 mg QD, each in combination with tenofovir disoproxil fumarate and emtricitabine (TDF/FTC) (300/200 mg QD). Primary endpoint was proportion of participants with plasma HIV-1 RNA <40 copies/mL at Week 24. Between May 2015 and May 2016, 206 participants received treatment. At Week 24, 76–83% participants receiving GSK3532795 and 77% receiving EFV achieved HIV-1 RNA <40 copies/mL. Fifteen participants receiving GSK3532795 and one receiving EFV met resistance testing criteria; 10/15 receiving GSK3532795 had emergent substitutions at reverse transcriptase positions M184, and one at position K65, while the participant receiving EFV did not have any nucleoside reverse transcriptase inhibitor (NRTI)/non-NRTI mutations. EFV, relative to GSK3532795, had more serious adverse events (9% versus 5%) and adverse events leading to discontinuation (17% versus 5%). However, 3–4-fold higher rates of gastrointestinal adverse events were observed with GSK3532795 relative to EFV. GSK3532795 combined with TDF/FTC is efficacious with 24 weeks of therapy. However, GSK3532795 showed a higher rate of gastrointestinal intolerability and treatment-emergent resistance to the NRTI backbone relative to EFV.

**Trial registration**: ClinicalTrials.gov NCT02415595.

## Introduction

Despite significant advances in the treatment of HIV-1 infection and the availability of more than 35 approved therapies [1,2], there continues to be unmet medical needs in the event of drug resistance (both acquired transmitted and emergent), long-term intolerability, and drug–drug interactions. Comorbidities in patients using current treatment options also pose a significant problem and complicate treatment decisions. New drugs with novel mechanisms of action could potentially fulfill these unmet needs and be used as part of a preferred combination antiretroviral therapy regimen.

The HIV-1 maturation process is essential for the production of infectious virions. This final step in the HIV-1 life cycle involves viral protease cleavage between capsid p24 and spacer peptide 1 within HIV-1 Gag, leading to rearrangement of the virion, condensation of the capsid core and release of infectious virus from the host cell [3,4]. Disruption of this maturation step results in the production of non-infectious HIV-1 particles, and exploitation of this feature therefore offers a crucial target for the development of newer therapeutic agents [5,6]. A prior maturation inhibitor (MI), bevirimat [7,8], ceased clinical development due to lack of activity towards naturally occurring Gag polymorphisms present in approximately 50% of HIV-1-infected participants. These polymorphisms occurred near the capsid/spacer peptide 1 cleavage site (Gag amino acids 362, 369, 370, and 371) [9–13].

The second-generation MI, GSK3532795, provided a potent antiviral response in a Phase IIa dose-ranging clinical trial, regardless of the presence of baseline Gag polymorphisms, a notable difference relative to bevirimat [14]. Based on the findings from this proof-of-concept study, 205891 was conducted to investigate the efficacy, safety, and dose response of GSK3532795. Here, we report the primary Week 24 findings from the Phase IIb study performed in HIV-1-infected, treatment-naive participants.

## Patients and methods

The protocol for this trial (S1 Protocol) is available as supporting information.

### Study outcomes

The primary endpoint was determination of the proportion of participants with plasma HIV-1 RNA <40 copies/mL at Week 24 after treatment with GSK3532795 or efavirenz (EFV). Secondary endpoints included mean changes from baseline in CD4+ T-cell count and mean changes in the percentage of CD4+ T-cell count over time. Secondary safety endpoints included frequency of adverse events (AEs), serious AEs (SAEs), and AEs leading to discontinuation through Week 24. Further secondary endpoints included emergence of drug resistance. Exploratory endpoints included assessment of the impact of Gag polymorphisms on the efficacy of GSK3532795. Prior data demonstrate that viruses harboring single polymorphic variations at the individual Gag positions 362, 369, and 370 have similar susceptibility to GSK3532795. In addition, though Gag A364 is essentially invariant across genotypes, it was included in the primary endpoint analysis since emergent resistance to GSK3532795 (both in vitro and in Phase IIa studies) mapped, in part, to the A364V change [15]. Thus, our analysis at the Week 24 primary endpoint was limited to these four positions. Pharmacokinetic (PK) analyses were performed to evaluate treatment exposure and assess the dose proportionality of GSK3532795 when co-administered with tenofovir disoproxil fumarate (TDF)/emtricitabine (FTC).

### Patients

Eligible participants were a minimum of 18 years of age, antiretroviral treatment-naive (defined as no current or previous exposure to an antiretroviral drug for more than 1 week), with plasma HIV-1 RNA levels of ≥1000 copies/mL and a CD4+ T-cell count >200 cells/μL at screening. Exclusion criteria included, but were not limited to, resistance or partial resistance to any approved study drug, presence of specific resistance mutations to EFV, TDF, FTC, or protease inhibitors (PIs) as shown by genotypic and/or phenotypic drug resistance testing, and evidence of chronic hepatitis B or hepatitis C infection; susceptibility to GSK3532795 was not prospectively measured nor considered a criterion for inclusion/exclusion. All participants provided written informed consent in agreement with the principles of the Declaration of Helsinki.

### Study design

205891 was a Phase IIb, randomized, active-controlled, double-blind trial performed at 58 sites in 12 countries across South America, North America, South Africa, and Europe. Participants were randomized 1:1:1:1 to one of four treatment arms; GSK3532795 at doses of 60 mg, 120 mg or 180 mg once daily (QD), and one reference arm with EFV at a dose of 600 mg QD, each given in combination with TDF/FTC 300/200 mg QD. EFV was selected as the reference arm, owing to its use in treatment-naive adults receiving initial therapy at the time of planning this study. TDF/FTC was used as a background as it has an established safety, efficacy, and tolerability profile.

Participants meeting the study criteria were randomized via the interactive voice response system/interactive web response system (IVRS/IWRS) and stratified by HIV-1 subtype (CRF01_AE versus other). Sponsors were blinded to GSK3532795 or EFV assignment and dose until the last participant had reached Week 24. Investigators and participants were blinded through at least Week 24. Masking was achieved by using identical-looking placebo tablets to the study drug. TDF/FTC was open label.

This study was conducted in compliance with the International Council for Harmonisation of Technical Requirements for Registration of Pharmaceuticals for Human Use (ICH) Good Clinical Practice (GCP). This study complies with US 21 Code of Federal Regulations (CFR) 312.120. The study protocol and its amendments, participant informed consent forms, and other information as required, were reviewed and approved by a national, regional, or investigational center ethics committee (EC) or institutional review board (IRB) in accordance with the above regulations. The IRBs for each centre by country were: Argentina (Comité de Bioética de la Fundación Huésped; Comité Independiente de Ética Prof Zieher, para Ensayos en Farmacología Clínica; Comité de Etica Centralizado de Asistencia e Investigatión Clínica Integral–CIAP; Comité Institucional de Ética de la Investigación en Salud Oulton Romagosa; Comité de Ética de Protocolos de Investigación); Canada (Ottawa Health Science Network Research Ethics Board (OHSN-REB); University of Manitoba—Bannatyne Campus, Biomedical Research Ethics Board; Quebec Institutional Review Board Services; Centre for Applied Ethics McGill University Health Centre (MUHC)); Chile (Comité de Etica Cientifico del Servicio de Salud Metropolitano De Oriente; Comité Ético Científico, Servicio de Salud Metropolitano Sur; Comité de Evaluación Ético Científico del Servicio de salud metropolitano sur oriente); France (Cpp He De France 8 Ambroise Pare); Germany (Ethik-Kommission der Medizinischen Hochschule Hannover); Italy (Segreteria del Comitate Etico della Provincia Monza Brianza; Comitato Etico dell’ IRCCS Ospedale San Raffaele di milano; Comitate Etico della Provincia di Bergamo; Comitato Etico Milano Area A presso Azienda Ospedale Luigi Sacco; Mexico (Comité de Ética en Investigacion de Promotora Médica Aguascalientes S.A de C.V; Comite de Etica en lnvestigacion de Clinica Bajio Clinba SC; Comité de Ética en Investigación del Instituto Nacional de Enfermedades Respiratorias Ismael Cosío V; Comitè de Ética en lnvestigacion del lnstituto Nacional de Ciencias Mèdicas y Nutrición); Poland (Komisja Bioetyczna przy Dolnoslaskiej Izbie Lekarskiej); Puerto Rico (Western Institutional Review Board); South Africa (Wits Health Consortium Ethics Committee); Spain (Secretarfa Tecnica Comité Ético de investigación Clínica, Hospital Universitario Germans Trias I Pujol); United Kingdom (London-Dulwich Research Ethics Committee); United States (Western Institutional Review Board; Acquired Immunodeficiency Syndrome Research Consortium Of Atlanta Inc Institutional Review Board). This trial is registered with ClinicalTrials.gov (NCT02415595) and the European Clinical Trials Database (EudraCT 2013-005487-26).

### Procedures

Participants attended study visits at screening, on Day 1, and once every 4 weeks from Week 4 to Week 24 (telephonic visits were conducted at Week 20). Participants were expected to continue treatment, then, through Week 96, with visit intervals every 8–12 weeks. A post-dose safety follow-up visit was also conducted 12 weeks after treatment discontinuation. Plasma HIV-1 RNA levels were quantified by the Abbott RealTime HIV-1 assay.

Drug resistance testing was conducted at screening, emergence of protocol-defined virologic failure (PDVF), or in the event of a confirmed or consecutive plasma HIV-1 RNA measurement of ≥400 copies/mL at any time during the study. PDVF was defined as either: (1) confirmed HIV-1 RNA ≥40 copies/mL at any time after prior confirmed suppression to <40 copies/mL; or (2) a confirmed >1 log_10_ copies/mL above the nadir level. Genotypic information required for screening was generated using the GenoSure^®^ MG assay; genotypic information and phenotypic observations during the treatment period were generated using the PhenoSense^®^ combination resistance test.

Physical examinations, measurement of vital signs, and clinical laboratory evaluations were performed at Weeks 4, 8, 12, 16 and 24, and participants were closely monitored for AEs and SAEs throughout. AEs were classified according to MedDRA version 19.0. The severity and relationship to study drug was assessed by the local investigator.

Intensive PK blood samples were collected, over a 24-hour period at Week 2, in a subset of participants (n = 24) for the assessment of GSK3532795, TDF, and FTC. Due to the EFV component of the blinded drug regimens, administration of study drug required both morning and evening dosing,12 hours apart. Intensive PK blood samples were collected at the following time points: 0 hour (pre-dose), 0.5, 1, 1.5, 2, 4, 4.5, 5, 6, 8, 12 (pre-dose), and 24 hours (pre-dose). In addition, all participants provided sparse PK samples (one sample per visit) for the assessment of GSK3532795, TDF, and FTC at visit Weeks 4 to 24. All bioanalytical analyses were conducted by PPD^®^ Laboratories using validated high-performance liquid chromatography/mass spectroscopy assays.

### PK analyses

#### Non-compartmental analysis

Intensive plasma GSK3532795 concentrations were analyzed by non-compartmental analysis using the actual sampling times in WinNonlin version 6.3.0 (Pharsight, St. Louis, Missouri, USA). The following PK parameters were determined: maximum observed plasma concentration (C_max_), time to C_max_ (T_max_), area under the plasma concentration–time curve (AUC) in one dosing interval (AUC_tau_), plasma concentration at the end of a dosing interval (for example, concentration at 24 hours), and pre-dose plasma concentration (C0).

### Statistical analyses

#### Efficacy

This was an estimation study and therefore not powered for statistical significance. The response rate was expected to be approximately 80%, giving a 95% confidence interval range of around 66–90% with 50 participants per treatment arm.

The FDA-defined snapshot algorithm was used for primary endpoint efficacy assessment, using the last plasma HIV-1 RNA value in the pre-defined Week 24 visit window (± 6 weeks) to determine response. The modified intent-to-treat (mITT) population consisted of participants who received at least one dose of GSK3532795 or EFV. The observed population comprised participants who received at least one dose of GSK3532795 or EFV, with plasma HIV-1 RNA data within the Week 24 window.

#### Dose proportionality

To assess the dose proportionality of GSK3532795 when co-administered with TDF/FTC, a mixed-effect model with log-transformed dose as the fixed effect and each participant as the random effect was used to fit a power model [16] in SAS version 9.1.3. The response was log-transformed maximum serum concentration (C_max_) and AUC as calculated by a linear trapezoidal rule from time zero to the end of the dosing interval at steady state (AUC_tau_). A slope of 1 was interpreted as perfect dose proportionality.

#### Population PK and PK/pharmacodynamic modeling

Population PK analyses were conducted via non-linear mixed effects using non-linear mixed-effects modeling (NONMEM) software, version 7, level 2.0 (ICON Development Solutions, Hanover, Maryland, USA).[17] A summary of the population PK modeling methods and exposure response exploratory analyses for efficacy and safety is included in **Supporting information**.

## Results

**Fig 1:**
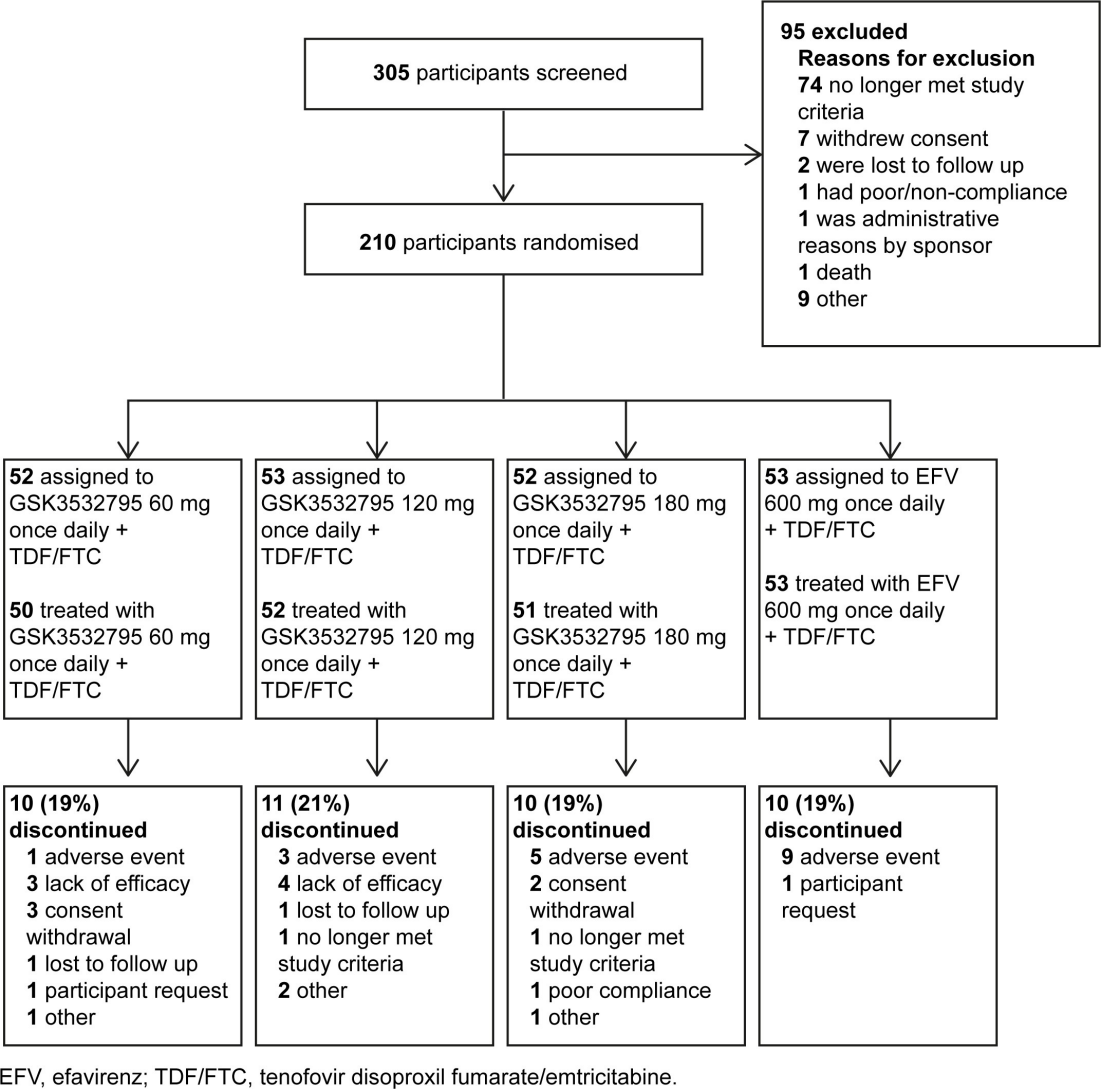
Participant disposition. AE, adverse event; EFV, efavirenz; QD, once daily; TDF/FTC, tenofovir disoproxil fumarate/emtricitabine.

Study recruitment took place between 12 May 2015 and 26 May 2016. A total of 305 participants with treatment-naive HIV-1 infection were enrolled, 210 were randomized and 206 were treated (Fig 1). The most common, non-mutually exclusive reason for screening failures was failure to meet study entry criteria (24% of participants), such as participants having CD4+ T-cell counts <200 cells/μL, HIV-1 RNA <1,000 copies/mL, and/or baseline resistance to study medications. A total of 31/153 (20%) participants receiving GSK3532795 and 10/53 (19%) participants receiving EFV did not complete the study period through Week 24 (Fig 1).

Baseline characteristics were similar across treatment arms and no meaningful clinically significant differences were observed in demographics or disease characteristics (Table 1). Median age was 31 years, 85% were male, 77% White, and 72% participants had HIV-1 subtype B. Almost half of the participants had HIV-1 RNA above 30,000 copies/mL, and 17% of participants had HIV-1 RNA above 100,000 copies/mL; mean baseline plasma HIV-1 RNA was 4.338 (standard deviation [SD] 0.7049) log_10_ copies/mL. One third of participants had a baseline CD4+ T-cell count of 500 cells/μL or above; mean baseline CD4+ T-cell count was 443.5 cells/μL (SD 198.77).

**Table 1:** Baseline participant characteristics.

	GSK3532795 + TDF/FTC			EFV + TDF/FTC	Total
	60 mg (N = 50)	120 mg (N = 52)	180 mg (N = 51)	600 mg (N = 53)	N = 206
**Median age, years (range)**	30 (20–65)	34 (19–57)	31 (22–67)	31 (22–58)	31 (19–67)
Age <50 years, n (%)	49 (98.0)	43 (82.7)	46 (90.2)	49 (92.5)	187 (90.8)
Age ≥50 years, n (%)	1 (2.0)	9 (17.3)	5 (9.8)	4 (7.5)	19 (9.2)
**Male, %**	84.0	84.6	86.3	86.8	176 (85.4)
**Race, %**					
White	78.0	73.1	80.4	75.5	158 (76.7)
**HIV subtype, n (%)**					
B	34 (68.0)	37 (71.2)	35 (68.6)	42 (79.2)	148 (71.8)
C	5 (10.0)	6 (11.5)	6 (11.8)	4 (7.5)	21 (10.2)
CRF01_AE	1 (2.0)	0	0	0	1 (0.5)
Other	11 (22.0)	9 (17.3)	10 (19.7)	7 (13.2)	37 (18.0)
**HIV-1 RNA, mean (SD), log**_**10**_ **copies/mL**	4.336 (0.669)	4.299 (0.730)	4.222 (0.681)	4.489 (0.730)	4.338 (0.705)
**Patients in each category:**					
<30,000 copies/mL, n (%)	28 (56.0)	28 (53.8)	32 (62.7)	29 (54.7)	117 (56.8)
30,000 to <100,000 copies/mL, n (%)	13 (26.0)	17 (32.7)	14 (27.5)	10 (18.9)	54 (26.2)
>100,000 copies/mL, n (%)	9 (18.0)	7 (13.4)	5 (9.8)	14 (26.4)	35 (17.0)
**CD4**^**+**^ **T-cell count, mean (SD), cells/**μ**L**	458.2 (232.2)	460.6 (214.1)	441.7 (182.9)	414.4 (162.7)	443.5 (198.8)
**Patients in each category:**					
<200 cells/μL, n (%)	3 (6.0)	4 (7.7)	3 (5.9)	2 (3.8)	12 (5.8)
200–350 cells/μL, n (%)	18 (36.0)	14 (26.9)	16 (31.4)	17 (32.1)	65 (31.6)
350 to <500/μL, %	12 (24.0)	14 (26.9)	16 (31.4)	22 (41.5)	64 (31.1)
≥500 cells/μL, %	17 (34.0)	20 (38.5)	16 (31.4)	12 (22.6)	65 (31.6)

The GSK3532795 (BMS-955176) phenotypic test has an inherent range maximum of 5.0 μM.

EFV, efavirenz; FTC, emtricitabine; SD, standard deviation; TDF, tenofovir disoproxil fumarate.

For the primary efficacy endpoint, response rates were similar across all three GSK3532795 arms and the EFV arm, when analyzed with both the FDA-defined snapshot algorithm (mITT population) and observed population criteria. Per the mITT analysis, 76–83% of participants receiving GSK3532795 and 77% of those receiving EFV achieved HIV-1 RNA <40 copies/mL (Table 2). In both the GSK3532795 and EFV arms, nine participants each had no virologic data in the visit window. AEs were the most common reason for missing virologic data in the visit window. In the observed analysis, 38/46 (83%), 43/47 (92%), and 42/45 (93%) participants receiving GSK3532795 60 mg, 120 mg and 180 mg, respectively, and 41/44 (93%) participants receiving EFV, achieved HIV-1 RNA <40 copies/mL.

**Table 2:** Proportion of responders with HIV-1 RNA <40 copies/mL (Week 24 snapshot) (mITT).

Outcome, n (%)	GSK3532795 + TDF/FTC			EFV + TDF/FTC
	60 mg (N = 50)	120 mg (N = 52)	180 mg (N = 51)	600 mg (N = 53)
**HIV-1 RNA <40 copies/mL**^**a**^ 95% CI	38 (76.0) 61.8–86.9	43 (82.7) 69.7–91.8	42 (82.4) 69.1–91.6	41 (77.4) 63.8–87.7
**HIV-1 RNA ≥40 copies/mL**	10 (20.0)	7 (13.5)	4 (7.8)	3 (5.7)
HIV-1 RNA ≥40 copies/mL in window^**b**^	8 (16.0)	4 (7.7)	3 (5.9)	3 (5.7)
Discontinued due to lack of efficacy^**c**^	1 (2.0)	2 (3.8)	0	0
Discontinued for other reasons^d^	1 (2.0)	1 (1.9)	1 (2.0)	0
**No virologic data in window**	2 (4.0)	2 (3.8)	5 (9.8)	9 (17.0)
Discontinued due to AE or death^e^	1 (2.0)	1 (1.9)	4 (7.8)	8 (15.1)
Discontinued for other reasons^f^	1 (2.0)	1 (1.9)	1 (2.0)	1 (1.9)

AE, adverse event; CI, confidence interval; EFV, efavirenz; FTC, emtricitabine; mITT, modified intent-to-treat population; TDF, tenofovir disoproxil fumarate.

^a^Response assessed with the snapshot algorithm, which uses the last plasma HIV-1 RNA value in the pre-defined visit window.

^b^Includes participants having HIV-1 RNA ≥40 copies/mL within the Week 24 visit window.

^c^Includes participants who discontinued due to lack of efficacy at any time point from Day 1 through the time window.

^d^Includes participants who discontinued due to any reason except AE, death, and lack of efficacy at any time point from Day 1 through the time window.

^e^Includes participants who discontinued due to AE or death at any time point from Day 1 through the time window.

^f^Includes participants who withdrew consent, lost to follow-up, moved, etc. with last HIV-1 RNA <40 copies/mL.

At Week 24, the mean change in CD4+ T-cell count was similar within the GSK32532795 doses of 60 mg, 120 mg, and 180 mg: 94.3 (SD 175.00), 79.7 (SD 199.46), and 92.5 (SD 144.04) cells/μL, respectively. The greatest mean change from baseline of 134.7 (SD 151.70) cells/μL was observed in the EFV arm.

There was a numerically higher proportion of participants with HIV-1 RNA levels ≥40 copies/mL in the Week 24 (mITT) snapshot window in the GSK3532795 treatment arms relative to the EFV reference arm; 14 participants met the definition of PDVF, with rates of 12% (6/50), 10% (5/52), and 6% (3/51) in the 60 mg, 120 mg, and 180 mg GSK3532795 arms, respectively, and 2% (1/53) with EFV.

Eleven participants who were considered PDVF, and an additional five participants with no virologic response, had both baseline and on-treatment resistance testing performed prior to the Week 24 data analysis (Table 3). Of these 16 participants, 13 were sequenced successfully and had reverse transcriptase genotype at both baseline and on treatment. In the GSK3532795 arms, 10/12 participants had emergent nucleoside/nucleotide reverse transcriptase inhibitor (NRTI) mutations M184V, M184I/V, or M184I with consequent resistance to FTC. NRTI resistance-associated mutations emerged in 3/5, 5/5, and 2/2 participants across the 60 mg, 120 mg, and 180 mg GSK3532795 arms, respectively, who were sequenced successfully (Table 3). One participant in the GSK3532795 180 mg arm developed NRTI mutations M184V and K65K/R, and another participant in the GSK3532795 60 mg arm had emergent M184V and K70K/E. However, neither participant had phenotypic resistance to tenofovir. Non-NRTI (NNRTI) mutations, V106V/I and E138E/G, were detected in one participant each in the GSK3532795 60 mg and 120 mg arms. One participant with M184V NRTI mutation had an emergent, minor protease V77V/I mutation with no emergent phenotypic resistance to any approved PI. The only participant in the EFV arm with on-treatment resistance testing had no emergent NRTI, NNRTI, or PI mutations.

**Table 3:** Treatment-emergent substitutions: Participants meeting drug-resistance testing criteria.

Parameter, n	GSK3532795 + TDF/FTC			EFV + TDF/FTC
	60 mg (N = 5)	120 mg (N = 6)	180 mg (N = 4)	600 mg (N = 1)
**Underwent resistance testing**	5	6	4	1
**PI substitutions**				
**Successfully sequenced**	5	5	2	1
V77 (V/I)	1	0	0	0
**RT/NRTI/NNRTI substitutions**				
**Successfully sequenced**	5	5	2	1
**RT/NRTI substitutions**	3	5	2	0
K65K/R	0	0	1	0
K70K/E	1	0	0	0
M184I	1	1	0	0
M184I/V	0	1	0	0
M184V	2	3	2	0
**NNRTI substitutions**	1	1	0	0
V106VI	1	0	0	0
E138E/G	0	1	0	0
**Gag substitutions (selected positions: 362, 364, 369, 370)**				
**Successfully sequenced**	4	3	1	0
V362I	2	0	0	0
A364V	0	1	0	0
A364A/V + V362V/I	0	1	0	0

Resistance testing was performed for all participants with: (1) confirmed or consecutive HIV-1 RNA ≥400 copies/mL from Week 4 through Week 96; or (2) PDVF (confirmed ≥40 copies/mL if prior suppression to <40 copies/mL or >1 log_10_ copies/mL increase in HIV-1 RNA at any time above nadir level, where nadir is ≥40 copies/mL).

EFV, efavirenz; FTC, emtricitabine; NNRTI, non-nucleoside reverse transcriptase inhibitor; NRTI, nucleoside/nucleotide reverse transcriptase inhibitor; PI, protease inhibitor; RT, reverse transcriptase; TDF, tenofovir disoproxil fumarate.

Emergence of Gag mutations at key positions 362, 364, 369, and 370 were evaluated in the 15 participants receiving GSK3532795 with on-treatment resistance testing. The Gag gene in 8/15 participants was successfully sequenced, both at baseline and on treatment, and of these, 4/8 participants in the GSK3532795 60 mg and 120 mg arms developed treatment-emergent Gag substitutions at positions V362 and/or A364 (Table 3).

The effect of baseline Gag polymorphisms on virologic response (HIV-1 RNA <40 copies/mL) was evaluated through Week 24. Key Gag substitutions at positions 362, 369, and 370 were evaluated; position 364 was excluded, as no polymorphisms at A364 were present at baseline in this study population. The most frequently observed baseline substitution was at position 370. At the higher 120 mg and 180 mg GSK3532795 doses, response rates were similar in participants with substitutions at position 370 (21/25 [84%] and 20/24 [83%], respectively) relative to participants without baseline polymorphisms (12/16 [75%] and 17/19 [89.5%], respectively). In the GSK3532795 60 mg arm, fewer participants achieved HIV-1 RNA <40 copies/mL with a Gag polymorphism at position 370 (11/17 [65%]) relative to those without any Gag polymorphisms (19/24 [79%]). The numbers of participants with baseline substitutions at positions 362 and 369 were too small to draw clinical inference.

Most participants receiving EFV (48/53 [91%]) or GSK3532795 (131/153 [86%]) reported ≥1 AE (Table 4). However, more participants receiving GSK3532795 had gastrointestinal (GI) disorders (99/153 [65%]) relative to EFV (16/53 [30%]), and those receiving EFV reported higher nervous system disorders (dizziness/headache; 25/53 [47%]) relative to GSK3532795 (16/153 [10.5%]). Dizziness was reported in 19/53 (36%) participants in the EFV arms, relative to 2/153 (1%) participants across the GSK3532795 arms (Table 5). A higher frequency of Grade 1–4-related AEs was observed with EFV (60%) relative to the GSK3532795 arms (53%) (Table 5). Grade 1–4-related diarrhea and abdominal pain were observed in 27–51% and 6–16% of participants receiving GSK3532795, respectively, relative to 4% and 0 participants receiving EFV. The proportion of related GI AEs considered Grade 3–4 was 6/153 (4%) in participants receiving GSK3532795 relative to 4/53 (7.5%) in participants receiving EFV.

**Table 4:** Safety summary through Week 24.

Parameter, n	GSK3532795 + TDF/FTC			EFV + TDF/FTC
	60 mg (N = 50)	120 mg (N = 52)	180 mg (N = 51)	600 mg (N = 53)
Participants with ≥1 AE	41 (82%)	45 (87%)	45 (88%)	48 (91%)
Deaths	0	0	0	0
Serious AEs	1 (2%)	2 (4%)	1 (2%)	5 (9%)
Related AEs	22 (44%)	26 (50%)	33 (65%)	32 (60%)
AEs leading to discontinuation	1 (2%)	3 (6%)	4 (8%)	9 (17%)

AE, adverse event; EFV, efavirenz; FTC, emtricitabine; TDF, tenofovir disoproxil fumarate.

**Table 5:** Grade 1–4 treatment-related AEs through Week 24 (treated participants).

	GSK3532795 + TDF/FTC			EFV + TDF/FTC	Total
n (%)	60 mg (N = 50)	120 mg (N = 52)	180 mg (N = 51)	600 mg (N = 53)	N = 206
**Overall Grade 1–4 treatment-related AEs**					
Total participants with an event	22 (44.0)	26 (50.0)	33 (64.7)	32 (60.4)	113 (54.9)
**Grade 1–4 treatment-related AEs by SOC**^a^ **(≥5%)**					
**Gastrointestinal disorders**	21 (42.0)	23 (44.2)	31 (60.8)	7 (13.2)	82 (39.8)
Diarrhea	15 (30.0)	14 (26.9)	26 (51.0)	2 (3.8)	57 (27.7)
Abdominal pain	3 (6.0)	5 (9.6)	8 (15.7)	0	16 (7.8)
Nausea	2 (4.0)	1 (1.9)	5 (9.8)	5 (9.4)	13 (6.3)
Upper abdominal pain	1 (2.0)	2 (3.8)	3 (5.9)	0	6 (2.9)
**Nervous system disorders**	1 (2.0)	2 (3.8)	2 (3.9)	20 (37.7)	25 (12.1)
Dizziness	0	1 (1.9)	1 (2.0)	19 (35.8)	21 (10.2)
**Skin and subcutaneous tissue disorders**	0	2 (3.8)	2 (3.9)	12 (22.6)	16 (7.8)
Rash	0	2 (3.8)	0	3 (5.7)	5 (2.4)
Rash macular	0	0	1 (2.0)	3 (5.7)	4 (1.9)
**Psychiatric disorders**	1 (2.0)	3 (5.8)	7 (13.7)	11 (20.8)	22 (10.7)
Insomnia	1 (2.0)	0	6 (11.8)	1 (1.9)	8 (3.9)
Abnormal dreams	0	3 (5.8)	1 (2.0)	5 (9.4)	9 (4.4)
**Respiratory, thoracic, and mediastinal disorders**	5 (10.0)	3 (5.8)	3 (5.9)	4 (7.5)	15 (7.3)
**Ear and labyrinth disorders** (Vertigo)	1 (2.0)	0	0	3 (5.7)	4 (1.9)
**Investigations**^**b**^	0	1 (1.9)	1 (2.0)	3 (5.7)	5 (2.4)

AE, adverse event; EFV, efavirenz; FTC, emtricitabine; SOC, system organ class; TDF, tenofovir disoproxil fumarate.

^a^SOC, as defined according to the latest version of the MedDRA at the time of database lock.

^b^ Alanine aminotransferase, apartate aminotransferase, blood creatine phosphokinase, blood creatinine, blood phosphorus increased.

SAEs occurred in 5/53 (9%) participants in the EFV arm relative to 1/50 (2%), 2/52 (4%), and 1/51 (2%) participants in the GSK3532795 60 mg, 120 mg, and 180 mg arms, respectively (Table 4). Two SAEs were considered related to study drug: Grade 3 abdominal pain was reported with GSK3532795 120 mg (no action taken with study therapy) and increased hepatic enzyme was reported with EFV (led to discontinuation of therapy). No SAEs led to death during the treatment phase of the study. A higher proportion of discontinuations due to AEs was observed in the EFV arm (9/53 [17%] participants) relative to the GSK3532795 arms (8/153 [5%] participants) (Table 4). Participants who discontinued in the GSK3532795 arms due to AEs increased with increasing dose from 1/50 (2%) in the 60 mg arm, 3/52 (6%) in the 120 mg arm, and 4/51 (8%) in the 180 mg arm. Discontinuations with EFV were mostly due to skin and subcutaneous disorders, whereas those with GSK3532795 were all attributable to GI disorders.

Both time to onset and duration of diarrhea were assessed. Time to onset of first diarrhea event in the GSK3532795 arms was shorter with increasing dose; mean time to onset was 48.4 days, 19.9 days, and 11.3 days in the 60 mg, 120 mg, and 180 mg arms, respectively, and 73.0 days in the EFV arm. However, across all treatment arms, participants who developed diarrhea (75/206 [36%]) mostly did so during the first week (Days 1–7) (51/75 [68%]). Mean duration of first event was longest in the GSK3532795 120 mg arm (104.2 days) and shortest in the EFV arm (35.8 days) (Table A in S1 Table).

Grade 3 laboratory abnormalities in triglycerides and creatinine clearance (CrCl) was reported in one participant each in the GSK3532795 180 mg arm, and low potassium in one participant in the GSK3532795 60 mg arm. Grade 3 liver chemistry abnormalities were observed in three participants in the GSK3532795 arms: two experienced Grade 3 aspartate aminotransferase elevations that returned to normal at Week 32, and one experienced Grade 3 alkaline phosphatase that returned to normal after 13 weeks. These were all regarded as isolated events. Two participants experienced hepatic events with elevations of several liver chemistry parameters. One participant experienced Grade 3 aspartate aminotransferase elevations, and lower grade alanine aminotransferase and alkaline phosphatase elevations. The participant was ongoing in the study and outcome and treatment arm were unknown at the Week 24 database lock. The other participant experienced several Grade 4 liver chemistry elevations that did not quickly resolve and was discontinued with the treatment arm unblinded. The participant had been receiving EFV and all events were attributed to EFV.

Mean total cholesterol decreased in the GSK3532795 120 mg arm and increased in the remaining treatment arms from baseline to Week 24, with the highest increase in the EFV reference arm of 17.6 mg/dL. Triglyceride levels decreased in all GSK3532795 arms (mean decrease 5.0–12.9 mg/dL), whereas an increase was observed in the EFV arm (mean increase 41.1 mg/dL) from baseline to Week 24.

Following multiple doses of GSK3532795 60 mg, 120 mg, or 180 mg co-administered in the presence of food with TDF/FTC, median steady-state plasma concentrations (assessed on treatment at Week 24) reached peak concentrations at 4–5 hours post-dose (Table A in S2 Table). A less than dose-proportional increase in systemic exposure over the GSK3532795 dose range of 60–180 mg was noted (Table A in S3 Table).

GSK3532795 disposition was described by a one-compartment model with first order absorption (S1 Text). The model structural parameter estimates are presented in Table A in S4 Table. Of the 151 participants that contributed PK data, ~77% were White, ~15% were Black or African American, <1% were Asian, and <7% were categorized as “other”. Of these, a statistically significant reduction in clearance was seen only in the Black/African-American participants when compared with White participants. Participants with Grade 1 diarrhea had similar clearance relative to participants with no diarrhea, whereas participants with Grade ≥2 diarrhea had increased clearance of 18% (1–40%). To explore the exposure-response relationships, efficacy endpoints (proportion of participants with viral load <40 copies/mL at 24 weeks) were plotted versus GSK3532795 exposure (AUC) (Figure A in S1 Fig). Similar analysis was conducted for GI AE endpoints Grade 1–4 (Figure B and Figure C in S1 Fig). Overall, the AE and efficacy endpoints did not show strong trends with increasing GSK3532795 exposure.

## Discussion

The analysis of this Phase IIb data is the first to show that combination treatment with an MI with other antiretrovirals can suppress viral replication over time. Specifically, for the primary efficacy endpoint, the proportions of responders in the GSK3532795 120 mg and 180 mg arms were similar, relative to the EFV reference arm (based on the standard snapshot algorithm, and observed analysis). The 120 mg and 180 mg doses of GSK3532795 had a greater proportion of responders at Week 24 than the 60 mg dose.

SAEs, AEs leading to study/study drug discontinuation, and Grade 3–4 AEs were all reported more frequently in the EFV reference arm relative to GSK3532795. The incidence of these safety events was similar, relative to the three GSK3532795 treatment arms. The overall incidence of nervous system disorders and psychiatric disorders was highest in the EFV reference arm, with the incidence of dizziness almost 10-fold higher with EFV relative to GSK3532795.

However, analyses of the Week 24 data revealed GI tolerability concerns associated with GSK3532795. The incidence of GI AEs (regardless of grade or relationship) in the GSK3532795 arms was 3–4-fold higher relative to the EFV arm, and the incidence of GI events with GSK3532795 appeared to increase in a dose-related manner. Although GI AEs were the most frequently reported AEs leading to discontinuation of GSK3532795, an exploratory analysis of exposure in participants with Grade 1–4 GI AEs showed no correlation to plasma GSK3532795 exposure parameters. This indicates that the GI intolerability observed in GSK3532795-treated participants is more likely to be related to drug dose rather than drug exposure. The time to onset of diarrhea showed a dose-response relationship, with shorter time to onset associated with higher doses.

There was a higher percentage of participants meeting PDVF in the GSK3532795 arms relative to EFV, with an inverse relationship between the development of PDVF and GSK3532795 dose. Virologic failure was associated with development of NRTI RAMs (most commonly, M184V, M184I/V, or M184I) and treatment-emergent substitutions in Gag at positions 362 and 364. Notably, there was no treatment-emergent resistance in the EFV arm. The rate of treatment-emergent NRTI resistance observed across the GSK3532795 treatment arms was higher than current recommended cART regimens for HIV-1-infected, treatment-naive adults.

At the GSK3532795 120 mg and 180 mg doses, efficacy at Week 24 was similar in participants with or without a baseline Gag substitution at position 370. Furthermore, these response rates were similar to EFV and generally corroborate data from the prior proof-of-concept study [14]. However, participants receiving the GSK3532795 60 mg dose displayed reduced efficacy at Week 24 in the presence of Gag polymorphic variation at position 370. The numbers of participants with baseline Gag substitutions at positions 362 and 369 were too small to draw any meaningful clinical inference.

Systemic exposure to GSK3532795 co-administered in the presence of food with TDF/FTC increased with dose in a less than dose-proportional manner. GSK3532795 disposition was adequately described by a one-compartment model with first order absorption. Clearance was reduced by 23% in African-American participants. In addition, Grade 2 and higher diarrhea resulted in an 18% increase in clearance. Efficacy endpoints demonstrated little correlation to GSK3532795 exposure. It is likely that lower doses would need to be studied to elucidate clear exposure-response relationships.

Our study had several strengths: given the well-established clinical profile of the background therapy of TDF/FTC, particularly in HIV-1-infected treatment-naive participants, combination with GSK3532795 allowed us to have a clearer understanding of dose-response relationship, safety/tolerability, and resistance. There were several limitations to our study. First, we used EFV as a reference arm as this trial was designed when combination therapy with EFV was listed as a preferred therapy for treatment-naive adults, per treatment guidelines. Second, women represented only ~15% of the study population. Third, our study population had minimal representation of HIV-1 subtype AEs given the geographic footprint of our multinational trial. Finally, phenotypic data for GSK3532795 was not available at the time of the analysis of the primary endpoint; thus, there are insufficient data from which to elucidate a relationship between baseline substitutions and treatment-emergent changes in Gag and any changes in susceptibility for GSK3532795. Further research will be needed in this regard.

In summary, despite significant Week 24 efficacy rates (as seen within the Week 24 snapshot) that are similar to EFV, the clinical development program of GSK3532795 was terminated early due to higher rates of GI AEs and frequency of treatment-emergent NRTI resistance. Although GSK3532795 is not progressing to Phase III studies, the antiviral response rates and immunologic reconstitution for GSK3532795 (in combination with other antiretrovirals) observed, along with its novel mechanism of action, are promising and support the continued development of the MI class of anti-HIV-1 agents.

## Supporting information

S1 CONSORT Checklist (DOC)

S1 Protocol (PDF)

S1 Table **(A) Onset and duration of diarrhoea**. EFV, efavirenz; FTC, emtricitabine; Max, maximum; Min, minimum; Q1, the first quartile; Q3, the third quartile; SD, standard deviation; TDF, tenofovir disoproxil fumarate.
(DOCX)

S2 Table **(A) Treatment exposure**. AUC, area under curve; CV, coefficient of variation; FTC, emtricitabine; TDF, tenofovir disoproxil fumarate.
(DOCX)

S3 Table **(A) Dose proportionality assessment for GSK3532795 Cmax and AUC(TAU) in the evaluable population**. AUC, area under curve calculated by linear trapezoidal rule from time zero to the end of the dosing interval at steady state; CI, confidence interval; Cmax, maximum serum concentration.
(DOCX)

S4 Table **(A) Parameter estimates from the final GSK3532795 population PK model. CIs are derived from the bootstrap**. CI, confidence interval; CL, clearance; CV, coefficient of variation; RSE, residual standard error; PK, pharmacokinetic; SD, standard deviation.
(DOCX)

S1 Fig **(A) Fraction of participants with viral load <40 copies/mL at Week 24 versus steady state AUC: mITT population. (B) Exposure-response relationship for Grade 1 GI AEs. (C) Exposure-response relationship for Grade 2 GI AEs**. Mean response is plotted at the mean of the exposure quantile. The observed values are also plotted against individual exposures, represented by black filled circles. The dashed blue line represents a loess smooth of the individual data, with a shaded blue region representing the 95% CI of the loess smooth. Range of exposures within a quartile group is shown at the bottom of the figure as a horizontal line with vertical ticks representing minimum and maximum values. The values along the horizontal line indicate the number of individuals within each exposure quartile/covariate group. The horizontal bars at the top of the figure show the overall PK variability in exposure following 60 mg, 120 mg, or 180mg GSK3532795.
(DOCX)

S1 Text Additional methods. (DOCX)
