# Supplementary material for: Safety, efficacy, and dose response of the maturation inhibitor GSK3532795 (formerly known as BMS-955176) plus tenofovir/emtricitabine once daily in treatment-naive HIV-1-infected adults: Week 24 primary analysis from a randomized Phase IIb trial
Source: PLoS One. 2018 Oct 23;13(10):e0205368. doi: 10.1371/journal.pone.0205368 (PMC6198970; doi:10.1371/journal.pone.0205368)
Supplement: S1 Protocol — (PDF) [file pone.0205368.s002.pdf]

Protocol Number: AI468038
IND Number: 118,936
Ex-US Non-IND
EUDRACT Number 2013-005487-26
Date: 28-Jan-2015
Revised Date: 22-May-2015

Clinical Protocol AI468038

A Phase 2b Randomized, Active-Controlled, Double-Blind Trial to Investigate Safety, Efficacy and Dose-response of BMS-955176, Given on a Backbone of Tenofovir/Emtricitabine, in Treatment-Naive HIV-1 Infected Adults

Revised Protocol Number: 02
Incorporates Amendment 03

Study Director

Samit Joshi, DO, MPH
5 Research Parkway
Office 201D
Wallingford, CT 06492 USA
Telephone (office): 203-677-3613
Fax: 203-677-6002

Central Medical Monitor

Carey Hwang, M.D., Ph.D.
Route 206 & Province Line Road
Office E1.256
Lawrenceville, NJ 08543 USA
Telephone (office): 609-252-3597
Fax: 609-252-7035

24-hr Emergency Telephone Number
USA: 1-866-470-2267
International: +1-248-844-7390

Bristol-Myers Squibb Research and Development
Virology Clinical Research and Development

Avenue de Finlande 4
B-1420 Braine-l’Alleud, Belgium

Route 206 & Province Line Road
Lawrenceville, NJ 08543

5 Research Parkway
Wallingford, CT 06492

Replace all previous version(s) of the protocol with this revised protocol and please provide a copy of this revised protocol to all study personnel under your supervision, and archive the previous versions.

Approved v 3.0 930087119 3.0

DOCUMENT HISTORY

Document	Date of Issue	Summary of Change
Revised Protocol 02	22-May-2015	Incorporates Amendment 03
Amendment 03	22-May-2015	To remove “male condoms with spermicide” as a highly effective method of contraception and require the use of a highly effective method of contraception as specified in the Clinical Trials Facilitation Group Recommendations Related to Contraception and Pregnancy Testing in Clinical Trials. Most contraception methods were more clearly defined, including the removal of any text relative to hormonal methods used by female study subjects who are WOCBP, since they can’t be counted among the methods used at all.<br><br>There may be instances where study participants may choose not to continue in the roll over study, but rather switch to other approved and marketed antiretroviral therapy based on discussion with their health care provider. Therefore, the protocol was modified to incorporate a post-dosing safety follow-up visit 12 weeks after early discontinuation or after Week 96 (end of study). In addition, WOCBP are now required to follow study instructions for methods of contraception for 12 weeks post treatment completion.<br><br>In addition, at Week 24, a Time to Loss of Virologic Response analysis will be conducted as a sensitivity analysis that compliments the snapshot analysis, and a definition of virologic rebound was added.<br><br>A table of the laboratory assessments in detail was added as an appendix, and the appendix of the DAIDS Toxicity Table (updated 2009) was removed to include a link to the more current 2014 version of the table.<br><br>An administrative change was made to clearly indicate that AIDS History will be taking at Screening, as well as other minor administrative changes
Revised Protocol 01	29-Apr-2015	Incorporates Amendment 02
Amendment 02	29-Apr-2015	The criterion that excluded from the study subjects with “3 or more” of the listed thymidine analog mutations (TAMs) was changed to further restrict exclusion to subjects having “any” of the listed TAMs: M41L, D67N, K70R, L210W, T215Y/F, or K219Q/E (the list of TAMs was not modified). This change was made to ensure optimal susceptibility to tenofovir disoproxil fumarate as previous studies demonstrated a decrease in HIV-1 RNA decline in the presence of certain combinations of TAMs1.<br><br>In addition, protocol defined virologic failure was clarified to read HIV-1 RNA confirmed ≥ 40 copies/mL if prior suppression to < 40 copies/mL or confirmed > 1 log10 copies/mL increase in HIV-1 RNA above nadir at any time. This clarification and simplification alleviates confusion from the definition as previously written criteria that had defined PDVF in various ways for different time points.<br><br>It was also clarified that resistance testing should occur for all

Approved v 3.0 930087119 3.0

Document	Date of Issue	Summary of Change
		subjects with HIV-1 RNA viral load > 400 copies/mL between Week 4 and 96. This alleviates any ambiguity and will ensure that clinical sites will send samples for resistance testing.<br><br>The amendment also corrects the collection of Sparse PK samples to indicate that only one visit Weeks 4 -24 (as opposed to all of the visits Weeks 4 - 24 as outlined in the original protocol) needs to be performed in the morning and to have the blood collected as a pre-AM dose sampling.<br><br>In addition, this amendment includes the detail for the BMS-955176 120 mg tablet is film-coated, as well as a modification regarding discontinuation for reason of pregnancy so that all references to it in the protocol are consistent.
Original Protocol	28-Jan-2015	Not applicable

Approved v3.0 930087119 3.0

SYNOPSIS

Clinical Protocol AI468038

Protocol Title: A Phase 2b Randomized, Active-Controlled, Double-Blind Trial to Investigate Safety, Efficacy and Dose-response of BMS-955176, Given on a Backbone of Tenofovir/Emtricitabine, in Treatment-Naive HIV-1 Infected Adults

Investigational Product(s), Dose and Mode of Administration, Duration of Treatment with Investigational Product(s): Subjects in each arm will begin dosing QD with BMS-955176 (60, 120, or 180 mg)/placebo and efavirenz (EFV)/placebo, in combination with open-label tenofovir/emtricitabine (TDF/FTC), with the following drugs/doses, for the duration of the blinded portion of the study.

- Arm 1: BMS-955176 60 mg QD + TDF/FTC 300/200 mg QD, or
- Arm 2: BMS-955176 120 mg QD + TDF/FTC 300/200 mg QD, or
- Arm 3: BMS-955176 180 mg QD + TDF/FTC 300/200 mg QD, or
- Arm 4: EFV 600 mg QD + TDF/FTC 300/200 mg QD

Study Phase: 2b

Research Hypothesis: The Phase 2b study will demonstrate that at least one dose of BMS-955176 will be efficacious, safe, and tolerable for use in HIV-1 infected treatment-naïve adults

Objectives:

Primary Objective

- To evaluate antiviral efficacy of 3 doses (60, 120 and 180 mg) of BMS-955176, and EFV, each when given in combination with TDF/FTC in treatment-naïve subjects by determining the proportion of treatment-naïve subjects with plasma HIV-1 RNA < 40 c/mL at Week 24

Secondary Objectives

- To assess the antiviral efficacy of BMS-955176 and EFV by determining the proportion of subjects with plasma HIV-1 RNA < 40 c/mL at Weeks 48, and 96
- To assess the antiviral efficacy of BMS-955176 and EFV by determining the proportion of subjects with plasma HIV-1 RNA < 200 c/mL at Weeks 24, 48, and 96
- To assess the emergence of HIV drug resistance among samples selected for drug resistance testing (according to criteria outlined in Protocol Section 5.4.1)
- To assess efficacy of BMS-955176 and EFV, by using mean changes from baseline in $\log_{10}$ HIV-1 RNA, CD4+ T-cell counts, and percentage of CD4+ T-cells
- To assess the safety and tolerability of BMS-955176 in treatment-naïve subjects by measuring frequency of SAEs and AEs leading to discontinuation
- To assess disease progression as measured by the occurrence of new AIDS defining events (CDC Class C events)
- To characterize the pharmacokinetics of BMS-955176 when co-administered with TDF and FTC in treatment-naïve HIV-1 infected subjects.

Study Design:

This is a randomized, active-controlled, double-blind clinical trial. Approximately 200 treatment-naïve HIV-1 infected subjects will be randomized 1:1:1:1 to one of four different treatment arms (approximately 50 per arm).

Approved v3.0 930087119 3.0

Randomization will be stratified by HIV-1 Clade (AE versus Other). The number of subjects with HIV-1 Clade AE will be capped at a maximum of approximately 16 total, approximately 4 per treatment arm.

A Continuation Dose of BMS-955176 will be selected: A dose of BMS-955176 will be selected, based on the analysis of Week 24 data, to which subjects in Arms 1-3 will transition for the remainder of the study. After the selection of a Continuation Dose of BMS-955176, subjects in the BMS-955176 arms will be switched to that open-label BMS-955176 dose; subjects in the EFV Arm will switch to open-label EFV. The switch is expected to occur on or after Week 48. The open-label backbone of TDF/FTC will remain unaltered.

All subjects are expected to receive study treatment for 96 weeks.

Approved v 3.0 930087119 3.0

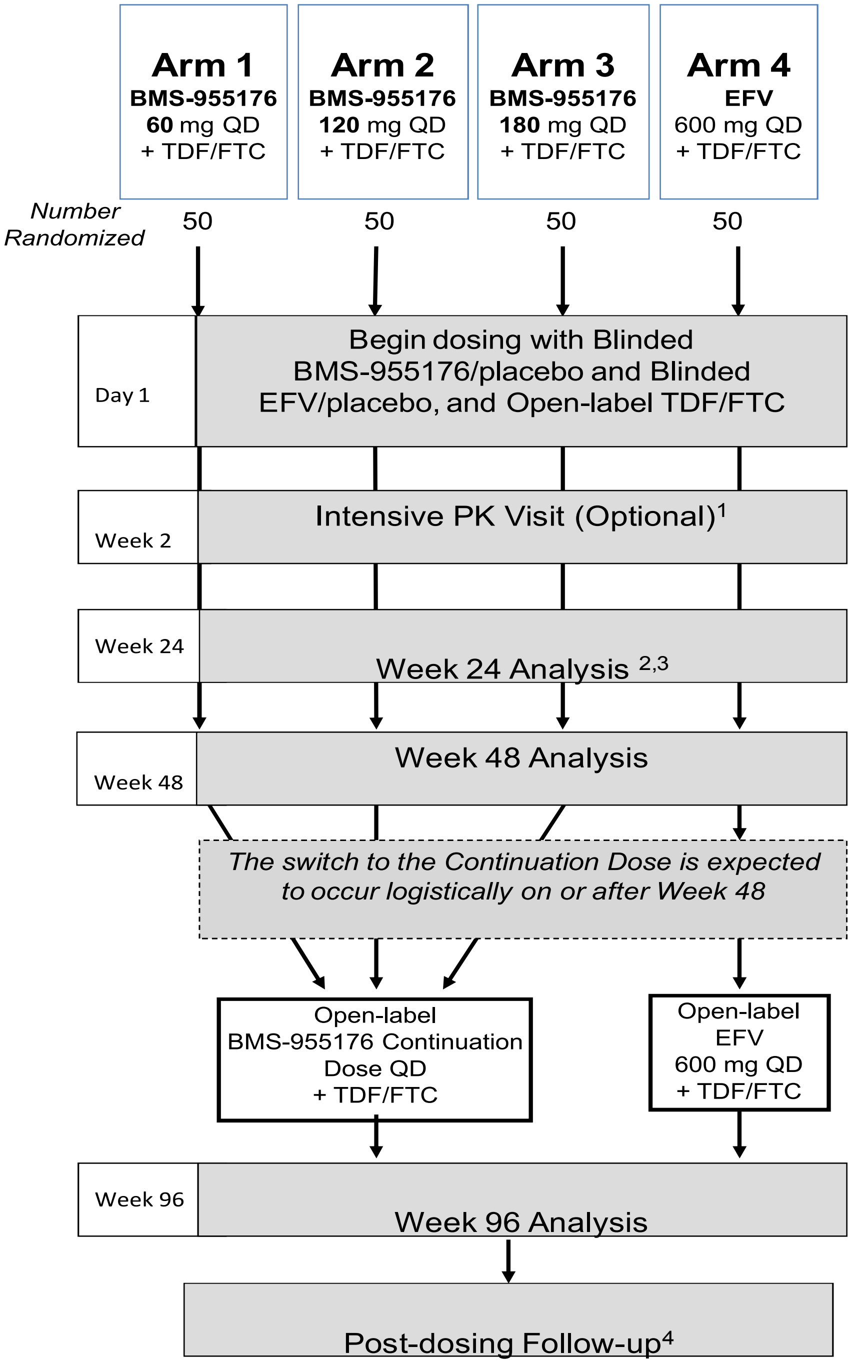

1 *Subjects with Hemoglobin < 11.0 g/dL should be excluded from participation in the optional Week 2 Intensive PK visit.*

2 *Virologic Futility will be evaluated (see Section 8.4.7)*

3 *The selection of the Continuation Dose will be made using the Week 24 data. If selection of the Continuation Dose cannot be made using the Week 24 data, the assessment will be made using Week 48 data.*

4 *Subjects who discontinue the study early, or who reach Week 96 but choose not continue with BMS supplied study drugs via the methods described in Section 3.2, are required to be seen for one visit 12 weeks after end of study treatments.*

Approved v3.0 930087119 3.0

Study Population:

Key Inclusion Criteria:

- Men and non-pregnant women, at least 18 years of age (or minimum age as determined by local regulatory or as legal requirements dictate)
- Antiretroviral treatment-naïve; defined as no current or previous exposure to > 1 week of an antiretroviral drug
- Plasma HIV-1 RNA ≥ 1000 copies/mL
- CD4 T-cell count > 200 cells/mm^3

Key Exclusion Criteria:

- Resistance or partial resistance to any study drug determined by tests at Screening
- Current or historical genotypic and/or phenotypic drug resistance testing showing resistance to EFV: K103N, L100I, K101E/P, V106A/M, Y181C/I/V, Y188L/C/H, G190A/S/E, or M230L
- Current or historical genotypic and/or phenotypic drug resistance testing showing resistance to TDF: K65R or T69ins
- Current or historical genotypic and/or phenotypic drug resistance testing showing resistance to FTC: M184I/V or Q151M
- Current or historical genotypic drug resistance testing showing any of the following non-accessory thymidine analog mutations (TAMs): M41L, D67N, K70R, L210W, T215Y/F, K219Q/E
- Current or historical genotypic drug resistance testing showing primary protease inhibitor resistance mutations: I50L, I84V/A, N88D/S, I47A/V, V82A/F/T/S. These exclusionary mutations are listed only in order to allow subjects who develop on-treatment emergent virologic resistance a subsequent PI-based regimen (after study discontinuation)
- Chronic HBV/HCV (Positive blood screen for HBsAg; Positive blood screen for HCV Ab and HCV RNA)
- ALT or AST > 3 × ULN
- Alkaline Phosphatase > 5 × ULN
- Bilirubin ≥ 1.5 × ULN
- History of decompensated cirrhosis or active decompensated cirrhosis
- Hemoglobin < 8.0 g/dL
- Platelets < 50,000 cells/mm^3

Study Drug: includes both Investigational [Medicinal] Products (IMP/IMP) and Non-investigational [Medicinal] Products (Non-IMP/Non-IMP) as listed:

Study Drug for AI468038		
**Medication**	**Potency**	**IMP/Non-IMP**
BMS-955176	60 mg[a] and 120 mg[a]	IMP
Placebo to match BMS-955176 60 mg[a], and Placebo to match BMS-955176 120 mg[a]	N/A	IMP
Efavirenz (EFV)	600 mg	IMP

Approved v3.0 930087119 3.0

Study Drug for AI468038		
**Medication**	**Potency**	**IMP/Non-IMP**
Placebo to match Efavirenz (EFV) 600 mg	N/A	IMP
TDF/FTC	300 mg/200 mg[b]	Non-IMP

[a] 180 mg of BMS-955176 active/placebo will be constructed with BMS-955176 60 mg/placebo + BMS-955176 120 mg/placebo

[b] Some regions may be supplied with TD/FTC 245/200 mg; Tenofovir disoproxil (TD) 245 mg is equivalent to tenofovir disoproxil fumarate (TDF) 300 mg. See Table 4.1.

Study Assessments: Efficacy assessments will include plasma HIV-1 RNA measurements. Safety Assessments will include blood chemistry and hematology, ECGs, Physical Exams and Vital Signs, and assessment of non-serious AEs, SAEs and AEs leading to discontinuation.

Statistical Considerations:

Sample Size:

This is an estimation study, without statistical testing, and hence there are no power considerations. It is expected that the response rate for the primary endpoint will be somewhere around 80%. With this response rate, and 50 subjects per arm, an exact 95% confidence interval would run from roughly 66% to 90%.

Endpoints:

Primary Endpoint

The primary endpoint is the proportion of subjects with plasma HIV-1 RNA < 40 c/mL at Week 24. This will be assessed with the FDA snapshot algorithm. This uses the last on-treatment plasma HIV-1 RNA measurement within an FDA-specified visit window to determine response.

Secondary Endpoints

- The antiviral efficacy will be determined by the proportion of subjects with plasma HIV-1 RNA < 40 c/mL at Weeks 48 and 96 using the FDA snapshot algorithm
- The antiviral efficacy will also be assessed by the proportion of subjects with plasma HIV-1 RNA < 200 c/mL at Weeks 24, 48, and 96 using the FDA snapshot algorithm approach with positive response defined as HIV-1 RNA < 200 c/mL
- The emergence of HIV drug resistance among samples selected for drug resistance testing will be assessed using the most recent version of the IAS-USA list of HIV-1 drug resistance mutations
- Changes from baseline in log10 HIV-1 RNA and in CD4+ T-cell counts, and changes in the percentage of CD4+ T-cells over time will be assessed using on-treatment laboratory results and pre-specified visit windows
- The frequency of SAEs and AEs leading to discontinuation (DC) will be tabulated directly from the case report forms (CRFs). The summary will count the number of subjects that have at least one event
- The occurrence of new AIDS defining events (CDC Class C events) will be tabulated from the CRFs. The summary will count the number of subjects that have at least one event.
- The steady-state plasma PK of BMS-955176 will be assessed using the intensive PK data, collected at Week 2 from a subset of subjects.

Approved v3.0 930087119 3.0

Analyses:

- An interim analysis will be conducted when approximately 50% of the subjects have completed 24 weeks of therapy. Access to the unblinded data will be restricted to a limited number of BMS and vendor personnel involved in the development of models for population pharmacokinetics, exposure-response relationships, and viral kinetics.
- An interim analysis will be conducted after the last randomized subject is on study therapy for 24 weeks
  a) At Week 24, a Time to Loss of Virologic Response (TLOVR) analysis will be conducted as a sensitivity analysis that compliments the snapshot analysis.
- An interim analysis will be conducted after the last randomized subject is on study therapy for 48 weeks
- The final analysis will be conducted after the last randomized subject is on study therapy for 96 weeks

Approved v3.0 930087119 3.0

TABLE OF CONTENTS

TITLE PAGE 1
DOCUMENT HISTORY 3
SYNOPSIS 5
TABLE OF CONTENTS 11
1 INTRODUCTION AND STUDY RATIONALE 15
1.1 Study Rationale 15
1.1.1 Rationale to support study design 15
1.1.2 Rationale to support dose selection 16
1.1.3 Rationale to support any drug combinations 19
1.2 Research Hypothesis 19
1.3 Objectives(s) 19
1.3.1 Primary Objective 19
1.3.2 Secondary Objectives 19
1.3.3 Exploratory Objectives 20
1.4 Product Development Background 20
1.4.1 Background Information BMS-955176 20
1.4.1.1 Mechanism of Action 20
1.4.1.2 Nonclinical studies 21
1.4.1.3 Clinical studies 26
1.4.2 Background Information Efavirenz 29
1.4.3 Background Information TDF/FTC 29
1.4.4 Drug-Drug Interactions 29
1.5 Overall Risk/Benefit Assessment 30
2 ETHICAL CONSIDERATIONS 33
2.1 Good Clinical Practice 33
2.2 Institutional Review Board/Independent Ethics Committee 33
2.3 Informed Consent 33
3 INVESTIGATIONAL PLAN 35
3.1 Study Design and Duration 35
3.1.1 Screening 35
3.1.2 Day 1/Baseline Visit 35
3.1.3 Optional Week 2 Intensive PK Visit 36
3.1.4 Visits Week 4 - 96 36
3.1.5 Selection of the Continuation Dose, and the Switch to open-label study medications 37
3.1.6 Post-dosing Follow-up Period 37
3.1.7 End of the study 38
3.2 Post Study Access to Therapy 40
3.3 Study Population 40
3.3.1 Inclusion Criteria 40
3.3.2 Exclusion Criteria 42
3.3.3 Women of Childbearing Potential 44
3.4 Concomitant Treatments 44
3.4.1 Prohibited and/or Restricted Treatments 44

Approved v3.0 930087119 3.0

3.4.2 Other Restrictions and Precautions 44
3.5 Discontinuation of Subjects following any Treatment with Study Drug 45
3.6 Post Study Drug Study Follow up 45
3.6.1 Withdrawal of Consent 46
3.6.2 Lost to Follow-Up 46
4 STUDY DRUG 47
4.1 Investigational Product 49
4.2 Non-investigational Product 49
4.3 Storage and Dispensing 49
4.4 Method of Assigning Subject Identification 49
4.5 Selection and Timing of Dose for Each Subject 50
4.5.1 Instructions for Dose Administration 50
4.5.1.1 General Instructions 50
4.5.1.2 Instructions for ALL SUBJECTS for dosing during the blinded portion of the study 50
4.5.1.3 Instructions for dosing during the fully open-label portion of the study 51
4.5.2 Dose Modifications 51
4.6 Blinding/Unblinding 51
4.7 Treatment Compliance 52
4.8 Destruction of Study Drug 52
4.9 Return of Study Drug 53
5 STUDY ASSESSMENTS AND PROCEDURES 54
5.1 Flow Chart/Time and Events Schedule 54
5.1.1 Retesting During Screening or Lead-in Period 60
5.2 Study Materials 60
5.3 Safety Assessments 61
5.3.1 Vital Signs and Physical Examinations 61
5.3.2 Adverse Events 61
5.3.3 Concomitant Medication Assessment 61
5.3.4 Electrocardiograms 61
5.4 Efficacy Assessments 62
5.4.1 Primary Efficacy Assessment 62
5.4.1.1 Guidelines for Confirmatory Testing of Plasma HIV-1 RNA and Resistance testing 62
5.4.1.2 Protocol Defined Virologic Failure 63
5.4.2 Secondary Efficacy Assessments 63
5.4.2.1 CD4+ and CD8+ T-Cells 63
5.4.2.2 Drug Resistance Testing 63
5.5 Pharmacokinetic Assessments 64
5.5.1 Intensive PK Assessment 64
5.5.2 Sparse PK Assessments 65
5.6 Biomarker Assessments 66
5.7 Outcomes Research Assessments 66
5.8 Other Assessments 66
5.9 Results of Central Assessments 66

Approved v3.0 930087119 3.0

6 ADVERSE EVENTS 67
6.1 Serious Adverse Events 67
6.1.1 Serious Adverse Event Collection and Reporting 68
6.2 Nonserious Adverse Events 69
6.2.1 Nonserious Adverse Event Collection and Reporting 69
6.3 Laboratory Test Result Abnormalities 70
6.4 Pregnancy 70
6.5 Overdose 70
6.6 Potential Drug Induced Liver Injury (DILI) 71
6.7 Other Safety Considerations 71
6.7.1 Toxicity Management 71
6.7.1.1 Management of Elevations in Liver Transaminases 71
6.7.1.2 Management of Renal Toxicity 72
6.7.1.3 Gastrointestinal Toxicity Evaluation and Management Plan 72
7 DATA MONITORING COMMITTEE AND OTHER EXTERNAL COMMITTEES 75
8 STATISTICAL CONSIDERATIONS 75
8.1 Sample Size Determination 75
8.2 Populations for Analyses 75
8.3 Endpoints 76
8.3.1 Primary Endpoint(s) 76
8.3.2 Secondary Endpoint(s) 76
8.4 Analyses 76
8.4.1 Demographics and Baseline Characteristics 77
8.4.2 Efficacy Analyses 77
8.4.2.1 Primary Efficacy Analyses 77
8.4.2.2 Secondary Efficacy Analyses 78
8.4.3 Safety Analyses 78
8.4.4 Pharmacokinetic Analyses 79
8.4.4.1 Sparse Pharmacokinetic Analyses 79
8.4.4.2 PK/PD and PK/VK Analyses 79
8.4.5 Biomarker Analyses 79
8.4.6 Outcomes Research Analyses 79
8.4.7 Other Analyses (including Virologic Futility) 79
8.5 Interim Analyses 80
9 STUDY MANAGEMENT 80
9.1 Compliance 80
9.1.1 Compliance with the Protocol and Protocol Revisions 80
9.1.2 Monitoring 81
9.1.2.1 Source Documentation 81
9.1.3 Investigational Site Training 81
9.2 Records 81
9.2.1 Records Retention 81
9.2.2 Study Drug Records 82
9.2.3 Case Report Forms 82
9.3 Clinical Study Report and Publications 83

Approved v3.0 930087119 3.0

10 GLOSSARY OF TERMS 84
11 LIST OF ABBREVIATIONS 85
12 REFERENCES 92
APPENDIX 1 LISTINGS OF PROHIBITED AND PRECAUTIONARY THERAPIES 94
APPENDIX 2 AIDS-DEFINING DIAGNOSES 96
APPENDIX 3 DAIDS TOXICITY GRADES 102
APPENDIX 4 COCKCROFT-GAULT EQUATION TO CALCULATE SERUM CREATINE CLEARANCE 103
APPENDIX 5 LABORATORY ASSESSMENTS 104

Approved v3.0 930087119 3.0

1 INTRODUCTION AND STUDY RATIONALE

1.1 Study Rationale

HIV/AIDS continues to be a global epidemic. In 2013, approximately 35 million individuals were living with HIV; the number of newly infected individuals was ~2.1 million.[1] The major goals for antiretroviral (ARV) therapy are to achieve a maximal sustainable virologic suppression, restore and preserve immune function, reduce HIV related morbidity and mortality, and prevent transmission of HIV.[2]

In order to achieve these goals, several recommended ARV regimens are available for treatment-naïve HIV-1 infected patients that are Non-nucleoside Reverse Transcriptase Inhibitor (NNRTI), Protease Inhibitor (PI), or Integrase Inhibitor (INI) based. These agents are frequently combined with Nucleos(t)ide Reverse Transcriptase Inhibitors (NRTIs) to form fully active three-drug combination antiretroviral therapy (cART) for the treatment of ARV-naïve HIV-1 infected patients. However, existing ARVs do have safety (both short and longer term) and tolerability associated concerns. For example, some NRTIs have been associated with mitochondrial toxicity, some PIs have been associated with dyslipidemia, and some INIs have been associated with an increase in CPK levels. These factors, combined with the ongoing problem of drug resistance, form a continued need for new and efficacious agents with novel mechanisms of action (MOA) and favorable safety/tolerability profiles.

Given the aforementioned challenges with existing ARVs, the primary objective of this Phase 2b study is to evaluate the efficacy of three doses of BMS-955176 (a novel HIV maturation inhibitor) when given in combination with tenofovir/emtricitabine (TDF/FTC). By accomplishing this objective, AI468038 will provide supportive data to select a safe and therapeutic dose of BMS-955176 for subsequent development.

1.1.1 Rationale to support study design

This Phase 2b study seeks to build on preliminary data from the Phase 2a studies to accomplish the goal of studying the efficacy of three different, once-daily doses (60, 120, and 180 mg) of BMS-955176 when given in combination with TDF/FTC. By accomplishing this primary objective, this Phase 2b study will provide supportive data in the context of a therapeutic, safe, and tolerable dose of BMS-955176 for subsequent development.

The Phase 2b clinical trial design is in general agreement with guidance from both US and EU regulatory agencies.[3,4] Specifically, approximately 200 HIV-1 infected treatment-naive adults will be randomized 1:1:1:1 to one of four treatment arms and receive treatment with three fully active ARVs (see Section 1.1.3, Rationale to support any drug combinations).

In a recent clinical trial approximately 95% of randomized subjects were noted to be infected with HIV-1 clades B, C, and AE.[5] Based on in-vitro studies, BMS-955176 is expected to be active against a variety of HIV-1 clades albeit with EC50s approximately 2-3 fold less toward HIV-1 clade AE compared to clade B (see Section 1.4.1). However, since the Phase 2a study

Approved v3.0 930087119 3.0

AI468002 did not include any subjects infected with HIV-1 clade AE (see Section 1.4.1.3), this multinational Phase 2b study will cap the total number of HIV-1 clade AE infected subjects to approximately 8% (n ≈ 16 total, approximately 4 per treatment arm) and stratify randomization to ensure each treatment group has approximately the same number of clade AE infected subjects. The study will be double-blind until after the analysis of the primary endpoint (at Week 24).

The primary endpoint will be evaluated after all subjects reach Week 24. Subjects in Arm 4 will receive Efavirenz (EFV) in double-blind fashion until after the Week 24 primary endpoint has been analyzed. Based upon these results, an efficacious, safe, and tolerable dose of BMS-955176 will be chosen (see Section 3.1). Subjects in experimental arms 1-3 will subsequently be transitioned to the open-label continuation dose of BMS-955176 (see Section 3.1). TDF/FTC will be provided to all subjects in an open-label fashion. The study duration is expected to be 96 weeks in length to assess durability of response and longer term safety and tolerability.

1.1.2 Rationale to support dose selection

Phase 1 and Phase 2a clinical trials (AI468001[6] and AI468002[7]) investigating BMS-955176 utilized a spray-dried dispersion (SDD) suspension. However, this Phase 2b study will utilize a micronized crystalline (MC) tablet.

The doses of BMS-955176 of 60 mg, 120 mg, and 180 mg MC tablet are based upon modeling and simulation and formulation considerations. A population pharmacokinetic model was developed using single dose (10 mg - 120 mg SDD) and multiple dose (10 mg to 80 mg SDD) data in healthy subjects (AI468001) as well as multiple dose data in HIV-1 clade B-infected subjects (5 mg to 120 mg SDD for 10 days, AI468002). An exposure-response analysis was conducted using data from Part A of the Phase 2a clinical trial where HIV-1 clade B infected subjects received BMS-955176 monotherapy (Dose range: 5 - 120 mg) for 10 days. The exposure-response relationship was assessed via an E_{max} model using observed BMS-955176 steady state C_{min}. The primary endpoint was predicted maximum viral load decline from baseline.

Steady state BMS-955176 C_{min} values in HIV-infected subjects administered MC tablet with food were projected based on the following data and assumptions:

- Exposures in HIV-infected subjects are 35% less than normal healthy volunteers based on observations from studies AI468001 and AI468002
- Single dose data was projected to multiple dosing using accumulation indices from the AI468001 study
- BMS-955176 exposures from the MC tablet formulation with food were projected based on studies AI468001 and AI468034. The impact of food on the 60 mg MC tablet dose was assumed to be that observed for the 40 mg MC suspension formulation in AI468001. Exposures to BMS-955176 120 mg MC tablet with food were determined from observed data in Study AI468034 where BMS-955176 C24 increased approximately 70% when 120 mg MC tablet was given with a high fat meal, relative to fasted conditions. Finally, exposures to

Approved v 3.0 930087119 3.0

BMS-955176 180 mg MC tablet with food were assumed to be 1.5-times that of 120 mg MC tablet with food

Figure 1.1.2-1 depicts the simulated maximum viral load decline in HIV-infected subjects administered BMS-955176 MC tablet under fed conditions.

Figure 1.1.2-1: Simulated Maximum Viral Load Decline from Baseline Under Fed Conditions[1]

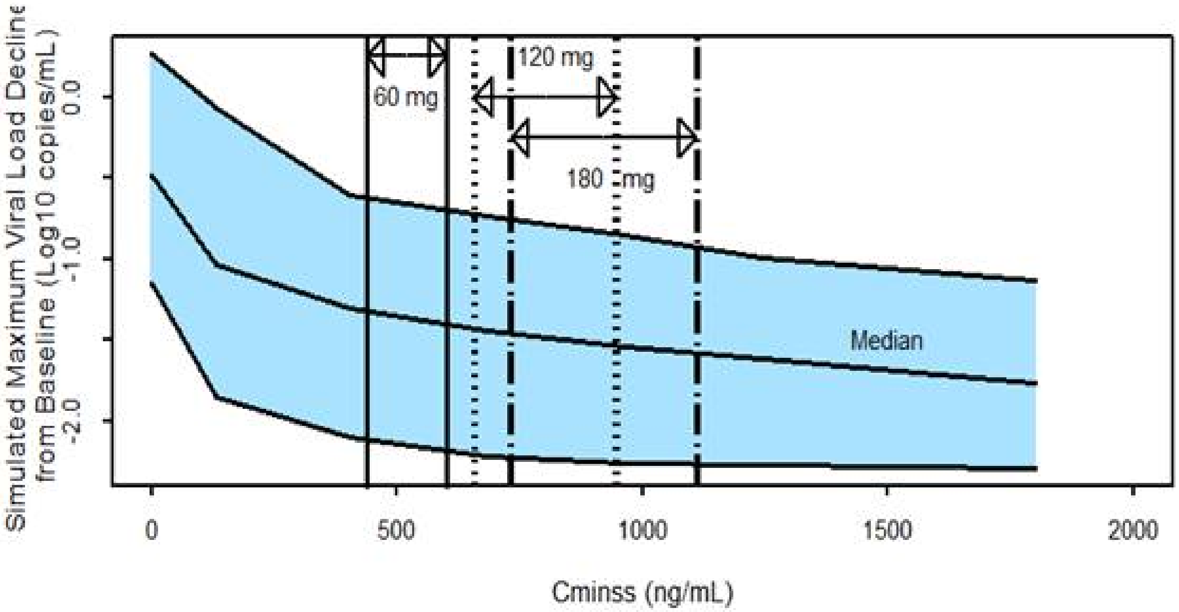

1 Solid lines are 10th and 90th percentiles and the median of simulated data, shaded area is the 90% confidence interval of simulated data, vertical solid lines are the 25th to 75th percentile of the simulated steady state BMS-955176 Cmin for the 60 mg MC tablet dose, vertical dotted lines are the 25th to 75th percentile of the simulated steady state BMS-955176 Cmin for the 120 mg MC tablet dose, and vertical dashed and dotted lines are the 25th to 75th percentile of the simulated steady state BMS-955176 Cmin for the 180 mg MC tablet dose.

While baseline EC_{90} was not a covariate included in the model due to the lack of significance; this covariate, among others, was considered marginally significant and it is possible this covariate will become important with additional data. This Phase 2b clinical trial will enroll subjects regardless of their viral susceptibility to BMS-955176; a range of baseline EC_{90}s are anticipated, and therefore the three doses above will give a wide range of exposures and coverage to assess against a range of baseline susceptibilities. Thus, the three doses of BMS-955176 may demonstrate varying rates of antiviral efficacy in Arms 1-3. Additionally, these three doses of BMS-955176 will allow for an assessment of safety and tolerability over a wide range of exposures by providing observed longer term clinical data. The combined efficacy and safety data from Arms 1-3 will support the process to select a dose for subsequent clinical development.

Approved v3.0 930087119 3.0

Although data from AI468002 Part C (in HIV-1 clade C infected subjects) was not included in the exposure-response assessment described above, BMS-955176 doses ≥ 40 mg SDD once daily demonstrated median maximal reductions in HIV-1 RNA > 1 $\log_{10}$ in both clade B and clade C HIV-1 infected subjects (see Section 1.4.1.3); thus, doses of BMS-955176 60 mg, 120 mg, and 180 mg are expected to yield a similar response in HIV-1 infected subjects of either clade.

Table 1.1.2-1 depicts the projected exposure multiples of BMS-955176 60 mg, 120 mg, and 180 mg MC tablet with food at the NOAEL for pre-clinical findings of interest.

Table 1.1.2-1: Exposure Multiples of BMS-955176 at NOAEL[a]

Species/ Study	NOAEL			Multiples		
	Dose (mg/kg/d)	Exposure	PK Parameter	60 mg	120 mg	180 mg
Rat/6-month (stomach histologic changes)	No NOAEL		AUC	---	---	---
Dog/9-month (stomach histologic changes)	1	AUC: 64.9 µg•h/mL	AUC	3×	2×	1×
Dog/1-month (heart rate)	20	Cmax: 17.8 µg/mL	Cmax	19×	10×	5×
Dog/ Cardiovascular telemetry (heart rate)	2	Cp: 1.93 µg/mL	Cmax	2×	1×	0.5×
Mouse/EFD	45	AUC: 197 µg•h/mL	AUC	10×	6×	3×

a Exposure multiple = animal value ÷ human value. Projected human Cmax values are 0.94, 1.79, and 3.61 µg/mL and steady state AUC values are 19.3, 35.8, and 69.2 µg•h/mL at 60, 120, and 180 mg in HIV-1 subjects receiving BMS-955176 MC tablets with high fat meal, respectively. High fat meal provides the highest exposure relative to other meal types or fasted state.

With regard to heart rate and the NOAEL of 1.93 µg/mL observed in the cardiovascular telemetry study in dogs (N=2), it is noted that projected exposure multiples are ≤ 1 at doses of 120 mg and 180 mg MC tablet in HIV-infected subjects. However, to date, there have been no clinically meaningful changes in heart rate observed in subjects treated with BMS-955176 up to 28 days (in Part B of the Phase 2a study). With regard to stomach histologic changes, no NOAEL could be established based on the 6-month rat study and the projected exposure multiples from the 9-month dog study are low (eg, 1-fold at the 180 mg MC tablet dose). Despite these preclinical findings, doses of BMS-955176 up to 180 mg MC tablet will provide exposures in this study which are expected to be generally safe and well tolerated based on existing clinical data (see Section 1.4.1).

Data from Study AI468034 demonstrated that BMS-955176 120 mg MC tablet AUC increased 53% when given with a high fat meal, relative to fasted conditions. Furthermore, preliminary data from Study AI468049 demonstrated that a light meal, a standard meal, and a high fat meal increased BMS-955176 180 mg MC tablet AUC 1.8-, 2.1-, and 2.5-fold, respectively, relative to

Approved v 3.0 930087119 3.0

fasted conditions. Taken together, these data suggest that exposures to BMS-955176 MC tablet at doses up to 180 mg increase in a linear fashion when given with food and that similar exposures are observed regardless of meal type. Thus, BMS-955176 will be administered with food in this study.

BMS-955176 MC tablet doses of 60 mg, 120 mg, and 180 mg with food are projected to provide a wide range of exposures to assess efficacy, tolerability, and safety when given in combination with TDF/FTC. Doses of BMS-955176 120 mg and 180 mg will minimize the potential risk to subjects with respect to lack of efficacy and the development of resistance. Despite overlap in exposures between BMS-955176 120 mg and 180 mg, assessment of both doses will provide observed data in the event that the BMS-955176 180 mg dose (Arm 3) has adverse effects and the BMS-955176 60 mg dose (Arm 1) has suboptimal antiviral response and potential subsequent resistance.

1.1.3 Rationale to support any drug combinations

The study co-administers one of three different, once-daily doses of BMS-955176 with TDF/FTC. Specifically, our proposed dose-finding arms 1-3 would allow treatment-naïve adults to be treated with three fully active ARVs. EFV is used as a comparator in Arm 4 since it is often employed in treatment-naïve adults' initial cART therapy. The rationale for using a backbone of TDF/FTC throughout all arms in the treatment-naïve patient population is based upon established safety, efficacy, and tolerability. Additionally, the potency of a backbone of TDF/FTC in combination with BMS-955176 (Arms 1-3) should allow for a differentiation of the efficacy of BMS-955176 at the three study doses informing the selection of a dose which is efficacious, safe, and tolerable (for use in both the open label phase of the study and subsequent development). Note both EFV and the components of TDF/FTC are recommended therapies for ARV treatment-naïve adults.[8,3]

1.2 Research Hypothesis

This Phase 2b study will demonstrate that at least one dose of BMS-955176 will be efficacious, safe, and tolerable for use in HIV-1 infected treatment-naïve adults

1.3 Objectives(s)

1.3.1 Primary Objective

- To evaluate antiviral efficacy of 3 doses (60, 120 and 180 mg) of BMS-955176, and EFV, each when given in combination with tenofovir/emtricitabine (TDF/FTC) by determining the proportion of treatment-naïve subjects with plasma HIV-1 RNA < 40 c/mL at Week 24

1.3.2 Secondary Objectives

- To assess the antiviral efficacy of BMS-955176 and EFV by determining the proportion of subjects with plasma HIV-1 RNA < 40 c/mL at Weeks 48 and 96

Approved v3.0 930087119 3.0

- To assess the antiviral efficacy of BMS-955176 and EFV by determining the proportion of subjects with plasma HIV-1 RNA < 200 c/mL at Weeks 24, 48, and 96
- To assess the emergence of HIV drug resistance among samples selected for drug resistance testing (according to criteria outlined in Section 5.4.1)
- To assess efficacy of BMS-955176 and EFV, by using mean changes from baseline in $\log_{10}$ HIV-1 RNA, CD4+ T-cell counts, and percentage of CD4+ T-cells
- To assess the safety and tolerability of BMS-955176 in treatment-naïve subjects by measuring frequency of SAEs and AEs leading to discontinuation
- To assess disease progression as measured by the occurrence of new AIDS defining events (CDC Class C events)
- To characterize the pharmacokinetics of BMS-955176 when co-administered with TDF and FTC in treatment-naïve HIV-1 infected subjects

1.3.3 Exploratory Objectives

- To assess the impact of baseline (pre-therapy) Gag polymorphisms on the efficacy of BMS-955176 in subjects in Arms 1-3 by determining the proportion of treatment-naïve subjects with plasma HIV-1 RNA < 40 c/mL and HIV-1 RNA < 200 c/mL, and the changes from baseline in $\log_{10}$ HIV-1 RNA at Weeks 24, 48 and 96, by baseline polymorphism
- To characterize the steady-state plasma PK of TDF and FTC when co-administered with BMS-955176 in treatment-naïve HIV-1 infected subjects. The effect of BMS-955176 on TDF and FTC will be assessed relative to the EFV reference arm
- To explore PK/PD and PK/viral kinetic (VK) relationships between BMS-955176 exposure and both efficacy and safety endpoints

1.4 Product Development Background

1.4.1 Background Information BMS-955176

1.4.1.1 Mechanism of Action

BMS-955176 is an HIV-1 maturation inhibitor (MI), a novel class of anti-HIV-1 drugs that prevents the maturation of HIV-1 virions by binding near a key structural element within the Gag polyprotein that is required for virion maturation and assembly. Maturation inhibitors block the last protease cleavage event between Gag protein segments designated as capsid (CA) protein p24 (p24) and spacer peptide 1 (SP1), resulting in the release of immature noninfectious virus particles. BMS-955176 has excellent potency and broad spectrum activity, and mechanism of action studies indicate that BMS-955176 is a true MI, with a mechanism of action distinct from current antiretroviral agents.[9]

Approved v 3.0 930087119 3.0

1.4.1.2 Nonclinical studies

Nonclinical Pharmacology and Microbiology

BMS-955176 specifically inhibits HIV-1 protease cleavage at the CA(p24)/SP1 junction within the Gag protein in both HIV-1-infected cells and purified HIV-1 Gag virus-like particles (VLPs). Radiolabeled BMS-955176 specifically binds to purified HIV-1 Gag VLPs, and its binding is dose-dependently inhibited by related MIs and is reversible. BMS-955176 does not directly inhibit HIV-1 protease nor bind to a small HIV-1 protease peptide substrate. These results indicate that BMS-955176 inhibits late in the HIV-1 life cycle by specific binding to immature capsid structures at or near the CA(p24)/SP1 junction, thereby inhibiting cleavage at that particular site. In cell culture, the range of values for the concentration producing 50% effect (EC50) of BMS-955176 against 7 common laboratory strains of HIV-1 was 1.6 to 10.5 nM (mean = 6.0 ± 3.5 nM). Using a reverse transcriptase readout, a phenotyping analysis of 93 subtype B viruses whose genotypes are representative of 96% of the diversity (found in the Los Alamos National Laboratory [LANL] database) in Gag sequences indicates that the mean EC50 of this cohort was 2.7 ± 1.9 nM, with a median value of 2.2 nM and a range between 0.6 to 12 nM. A similar analysis of 23 isolates of subtype C viruses found a mean EC50 of 6.1 ± 3.1 nM, a median value of 5.6 nM, and a range from 2.5 to 16 nM. When evaluated against clinical isolates in peripheral blood mononuclear cells (PBMCs), BMS-955176 exhibited a mean EC50 of 24 ± 24 nM against a cohort (N = 22) of subtype B viruses. Activity was also observed against viruses from subtypes A, C, D, F, and G, with average EC50 values for 96% of tested isolates (N = 41) between 5.9 and 87 nM. Clinical isolates from the CRF01_AE subtype were approximately 2- to 3-fold less susceptible to BMS-955176 (average EC50 77 nM, N = 7) viruses. BMS-955176 was active against 1 of 3 human immunodeficiency virus type-2 (HIV-2) isolates (EC50 = 15 nM). BMS-955176 retains complete activity against reverse transcriptase (RT), protease, and integrase inhibitor-resistant viruses, with EC50 values similar to wild-type (wt) viruses, while the potency of currently approved nucleotide/nucleoside reverse transcriptase inhibitors (NRTIs), non-nucleoside reverse transcriptase inhibitors (NNRTIs), protease inhibitors (PIs), and integrase inhibitors (INIs) was undiminished when tested against viruses with reduced susceptibility to BMS-955176. BMS-955176 maintained activity against a panel of PI-resistant isolates from PI-treated subjects harboring a variety of major and minor PI-resistance determinants in both protease and Gag.[10,11] Protein binding to 100% human serum (HS) was 86%, and in the presence of 40% HS supplemented with additional human serum albumin (HuSA) to match physiologic concentrations, BMS-955176 exhibited an approximately 4-fold reduction of antiviral activity. Selection for resistance to BMS-955176 in cell culture identified changes that map to amino acids adjacent to the CA(p24)/SP1 cleavage site. These include an amino acid substitution of A364V or a combination of V362I with secondary changes (V370A, A374P or I376V). In vitro, virus with the A364V change exhibited a drastic loss of susceptibility to BMS-955176 (>100-fold), while the V362I plus secondary change-containing viruses were generally less sensitive to BMS-955176 (median EC_{50} 25 nM, range 7.1 to 167 nM). In 2-drug combination studies with representative drugs from NRTI, NNRTI, PI, and INI classes, all

Approved v3.0 930087119 3.0

combinations produced additive to synergistic effects, suggesting that BMS-955176 should be amenable for use in combination with any of these agents.

Bevirimat (BVM), a first-generation MI, demonstrated proof of concept and dose-dependent anti-HIV-1 potency in both Phase 1 and Phase 2 clinical studies. Patients infected with HIV-1 sensitive to BVM demonstrated an approximate 1.2 log_{10} decline in HIV-1 RNA. However, approximately 50% of patients harboring naturally occurring polymorphisms located close to the CA(p24)/SP1 cleavage site showed a significantly reduced response to BVM treatment. In addition, BVM exhibited a large reduction in antiretroviral activity in the presence of human serum. BMS-955176 was developed to address the key flaws of BVM by providing improved coverage of BVM-resistant polymorphic variants and improved potency in serum. BMS-955176 has been shown to be active against viruses with resistance from all marketed ARVs and to possess a low serum effect. Development of BMS-955176 could potentially lead to novel HIV-1 treatment regimens in HIV-1 infected patients.

Nonclinical Pharmacokinetics

The absolute oral bioavailability of BMS-955176 was low (3.89% to 26.8%) in all preclinical species (mice, rats, dogs, and monkeys). In the dog, though there was a positive food effect and no pH dependent absorption, upon repeat dosing a less than dose-proportional increase in exposure was observed. BMS-955176 distributed preferentially into the duodenum, liver, and lymph nodes with little penetration into the brain. Protein binding was 86.1% in human serum and 78% to 94% in animal sera

In human in vitro systems, the metabolism of BMS-955176 was primarily mediated via cytochrome P450 (CYP)3A4. In vivo in rats, dogs, and monkeys, BMS-955176 was the predominant drug-related component in plasma following a single oral dose of BMS-955176. BMS-955176 was eliminated principally via metabolism followed by excretion in bile with little renal excretion.

In vitro, BMS-955176 was an inhibitor of CYP2C8 (concentration at which 50% inhibition observed [IC50] = 28.5 μM), CYP3A4 (IC50 = 32 μM), and uridine diphosphate glucuronosyltransferase (UGT)1A1 (IC50 = 20 μM) enzymes. No P-gp inhibition or time-dependent inhibition of CYPs was observed. BMS-955176 was not an inducer of CYP1A2, CYP2B6, or CYP3A4. The steady state C_{max} of BMS-955176 180 mg tablet in HIV-infected patients with food is projected to be approximately 5.2 μM. Thus, the potential exists for BMS-955176 to inhibit CYP2C8, CYP3A4, and/or UGT1A1 in vivo and increase exposures to co-administered drugs that are metabolized by these enzymes. Furthermore the potential exists for drug-drug interactions (DDI) if BMS-955176 is co-administered either with an inhibitor or inducer of CYP3A4 or P-gp.

BMS-955176 was a substrate of mouse P-glycoprotein (P-gp) based on higher bioavailability in P-gp knock-out mice when BMS-955176 was co-administered with elacridar, a potent inhibitor of P-gp and breast cancer resistance protein (BCRP). BMS-955176 could not be reliably assessed as a substrate for human P-gp due to nonspecific binding and low solubility. In vitro, BMS-955176 inhibited organic anion transporting polypeptide (OATP)1B1 and OATP1B3

Approved v3.0 930087119 3.0

(IC50 5.3 and 4 μM, respectively), but was not an inhibitor of P gp, sodium taurocholate cotransporting polypeptide (NTCP), organic anion transporter (OAT)1, OAT3, multiple drug-resistance protein (MRP)2, and bile salt export pump (BSEP). These findings suggest a potential for DDI between BMS-955176 (as the perpetrator) and substrates of OATP1B1 and OATP1B3, but not with those of P-gp, NTCP, OAT1, OAT3, MRP2, and BSEP. Furthermore, the potential exists for drug-drug interactions (DDI) if BMS-955176 is co-administered either with an inhibitor or inducer of CYP3A4 or P-gp. Preliminary data indicate that BMS-955176 does not inhibit OCT2, a transporter that is inhibited by dolutegravir (DTG), a drug with which BMS-955176 is planned to be co-administered.

Nonclinical Toxicology

The toxicity profile of BMS-955176 was evaluated in single- and repeat-dose toxicity, genotoxicity, phototoxicity, safety pharmacology, sensitization, reproductive toxicity and embryo-fetal development studies. The scope of the toxicologic evaluation for BMS-955176 supports its proposed clinical use for HIV-1 infection. Unless otherwise mentioned, all animal studies were dosed by the oral route with an aqueous methylcellulose suspension of a BMS-955176 spray-dried dispersion (SDD).

BMS-955176 was not phototoxic, mutagenic, or clastogenic in vitro and was not genotoxic in a rat micronucleus assay at ≤ 300 mg/kg/day (AUC ≤ 279 μg·h/mL). BMS-955176 was not a skin sensitizer in the local lymph node assay in the mouse. BMS-955176 had a low potential (IC_{50} or EC_{50} > 5 μM [> 3.45 μg/mL]) for in vitro off-target interactions on a broad range of enzymes, transporters, and receptors, including cardiac ion channels.

In safety pharmacology evaluations in rats, there were no respiratory findings and no direct central nervous system (CNS) findings. Decreases in motor activity, arousal, and rearing were considered secondary to general toxicity (ie, body weight decreases).

Cardiovascular safety pharmacology evaluations were conducted in rabbits, rats, and dogs. In the definitive oral single-dose cardiovascular safety study in conscious telemeterized dogs, blood pressure and electrocardiogram were unaffected at ≤ 20 mg/kg; however, increases in heart rate (mean 33% to 57% of pretest vehicle) were observed at 8 and 20 mg/kg. The increase in heart rate at these doses was primarily due to increases in 2 dogs at each dose that had higher plasma concentrations (≥ 12.83 μg/mL) relative to the dogs without effects on heart rate (≤ 6.81 μg/mL). The no-observed-effect level (NOEL) for cardiovascular effects in dogs was 2 mg/kg (plasma concentration of 1.93 μg/mL). Importantly, there was no change in heart rate at ≤ 20 mg/kg/day at higher plasma concentrations (C_{max} ≤ 17.8 μg/mL) in the 1-month study in dogs (below).

Taken together, BMS-955176 has low potential for respiratory, CNS, and cardiovascular effects and no cardiovascular effects have been observed in humans to date.

Two-week, 1-month, and 6-month studies were conducted in rats. As the 2-week study was of limited scope, only the 1-month and 6-month studies are presented in this summary. BMS-955176 was administered for 1 month at doses of 30, 100, or 300 mg/kg/day. While there was no mortality at ≤ 100 mg/kg/day, the high dose of 300 mg/kg/day was associated with

Approved v3.0 930087119 3.0

pronounced signs of clinical toxicity and early euthanasia of all the rats at that dose level on Days 8 to 9. The dose of 30 mg/kg/day was tolerated. The intermediate dose of 100 mg/kg/day (AUC 357 μg·h/mL) resulted in dose-limiting toxicity including persistent reduction in food consumption and body weights. A number of minor hematology (including red cell parameter changes with no consistent effect on the erythron) and serum chemistry changes (including increased alkaline phosphatase and alanine aminotransferase) without correlating histologic liver findings) occurred at 30 and 100 mg/kg/day; these changes were considered not adverse due to small magnitude, occurrence only in 1 sex, and lack of microscopic correlates, and most were secondary to decreases in food consumption and body weight. Dose-related gastrointestinal toxicity was primarily characterized by morphologic changes in the stomach at 100 mg/kg/day and the stomach and small and large intestines at 300 mg/kg/day. At the end of the 2-week postdose recovery period, there was complete recovery of all BMS-955176-related findings at 30 mg/kg/day. At 100 mg/kg/day, all findings recovered with the exception of increased red cell distribution width in females, minimally higher (1.94x) ALT activity in 1 male without any histologic correlates, and decreased mean prostate gland (with seminal vesicles) weights. The low dose of 30 mg/kg/day (AUC 113.5 μg·h/mL) was considered the no-observed-adverse-effect level (NOAEL) because the body weight and food consumption changes were minimal and transient and there were no BMS-955176-related morphologic changes.

In a 6-month oral toxicity study in rats with 1-month recovery period, BMS-955176 was administered at doses of 10, 25, or 50 mg/kg/day. BMS-955176-related effects were similar to those observed in the 1-month rat study and occurred at all doses (≥ 10 mg/kg/day; AUC ≥ 71 μg•h/mL). Findings included decreased body weight, food consumption, and in the stomach, minimal to marked atrophy involving both parietal and chief cells, minimal to mild single-cell necrosis and minimal regeneration in the glandular mucosa, which were partially reversible at the end of the 1-month recovery period. A NOAEL was not established in this study.

Five-day, 1-month, and 9-month repeat-dose studies were conducted in dogs. As the 5-day toxicokinetics and tolerability studies were of limited scope, only the 1-month and 9-month studies are presented here. In the 1-month study, BMS-955176 was administered at doses of 2, 8, or 20 mg/kg/day. Increased incidences of sporadic vomiting and liquid, yellow, and/or mucoid feces occurred at all doses, but had no apparent effect on the overall health of these animals. At 20 mg/kg/day, additional findings included occasional decreases in food consumption in a few animals, loss of body weight (up to 8%) in 2 females, a minimal increase in serum ALT activity (2.10× pretest) in 1 female with no microscopic correlates, and minimal single-cell necrosis of stomach glandular epithelium. All BMS-955176-related changes were fully reversible by the end of the 2-week recovery period. The dose of 8 mg/kg/day was considered a NOAEL (AUC 219.5 μg·h/mL) since the sporadic clinical observations had no adverse effects on the general health of the animals and there were no BMS-955176-related morphologic changes.

In a 9-month oral toxicity study in dogs with 1-month recovery period, BMS-955176 was administered at 0 (vehicle), 1, 3, or 10 mg/kg/day. BMS-955176-related effects were similar to those observed in the 1-month dog study and occurred at doses ≥ 3 mg/kg/day

Approved v 3.0 930087119 3.0

(AUC ≥ 135 µg•h/mL). Findings included salivation (only males at 10 mg/kg/day), fur thinness (males), thin appearance, and abnormal feces (yellow, liquid, pale and/or mucoid) that occurred sporadically throughout the study; increases in mean food consumption; minimal to marked chief cell depletion in the glandular stomach. Additional findings at 10 mg/kg/day included thin appearance that correlated with decreases in body weight and food consumption; occasional vomitus in males; in the stomach, minimal to moderate mucous cell hyperplasia (often associated with glandular dilatation) correlating with increased thickness macroscopically (males only) and minimal to marked parietal cell depletion and single-cell necrosis of glandular epithelial cells; and increases in serum gastrin values (1.31 to 4.56× highest control value) for several dogs that may have reflected the reductions in gastric parietal cells. The NOAEL was 1 mg/kg/day (AUC 64.9 µg•h/mL).

The embryo-fetal development (EFD) studies were conducted in 3 species (rabbits, rats, and mice) instead of the standard 2 species due to poor maternal tolerability and inability to achieve adequate systemic exposures in rabbits.

In a definitive EFD study in pregnant mice, BMS-955176 was administered at doses of 15, 45, or 150 mg/kg/day from gestation day (GD) 6 through 15. BMS-955176 was a selective developmental toxicant in mice. Dose of 100 mg/kg/day was associated with an increase in embryo-fetal lethality (cumulative postimplantation losses of 11.5%, relative to 3.9% in control litters, attributed to increased incidences of dead fetuses, early resorptions and late resorptions). Cleft palate and exencephaly were observed in a few fetuses; additionally, marginal reductions in fetal body weight (5% relative to control values) were observed. There was no maternal toxicity at any dose tested. The developmental NOAEL was 45 mg/kg/day (AUC 213 µg h/mL).

In a definitive EFD study in pregnant rats, BMS-955176 was administered at doses of 10, 30, or 100 mg/kg/day from GD 6 through 15. BMS-955176 was not a selective developmental toxicant. Developmental toxicity (reduced fetal body weights, increases in fetal alterations, and reduced fetal ossification) occurred only at 100 mg/kg/day; whereas, maternal toxicity (clinical observations, reduced body weights, and reduced food consumption) was observed at ≥ 30 mg/kg/day. The developmental NOAEL was 30 mg/kg/day (AUC 114 µg•h/mL).

In an EFD study in pregnant rabbits, BMS-955176 was administered at a dose of 80 mg/kg/day from GD 7 through 19. BMS-955176 was not a developmental toxicant in rabbits at 80 mg/kg/day (AUC 3.26 µg•h/mL), at which reductions in maternal food consumption and weight gain were observed.

In the fertility and early embryonic development study in rats, BMS-955176 was evaluated at doses of 10, 30, or 100/60 mg/kg/day in males and females. BMS-955176 did not affect reproduction or early embryonic development at doses ≤ 100 mg/kg/day that produced overt toxicity. The reproductive NOAEL was 100/60 mg/kg/day (AUC 210 µg•h/mL) in male rats and 100 mg/kg/day (AUC 458 µg•h/mL) in female rats.

Overall, results from the nonclinical toxicology studies demonstrate that BMS-955176 has a low potential for cardiovascular effects, is toxic to the gastrointestinal tract, and is a selective

Approved v 3.0 930087119 3.0

developmental toxicant. Clinical monitoring of vital signs (heart rate, systolic, and diastolic blood pressure) and for gastrointestinal adverse events (AEs) (eg, nausea, vomiting, diarrhea, or fecal changes), along with screening for potential renal tubular injury, have not indicated any potential for these AEs in Phase 1 or proof of concept (POC) studies in humans. Clinical protocols will ensure that appropriate contraceptive measures will be followed to minimize the risk of pregnancy while enrolling women of child-bearing potential (WOCBP) and males subjects who are sexually active with WOCBP (see Section 3.3.1 Inclusion Criteria).

1.4.1.3 Clinical studies

Phase 1

The safety, tolerability, and PK of BMS-955176 were evaluated in a randomized, double-blind, placebo-controlled, sequential single ascending dose (SAD, 10-120 mg) and multiple ascending dose (MAD, 10-80 mg QD for 14-28 days) study in healthy subjects (AI468001). No SAEs, deaths, or discontinuations related to study drug occurred. No clinically meaningful trends were observed in vital signs, physical exam findings, laboratory values, or ECGs. Following single-dose and multiple-dose administration of BMS-955176, a slightly less than dose-proportional increase in C_{max} and AUC(INF) was observed over the dose ranges studied. Steady state was reached in approximately 7 days following multiple-dose once daily administration of BMS-955176. The half life (T-HALF) of BMS-955176 is approximately 35 hours.

Study AI468034 assessed the relative bioavailability and dose proportionality of BMS-955176 MC tablet - the formulation that will be used in the current study. Relative to 80 mg SDD suspension, the bioavailability of BMS-955176 120 mg MC tablet was approximately 23% lower. Furthermore, consistent with the low solubility of BMS-955176, considerable overlap in exposures was observed between 60 mg, 120 mg, and 180 mg MC tablet, when given under fasted conditions. The impact of food on exposures to BMS-955176 120 mg MC tablet was assessed in Study AI468034 as well; a high fat meal increased BMS-955176 AUC approximately 50% with negligible impact on BMS-955176 C_{max}.

Study AI468049 assessed the impact of a light meal, a standard meal, and a high fat meal on the PK of BMS-955176 180 mg MC tablet. Preliminary results demonstrate that, relative to fasted conditions, BMS-955176 C_{max} is increased approximately 2-fold with all three meal types, while BMS-955176 AUC increased approximately 1.8-, 2.1-, and 2.5-fold with a light meal, a standard meal, and a high fat meal, respectively. These results, taken together with those from AI468034 described above, demonstrate that the impact of food on exposures to BMS-955176 is dose-dependent with the degree of impact increasing with increasing dose. Preliminary safety results from AI468049 indicate that GI adverse events (eg, nausea, vomiting, loose stools) only occurred in the fed arms (where the BMS-955176 exposures were higher) relative to the fasted arms.

Approved v3.0 930087119 3.0

Phase 2a

A randomized, double-blind, placebo-controlled proof of concept study in HIV subjects has completed enrollment and is undergoing analysis (AI468002). The three parts of this study were: 1) Part A evaluated doses of 5, 10, 20, 40, 80, and 120 mg of BMS-955176 (SDD suspension) given for 10 days in HIV-1 clade B infected subjects, 2) Part B compared the antiviral activity of BMS-955176 (SDD suspension) administered with ATV (with or without RTV) against standard of care (TDF + FTC + ATV/r) for 28 days in HIV-1 clade B infected subjects, and 3) Part C evaluated BMS-955176 40 and 120 mg (SDD suspension) given for 10 days in HIV-1 clade C infected subjects. See Table 1.4.1.3-1 for baseline demographics.

Preliminary results from the Phase 2a study (AI468002) in HIV-1 (clade B and C only) infected adults showed that at effective doses, a maximum median reduction in HIV-1 RNA ranging from 1.3 to 1.7 $\log_{10}$ was observed. In the Phase 2b study BMS-955176 doses estimated to provide similar exposure to effective doses in the Phase 2a study will be used. Moreover, when BMS-955176 was combined with ATV ± RTV, these combinations resulted in maximum median declines in HIV-1 RNA ranging from 1.9 to 2.2 $\log_{10}$ (see Table 1.4.1.3-2). These results are generally similar to the antiviral effect demonstrated by other classes of ARVs in short-term trials, and thus BMS-955176 should contribute substantially with other ARVs to form an effective cART regimen. Lastly, preliminary safety data show acceptable safety and tolerability across all Phase 2a arms. Most AEs were Grade 1-2 and were most frequently due to an indirect hyperbilirubinemia; the levels seen with BMS-955176 and ATV/r were similar to those seen with ATV/r combined with TDF/FTC. The arms containing BMS-955176 and unboosted ATV had bilirubin levels that were approximately half of those observed in the arms containing ATV/r. Last, arms containing BMS-955176 alone did not show elevated bilirubin levels. Many of these events occurred in subjects who were randomized to an arm containing BMS-955176 and ATV. Of the Grade 2-4 related AEs, many were due to headache and an increase in hyperbilirubinemia. Many of the AEs of hyperbilirubinemia occurred in subjects also receiving ATV.

Table 1.4.1.3-1: Phase 2a Baseline Demographics and Characteristics of Subjects (Preliminary Results)

Treatment Arm	Subjects (n)	Median age	Male	White	Median HIV RNA ($\log_{10}$ c/ml)	Median CD4 (cells/mm^3)
**Part A (Clade B, 10 days monotherapy)**						
BMS-955176 5 mg	8	43.5	8 (100)	6 (75.0)	4.09	437
BMS-955176 10 mg	8	39	7 (87.5)	7 (87.5)	4.02	539
BMS-955176 20 mg	8	33	8 (100)	8 (100)	3.59	512
BMS-955176 40 mg	8	38	8 (100)	8 (100)	4.03	536

Approved v 3.0 930087119 3.0

Table 1.4.1.3-1: Phase 2a Baseline Demographics and Characteristics of Subjects (Preliminary Results)

Treatment Arm	Subjects (n)	Median age	Male	White	Median HIV RNA (log_{10} c/ml)	Median CD4 (cells/mm^3)
BMS-955176 80 mg	8	31.5	8 (100)	8 (100)	3.82	504
BMS-955176 120 mg	8	37.5	8 (100)	8 (100)	3.84	498
Placebo	12	36	12 (100)	12 (100)	3.98	458
**Part B (Clade B, 28 days therapy)**						
BMS-955176 40 mg + ATV 400 mg	8	32.5	8 (100)	6 (75)	4.04	581
BMS-955176 40 mg + ATV 300 mg + RTV 100 mg	8	34	8 (100)	8 (100)	4.45	480
BMS-955176 80 mg + ATV 400 mg	8	31.5	8 (100)	7 (87.5)	4.15	549
Truvada® + ATV 300 mg + RTV 100 mg	4	32.5	4 (100)	4 (100)	4.12	427.5
**Part C (Clade C, 10 days monotherapy)**						
BMS-955176 40 mg	7	35	4 (57.1)	2 (28.6)	4.53	554
Placebo	2	38.5	2 (100)	0 (0)	3.78	304

Table 1.4.1.3-2: Maximum Decline Log_{10} HIV-1 RNA (Preliminary Results)

Treatment	Mean	S.D.	Median	Max	Min
**Part A (Clade B, 10 days monotherapy)**					
BMS-955176 5 mg	-0.49	0.217	-0.498	-0.78	-0.22
BMS-955176 10 mg	-1.05	0.351	-0.976	-1.76	-0.64
BMS-955176 20 mg	-1.17	0.645	-1.115	-2.12	-0.13
BMS-955176 40 mg	-1.55	0.352	-1.701	-1.88	-0.93
BMS-955176 80 mg	-1.52	0.257	-1.555	-1.82	-1.04
BMS-955176 120 mg	-1.53	0.478	-1.654	-2.07	-0.83
Placebo	-0.48	0.581	-0.381	-1.46	0.56
**Part B (Clade B, 28 days therapy)**					
BMS-955176 40 mg + ATV 400 mg	-1.89	0.273	-1.858	-2.37	-1.49
BMS-955176 40 mg + ATV 300 mg + RTV 100 mg	-2.22	0.676	-2.202	-3.52	-1.24

Approved v3.0 930087119 3.0

Table 1.4.1.3-2: Maximum Decline Log_{10} HIV-1 RNA (Preliminary Results)

Treatment	Mean	S.D.	Median	Max	Min
BMS-955176 80 mg + ATV 400 mg	-2.3	0.307	-2.228	-2.68	-1.87
Truvada® + ATV 300 mg + RTV 100 mg	-2.41	0.495	-2.39	-3.04	-1.83
**Part C (Clade C, 10 days monotherapy)**					
BMS-955176 40 mg	-1.5	0.439	-1.285	-2.03	-1.04
Placebo	0.12	0.141	0.12	0.02	0.22

The pharmacokinetics of BMS-955176 were assessed in HIV-1 infected subjects in AI468002. Overall, exposures to BMS-955176 are approximately 30% to 35% lower in HIV-1-infected subjects compared to healthy subjects administered the same doses and formulation of BMS-955176. Furthermore, exposures to BMS-955176 increased in a generally linear fashion up to 40 mg, with a less than dose proportional increase in exposures between 40 mg and 80 mg, and considerable overlap in exposures between 80 mg and 120 mg.

1.4.2 Background Information Efavirenz

EFV is a non-nucleoside reverse transcriptase inhibitor indicated in combination with other ARVs for the treatment of HIV-1 infection. The most common AEs include impaired concentration, abnormal dreams, rash, dizziness, nausea, headache, fatigue, insomnia, and vomiting. Clinicians are warned about serious psychiatric symptoms, nervous system symptoms, and rash.

EFV is recommended to be administered on an empty stomach. For more information concerning EFV, please refer to the EFV/Sustiva®SmPC or EFV/Sustiva®USPI.[12]

1.4.3 Background Information TDF/FTC

The combination of tenofovir disoproxil fumarate (TDF) 300 mg and emtricitabine (FTC) 200 mg, both nucleo(t)side analog HIV-1 reverse transcriptase inhibitors, are indicated in combination with other antiretroviral agents for the treatment of HIV-1. The most common adverse reactions include diarrhea, nausea, fatigue, headache, dizziness, depression, insomnia, abnormal dreams, and rash. Clinicians are warned about new onset worsening renal impairment and decreases in bone mineral density.

TDF/FTC can be administered with or without food. For more information concerning TDF/FTC, please refer to the TDF/FTC SmPC or USPI.[13]

1.4.4 Drug-Drug Interactions

In AI468001, coadministration of BMS-955176 as a single dose following two doses of 100 mg RTV resulted in an approximate 48% increase in BMS-955176 AUC(INF), consistent with

Approved v3.0 930087119 3.0

inhibition of CYP3A4 and/or P-gp. Multiple-dose administration of BMS-955176 with daily 400 mg ATV and a standard meal for 14 days resulted in a modest (~25%) increase in the BMS-955176 AUC(TAU).

Study AI468005 assessed the two-way interaction between BMS-955176 40 mg (administered as an SDD suspension) and TDF at steady state in healthy subjects. Relative to administration of each drug alone, neither BMS-955176 nor TDF exposures were meaningfully impacted upon coadministration.

Study AI468041 assessed the impact of BMS-955176 80 mg (administered as an SDD suspension) on the pharmacokinetics of the components of a combined oral contraceptive containing ethinyl estradiol (EE) and norgestimate (NGM). Exposures to both EE and norelgestromin (NGMN), the active metabolite of NGM were reduced in the presence of BMS-955176. Furthermore, one subject had a serum progesterone level > 300 ng/dL while BMS-955176 and the oral contraceptive were concomitantly administered, indicative of ovulation and contraceptive failure.

Finally, in vitro data suggest that BMS-955176 may inhibit OATP1B1 and OATP1B3 and exposures to substrates of these transporters, such as HMG-CoA reductase inhibitors, may increase when co-administered with BMS-955176.

1.5 Overall Risk/Benefit Assessment

The preclinical and clinical safety data demonstrate that BMS-955176 administered at doses in this Phase 2b study (60, 120, and 180 mg) should be well tolerated without a major clinically relevant impact on safety. Moreover, there have been no identified safety risks from completed/ongoing clinical studies to date.

The preclinical toxicology studies demonstrate two potential risks to subjects:

First, BMS-955176 is a selective developmental toxicant. Developmental toxicity (skeletal alterations in rats; cleft palate and reduced fetal body weights in mice) were observed in embryofetal development studies. In order to address this concern, subjects will be required to use two methods of contraception (as described in Section 3.3.1 Inclusion Criteria) and undergo routine urine pregnancy testing (as described in the T&E Tables in Section 5.1). Furthermore, due to results from Study AI468041 that demonstrates reduced exposures to the components of a combination oral hormonal contraceptive containing ethinyl estradiol and norgestimate when given concomitantly with BMS-955176, oral hormonal contraceptives cannot be used as a method of contraception by WOCBP in this study.

Second, single or repeat oral doses of BMS-955176 were associated with sporadic vomiting in dogs and unformed and/or liquid feces in rats and dogs. In rats at ≥ 10 mg/kg/day there were decreases in body weight and food consumption; in the stomach there was atrophy involving both parietal and chief cells, single-cell necrosis and regeneration in the glandular mucosa, and modest increases in serum gastrin values. At higher doses (≥ 100 mg/kg/day) in rats there were additional findings in the intestines (distended jejunum, ileum, and cecum; hyperplasia of the

Approved v3.0 930087119 3.0

crypt epithelium in the jejunum; ulcers and erosions in the cecum; and decreased mucosal cell extrusion and increased mucus in the colon).

Similar gastric changes were seen in dogs. At 20 mg/kg/day there was single-cell necrosis of the stomach glandular epithelium. At ≥ 3 mg/kg/day gastric changes showed chief cell depletion. At 10 mg/kg/day changes in the stomach included: mucous cell hyperplasia correlating with increased thickness macroscopically, parietal cell depletion, single-cell necrosis of glandular epithelial cells, and modest increases in serum gastrin values. Unlike the rats, no changes were observed elsewhere in the alimentary canal including the gastroesophageal junction and the duodenum. There was no evidence of macrocytosis. Measurement of Total Protein and Albumin revealed no clinically relevant changes. The stomach histologic findings were BMS-955176 dose- and duration dependent. In the 1-month studies, vomiting and fecal changes stopped soon after dosing cessation, and microscopic lesions in the stomach and/or intestines reversed completely within a 2-week treatment-free period. In the 6 month study in rats and the 9-month study in dogs, microscopic lesions in the stomach partially recovered after a 1 month treatment free period. The NOAEL was 1 mg/kg/day (AUC 64.9 mg•h/mL) in the 9-month study in dogs, and was not established in the 6-month study in rats. Investigative studies for gastric toxicity in rats and dogs indicated similar findings with both SDD and MC forms, and with no clear evidence that the gastric toxicity is a direct local effect of BMS-955176. The mechanism and clinical relevance of these gastrointestinal findings is unknown at present (see below).

A Phase 1 study (AI468001) in healthy volunteers evaluated single and multiple doses of BMS-955176 for 14-28 days both alone and in certain arms, in combination with ATV or RTV. Overall the safety data demonstrated that BMS-955176 was generally safe and well tolerated. A Phase 2a (AI468002) study in HIV-1 infected adults evaluated several doses of BMS-955176 given alone or in combination with ATV ± RTV for 10-28 days. The preliminary results show acceptable safety and tolerability across all arms. There were no deaths, SAEs, or AEs leading to discontinuation. There were no clinically relevant changes in vital signs, lab parameters, or EKGs. Most of the AEs were Grade 1-2 and were most frequently due to hyperbilirubinemia (primarily observed in treatment arms with ATV). Of the GI AEs, most were attributable to diarrhea or loose/watery stools. Many of these events occurred in subjects who were randomized to an arm containing BMS-955176 and ATV. Of the Grade 2-4 related AEs, many were due to headache and an increase in hyperbilirubinemia. Many of the AEs of hyperbilirubinemia occurred in subjects also receiving ATV; moreover, the three arms with the highest average total bilirubin occurred in subjects receiving both BMS-955176 and ATV. Clinical changes/symptoms consistent with the GI findings from dogs and rats (described above) were not seen in the preliminary data set from short-term therapy with BMS-955176 in HIV-1 infected adults.

In this treatment-naïve study population, we estimate GI safety multiples of 3×, 2×, and 1× (based on NOAEL in 9-month dog study), corresponding to projected human exposures at BMS-955176 doses of 60, 120, and 180 mg.

While no clinically relevant GI safety signals have been observed in AI468001 or AI468002, in this clinical trial, subjects will undergo routine targeted and complete history/physical exams in

Approved v3.0 930087119 3.0

addition to regular laboratory measurements (including CBC and chemistries). This will initially occur more frequently than in standard clinical practice and allow for increased vigilance for any potential GI toxicity. Guidance on the evaluation and management of potential GI toxicity is outlined in Section 6.7.1.3.

Subjects in this clinical study will benefit from receiving cART potentially containing BMS-955176. Preliminary data from the Phase 2a (Part A, B, and C) study show a maximum median reduction in HIV-1 RNA (clades B and C) ranging from 1.3 to 1.7 $\log_{10}$ in the dose arms estimated to provide similar exposure to those in this current study. When BMS-955716 was combined with ATV ± RTV (Part B) this resulted in maximum median declines in HIV-1 RNA ranging from 1.9 to 2.2 $\log_{10}$. These results are generally similar to the antiviral effect demonstrated by other classes of ARVs. Thus, BMS-955176 should contribute with other ARVs substantially to form an effective cART regimen.

As with any antiretroviral study in HIV-1-infected subjects, there is a risk for the development of treatment failure and the development of drug resistance associated mutations to BMS-955176 and/or other antiretrovirals. However, drug resistance to the maturation inhibitor would not be anticipated to result in cross-resistance to any other ARV class, including protease inhibitors.[14] Ongoing analysis of preliminary data from the Phase 2a study is evaluating both emergent genotypic and phenotypic changes after short term monotherapy with BMS-955176. The use of three fully susceptible agents as a part of cART is expected to decrease the probability of virologic failure and drug resistance. Initially in this clinical trial, measurement of HIV-1 RNA will occur more frequently than in standard clinical practice which will allow for increased vigilance for the development of lack of efficacy/resistance. Finally, an analysis for virologic futility will occur at Week 24 (see Section 8.4.7).

In-vitro studies show that BMS-955176 is expected to be active against a variety of HIV-1 clades albeit with EC50s approximately 2-3 fold less toward HIV-1 clade AE compared to clade B (see Section 1.4.1). As described in Section 1.1.1, the only clinical data for BMS-955176 exists in HIV-1 clade B and C. The efficacy profile of BMS-955176 in other HIV-1 clades encountered in other geographical regions where this multinational Phase 2b trial will be conducted (eg, clade AE in Thailand) is unknown. To mitigate the risk of randomizing subjects infected with HIV-1 with an unknown efficacy profile, this clinical trial will randomize a maximum of ~ 8% of total subjects (n = 16) who are infected with HIV-1 clade AE.

Of note, the other ARVs used in this clinical trial have a known and acceptable risk benefit ratio and are frequently prescribed to HIV-1 treatment-naïve infected adults as a part of standard of care.

Taken together, the clinical data to date show that BMS-955176 has potent antiretroviral activity and is generally safe and well tolerated in healthy volunteers and HIV-1 infected adults. These factors should allow subjects to benefit from achieving viral suppression whilst taking a generally safe and well-tolerated new antiretroviral with a novel mechanism of action. The risks including teratogenicity, GI toxicity, and drug resistance will be appropriately managed by following guidance in the study protocol.

Approved v3.0 930087119 3.0

2 ETHICAL CONSIDERATIONS

2.1 Good Clinical Practice

This study will be conducted in accordance with Good Clinical Practice (GCP), as defined by the International Conference on Harmonisation (ICH) and in accordance with the ethical principles underlying European Union Directive 2001/20/EC and the United States Code of Federal Regulations, Title 21, Part 50 (21CFR50).

The study will be conducted in compliance with the protocol. The protocol and any amendments and the subject informed consent will receive Institutional Review Board/Independent Ethics Committee (IRB/IEC) approval/favorable opinion prior to initiation of the study.

All potential serious breaches must be reported to BMS immediately. A serious breach is a breach of the conditions and principles of GCP in connection with the study or the protocol, which is likely to affect, to a significant degree, the safety or physical or mental integrity of the subjects of the study or the scientific value of the study.

Personnel involved in conducting this study will be qualified by education, training, and experience to perform their respective tasks.

This study will not use the services of study personnel where sanctions have been invoked or where there has been scientific misconduct or fraud (eg, loss of medical licensure, debarment).

2.2 Institutional Review Board/Independent Ethics Committee

Before study initiation, the investigator must have written and dated approval/favorable opinion from the IRB/IEC for the protocol, consent form, subject recruitment materials (eg, advertisements), and any other written information to be provided to subjects. The investigator or BMS should also provide the IRB/IEC with a copy of the Investigator Brochure or product labeling information to be provided to subjects and any updates.

The investigator or BMS should provide the IRB/IEC with reports, updates and other information (eg, expedited safety reports, amendments, and administrative letters) according to regulatory requirements or institution procedures.

2.3 Informed Consent

Investigators must ensure that subjects are clearly and fully informed about the purpose, potential risks, and other critical issues regarding clinical studies in which they volunteer to participate.

In situations where consent cannot be given to subjects, their legally acceptable representatives (as per country guidelines) are clearly and fully informed about the purpose, potential risks, and other critical issues regarding clinical studies in which the subject volunteers to participate.

BMS will provide the investigator with an appropriate (ie, Global or Local) sample informed consent form which will include all elements required by ICH, GCP and applicable regulatory requirements. The sample informed consent form will adhere to the ethical principles that have their origin in the Declaration of Helsinki.

Approved v3.0 930087119 3.0

Investigators must:

1) Provide a copy of the consent form and written information about the study in the language in which the subject is most proficient prior to clinical study participation. The language must be non-technical and easily understood
2) Allow time necessary for subject or subject's legally acceptable representative to inquire about the details of the study
3) Obtain an informed consent signed and personally dated by the subject or the subject's legally acceptable representative and by the person who conducted the informed consent discussion
4) Obtain the IRB/IEC's written approval/favorable opinion of the written informed consent form and any other information to be provided to the subjects, prior to the beginning of the study, and after any revisions are completed for new information
5) If informed consent is initially given by a subject's legally acceptable representative or legal guardian, and the subject subsequently becomes capable of making and communicating his or her informed consent during the study, consent must additionally be obtained from the subject
6) Revise the informed consent whenever important new information becomes available that is relevant to the subject's consent. The investigator, or a person designated by the investigator, should fully inform the subject or the subject's legally acceptable representative or legal guardian, of all pertinent aspects of the study and of any new information relevant to the subject's willingness to continue participation in the study. This communication should be documented

The confidentiality of records that could identify subjects must be protected, respecting the privacy and confidentiality rules applicable to regulatory requirements, the subjects' signed ICF and, in the US, the subjects' signed HIPAA Authorization.

The consent form must also include a statement that BMS and regulatory authorities have direct access to subject records.

Subjects unable to give their written consent (eg, stroke or subjects with or severe dementia) may only be enrolled in the study with the consent of a legally acceptable representative. The subject must also be informed about the nature of the study to the extent compatible with his or her understanding, and should this subject become capable, he or she should personally sign and date the consent form as soon as possible. The explicit wish of a subject who is unable to give his or her written consent, but who is capable of forming an opinion and assessing information to refuse participation in, or to be withdrawn from, the clinical study at any time should be considered by the investigator.

The rights, safety, and well-being of the study subjects are the most important considerations and should prevail over interests of science and society.

Approved v3.0 930087119 3.0

3 INVESTIGATIONAL PLAN

3.1 Study Design and Duration

This is a randomized, active-controlled, double-blind clinical trial. Approximately 200 treatment-naïve HIV-1 infected subjects will be randomized 1:1:1:1 to one of four treatment arms (approximately 50 per arm) with the resulting drugs/doses as follows:

- Arm 1: BMS-955176 60 mg QD + TDF/FTC 300/200 mg QD, or
- Arm 2: BMS-955176 120 mg QD + TDF/FTC 300/200 mg QD, or
- Arm 3: BMS-955176 180 mg QD + TDF/FTC 300/200 mg QD, or
- Arm 4: EFV 600 mg QD + TDF/FTC 300/200 mg QD

Randomization will be stratified by HIV-1 Clade (AE versus Other). The number of subjects with HIV-1 Clade AE will be capped at a maximum of approximately 16 total, approximately 4 per treatment arm.

3.1.1 Screening

The screening period begins with the subject's signature on the informed consent form (ICF).

The subject is then enrolled via the Interactive Voice Response System IVRS (or its web-based equivalent) See Section 4.4.

If the subject meets all eligibility criteria, the subject must be randomized within the 42 day screening period.

3.1.2 Day 1/Baseline Visit

On the Day 1 Visit, subjects will begin QD dosing with the blinded BMS-955176 or blinded EFV, each in combination with open-label TDF/FTC (see Section 4.5.1 for Instructions for Dose Administration).

The combinations assigned per arm of containers of BMS-955176 active dose/placebo and EFV active dose/placebo will achieve the designated dose of BMS-955176 60, 120, or 180 mg or EFV 600 mg so that treatment arms cannot be discerned:

- Subjects in Arm 1 will receive containers that have:
  - BMS-955176 60 mg active dose
  - BMS-955176 placebo matching 120 mg
  - EFV placebo matching 600 mg
  - open-label container of TDF/FTC 300/200 mg
- Subjects in Arm 2 will receive containers that have:
  - BMS-955176 placebo matching 60 mg
  - BMS-955176 120 mg active dose

Approved v3.0 930087119 3.0

    - EFV placebo matching 600 mg
    - open-label container TDF/FTC 300/200 mg
- Subjects in Arm 3 will receive containers that have:
    - BMS-955176 60 mg active dose
    - BMS-955176 120 mg active dose
    - EFV placebo matching 600 mg
    - open-label container TDF/FTC 300/200 mg
- Subjects in Arm 4 will receive containers that have:
    - BMS-955176 placebo matching 60 mg
    - BMS-955176 placebo matching 120 mg
    - EFV 600 mg active dose
    - open-label container TDF/FTC 300/200 mg

3.1.3 Optional Week 2 Intensive PK Visit

Subjects with anemia, defined as Hemoglobin < 11.0 g/dL, should be excluded from participation in the Week 2 Intensive PK Substudy.

Subjects in all arms will have the opportunity to participate in an elective Intensive PK Substudy visit at Week 2 (window for visit: Day 12-16). Approximately 48 subjects, 12 subjects from each arm, are expected to participate in the substudy; BMS may allow the substudy to over-enroll in an effort to have a sufficient number of complete datasets.

The series of 12 blood draws begins with pre-dose (0-hour) blood samples to be collected approximately 24 hours (20-28 hrs) after the morning doses of BMS-955176/placebo + TDF/FTC that were taken the day before. Ten more blood samplings are drawn through the 12-hr time point, with one final sampling collected at the 24-hr time point, requiring the subject to either remain overnight in the clinic, or to return the next morning; the final 24-hr sample will be collected prior to administration of the morning dose of BMS-955176/placebo + TDF/FTC (See Section 5.5.1).

PK Tools/Job Aids will be provided to assist with the proper sequencing of dosing and blood sample collections, as well as the collection of required data.

3.1.4 Visits Week 4 - 96

Subjects are expected to be treated for the duration of 96 weeks. After Day 1 and the optional Intensive PK visit at Week 2, subjects will be required to attend 12 more in-clinic study visits over the 96-week treatment period, as follows:

- Visits are conducted every 4 weeks from Week 4 through Week 16
- Visits are conducted every 8 weeks from Week 24 through Week 48
- Visits are conducted every 12 weeks from Week 60 through Week 96

Approved v3.0 930087119 3.0

Visits should be scheduled as an interval from the Day 1/Randomization date, and within a window of ± 5 days.

One of the visits should meet the very specific timing requirements as outlined in Section 5.5.2 for a pre-AM dose blood collection.

Telephonic visit will be conducted with each subject at visit Weeks 20, 28, 36, 44, 54, 66, 78, and 90 to conduct an adherence assessment and to continue retention efforts.

3.1.5 Selection of the Continuation Dose, and the Switch to open-label study medications

After the primary endpoint at Week 24 is reached (when all subjects have reached Week 24), BMS will conduct an analysis using the data through Week 24.

As described in Section 8.4.7, an analysis of Virologic Futility will also occur. If a BMS-955176 dose arm meets criteria for Virologic Futility at Week 24, subjects in that arm will begin dosing with the next highest available remaining dose of BMS-955176.

Based upon the Week 24 analysis, a recommended dose for continuation will be selected* and subjects in the BMS-955176 treatment arms will be unblinded and transitioned to the recommended, open-label Continuation Dose of BMS-955176; subjects in the EFV arm will be unblinded and transitioned to open-label EFV.

The open-label TDF/FTC backbone will remain unaltered.

> ** If the Continuation Dose cannot be clearly identified using the Week 24 data, the study will continue to be blinded to Investigator and subjects until an analysis of the Week 48 data can be performed and the Continuation Dose is selected.*

After the Continuation Dose is selected and once all of the logistics (eg, distribution of clinical drug supplies, activation of the new portion of the IVRS) have been completed globally, the transition of the subjects on the BMS-955176 arms to the open-label Continuation Dose, and of the subjects on the EFV arm to open-label EFV, will occur. It is anticipated that this transition will occur on or after all subjects have reached Week 48 (the earliest subjects to begin study treatment could be well beyond Week 48 when the switch to the open-label BMS-955176 or EFV occurs).

Investigators and subjects will be blinded to the treatment arm assignment (and to the dose of BMS-955176 for Arms 1-3) until after the switch to the Continuation Dose.

3.1.6 Post-dosing Follow-up Period

Subjects who discontinue the study early, or those who reach Week 96 and choose not to continue to receive BMS supplied study drug via the methods described in Section 3.2, are required to enter a post-dosing follow-up period to be seen for a safety visit 12 weeks after discontinuation.

Approved v3.0 930087119 3.0

3.1.7 End of the study

The end of the study will occur when the last study visit has been completed, defined as the final subject completing their final study visit (expected to be 12 weeks after a Week 96 or Early Termination visit for subjects required to complete the post-dosing follow-up period).

The study design schematic is presented in Figure 3.1.7-1.

Approved v3.0 930087119 3.0

Figure 3.1.7-1: Study Design Schematic

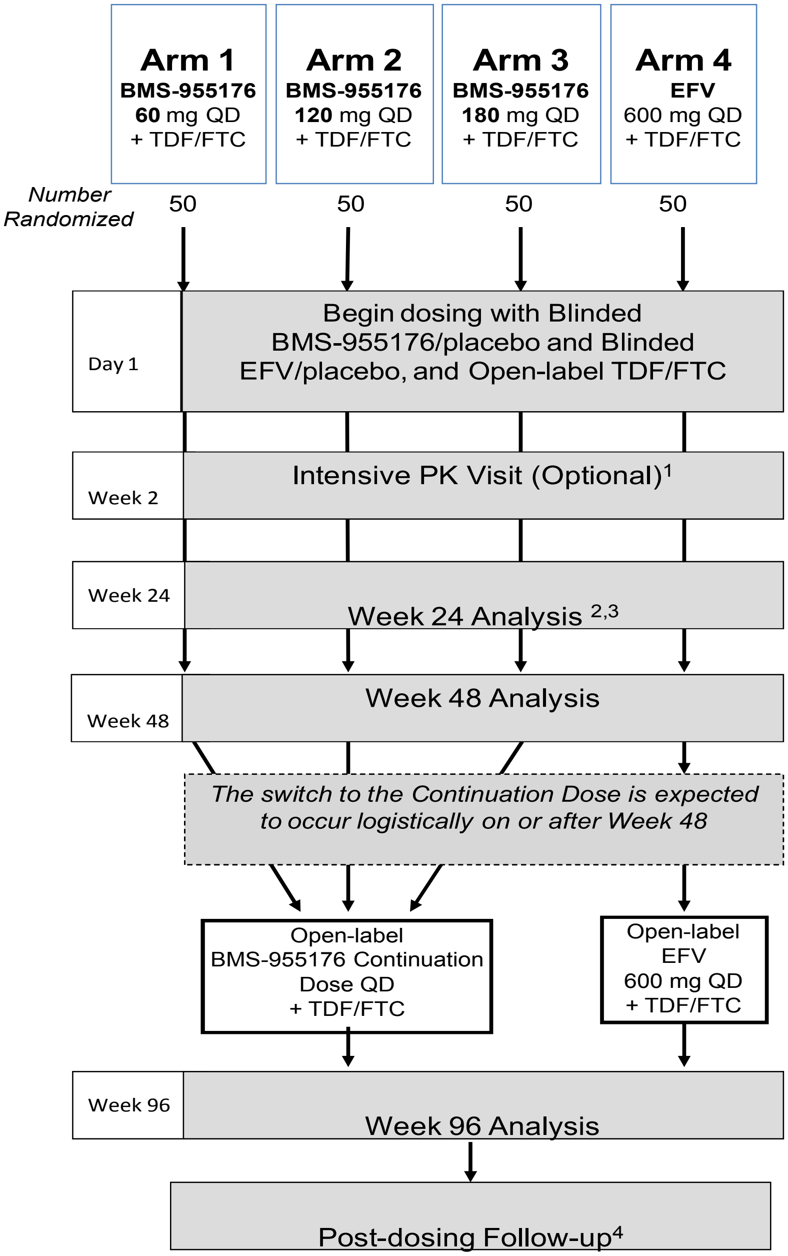

1 *Subjects with Hemoglobin < 11.0 g/dL should be excluded from participation in the optional Week 2 Intensive PK visit.*

2 *Virologic Futility will be evaluated (see Section 8.4.7)*

3 *The selection of the Continuation Dose will be made using the Week 24 data. If selection of the Continuation Dose cannot be made using the Week 24 data, the assessment will be made using Week 48 data.*

4 *Subjects who discontinue the study early, or who reach Week 96 but choose not continue with BMS supplied study drugs via the methods described in Section 3.2, are required to be seen for one visit 12 weeks after end of study treatments.*

Approved v3.0 930087119 3.0

3.2 Post Study Access to Therapy

At the conclusion of the study, subjects who continue to demonstrate clinical benefit will be eligible to receive BMS supplied study drug. Study drug will be provided via an extension of the study, a rollover study requiring approval by responsible health authority and ethics committee or through another mechanism at the discretion of BMS. BMS reserves the right to terminate access to BMS supplied study drug if any of the following occur: a) the marketing application is rejected by responsible health authority; b) the study is terminated due to safety concerns; c) the subject can obtain medication from a government sponsored or private health program; or d) therapeutic alternatives become available in the local market.

3.3 Study Population

For entry into the study, the following criteria MUST be met.

3.3.1 Inclusion Criteria

1. **Signed Written Informed Consent**
   a) Ability to understand and sign a written informed consent form
2. **Target Population**
   a) Antiretroviral treatment-naïve; defined as no current or previous exposure to > 1 week of an antiretroviral drug
   b) Plasma HIV-1 RNA ≥ 1000 copies/mL
   c) CD4 T-cell count > 200 cells/mm^3
   d) Fully susceptible to study drugs EFV, TDF, FTC as seen on Screening genotype/phenotype report(s)
   e) Estimated Life expectancy ≥ 1 year
   f) Subject Re-enrollment: This study permits the re-enrollment of a subject that has discontinued the study as a pre-treatment failure (ie, subject has not been randomized / has not been treated). If re-enrolled, the subject must be re-consented and assigned a new PID. Re-enrollment is limited to one occurrence.
3. **Age and Reproductive Status**
   a) Males and non-pregnant females
   b) At least 18 years of age, (or minimum age as determined by local regulatory or as legal requirements dictate)
   c) Willingness to use approved highly effective methods of contraception (see below) to avoid pregnancy (female subjects who are WOCBP and male subjects who are sexually active with WOCBP)
   d) Women of childbearing potential (WOCBP) must have a negative serum or urine pregnancy test (minimum sensitivity 25 IU/L or equivalent units of HCG) within 24 hours prior to the start of study drug

Approved v3.0 930087119 3.0

e) Women must not be breastfeeding

f) WOCBP must agree to follow instructions for method(s) of contraception for the duration of treatment with study drug BMS-955176 plus 12 weeks post-treatment completion

g) Males who are sexually active with WOCBP must agree to follow instructions for method(s) of contraception for the duration of treatment with study drug BMS-955176 plus 5 half-lives of the study drug BMS-955176 (8 days) plus 90 days (duration of sperm turnover) for a total of 98 days post-treatment completion

h) Azoospermic males and WOCBP who are continuously not heterosexually active are exempt from contraceptive requirements. However WOCBP must still undergo pregnancy testing as described in this section

Investigators shall counsel WOCBP and male subjects who are sexually active with WOCBP on the importance of pregnancy prevention and the implications of an unexpected pregnancy. Investigators shall advise WOCBP and male subjects who are sexually active with WOCBP on the use of highly effective methods of contraception.

All subjects must agree to the use of a highly effective method of contraception as listed below. In addition male subjects should use condoms during treatment and for 98 days following the last treatment when relevant systemic exposure is no longer present.

HIGHLY EFFECTIVE METHODS OF CONTRACEPTION

Highly effective methods of contraception have a failure rate of < 1% when used consistently and correctly. WOCBP and female partners of male subjects, who are WOCBP, are expected to use one of the highly effective methods of contraception listed below.

Study subjects who are WOCBP cannot use hormonal methods of contraception as one of the highly effective methods of contraception because there are data to show a lack of effectiveness of systemic hormonal contraceptives in women taking BMS-955176. However, WOCBP can continue to use hormonal contraceptives, if necessary, in addition to one other non-hormonal highly effective methods of contraception.

Male subjects must inform their female partners who are WOCBP of the contraceptive requirements of the protocol and are expected to adhere to using contraception with their partner. Relevant exposure of BMS-955176 in female partners of male participants in the study is expected to be negligible. Female partners of male subjects participating in the study may use hormone based contraceptives as one of the acceptable methods of contraception because exposure to the investigational product would be too small to alter exposure of hormonal contraceptives.

1. Nonhormonal IUDs, such as ParaGard®
2. Bilateral tubal occlusion
3. Vasectomised partner with documented azoospermia 90 days after procedure

Approved v3.0 930087119 3.0

- o Vasectomised partner is a highly effective birth control method provided that partner is the sole sexual partner of the WOCBP trial participant and that the vasectomised partner has received medical assessment of the surgical success.

4. Complete abstinence

- o Complete abstinence is defined as the complete avoidance of heterosexual intercourse when this is the preferred lifestyle of the patient.
- o Complete abstinence is an acceptable form of contraception for all study drugs and must be used throughout the duration of study and for the duration of time as specified above.
- o It is not necessary to use any other method of contraception when complete abstinence is elected.
- o Subjects who choose complete abstinence must continue to have pregnancy tests.
- o Acceptable alternate methods of highly effective contraception must be discussed in the event that the subject chooses to forego complete abstinence.

The reliability of sexual abstinence needs to be evaluated in relation to the duration of the clinical trial and the preferred and usual lifestyle of the subject.

At a minimum, subjects must agree to the use of two methods of contraception, with one method being highly effective and the other method being either highly effective or less effective as listed below:

LESS EFFECTIVE METHODS OF CONTRACEPTION

- Diaphragm with spermicide
- Cervical cap with spermicide
- Vaginal sponge with spermicide
- Male or female condom with or without spermicide*
- By male subject's WOCBP partner only: Progestogen-only oral hormonal contraception, where inhibition of ovulation is not the primary mode of action.

* A male and female condom must not be used together

3.3.2 Exclusion Criteria

1. **Target Disease Exceptions**

    a) Resistance or partial resistance to any study drug determined by tests at Screening

    b) Current or historical genotypic and/or phenotypic drug resistance testing showing resistance to EFV: K103N, L100I, K101E/P, V106A/M, Y181C/I/V, Y188L/C/H, G190A/S/E, or M230L

    c) Current or historical genotypic and/or phenotypic drug resistance testing showing resistance to TDF: K65R or T69ins

    d) Current or historical genotypic and/or phenotypic drug resistance testing showing resistance to FTC: M184I/V or Q151M

Approved v3.0 930087119 3.0

e) Current or historical genotypic drug resistance testing showing any of the following non-accessory thymidine analogue mutations (TAMs): M41L, D67N, K70R, L210W, T215Y/F, K219Q/E

f) Current or historical genotypic drug resistance testing showing primary protease inhibitor resistance mutations: I50L, I84V/A, N88D/S, I47A/V, V82A/F/T/S. These exclusionary mutations are listed only in order to allow subjects who develop on-treatment emergent virologic resistance a subsequent PI-based regimen (after study discontinuation)

2. Medical History and Concurrent Diseases

a) A new AIDS defining condition diagnosed within the 30 days prior to screening (see Appendix 2)

b) Any other clinical condition (including but not limited to active substance use) or prior therapy that, in the opinion of the Investigator, would make the subject unsuitable for the study; unable to comply with dosing requirements; or unable to comply with study visits; or a condition that could affect the absorption, distribution, metabolism or excretion of the drug

3. Physical and Laboratory Test Findings

a) Chronic HBV/HCV (Positive blood screen for HBsAg; Positive blood screen for HCV Ab and HCV RNA)

b) ALT or AST > 3 × ULN

c) Alkaline Phosphatase > 5 × ULN

d) Bilirubin ≥ 1.5 × ULN

e) History of decompensated cirrhosis or active decompensated cirrhosis

f) Hemoglobin < 8.0 g/dL

g) Platelets < 50,000 cells/mm^3

h) Estimated eGFR < 60 mL/min (CKD-EPI formula)

i) Confirmed QT value > 500 msec at Screening or Day 1

j) Confirmed QTcF value > 470 msec for women and > 450 msec for men at Screening or Day 1

k) Confirmed PR Interval > 260 msec (severe first degree AV block) at Screening or Day 1

l) Confirmed second or third degree heart block at Screening or Day 1

4. Allergies and Adverse Drug Reaction

a) Medications contraindicated for use with investigational/non-investigational study drugs (EFV, TDF, and FTC); or subjects with any known allergies to the investigational/non-investigational study drugs (EFV, TDF, or FTC)

b) Current or anticipated treatment with any of the medication listed in Appendix 1, in addition to any medications that are contraindicated with EFV, TDF or FTC.

c) Participation in an experimental drug and/or HIV-1 vaccine trial(s) within 30 days prior to Screening

Approved v 3.0 930087119 3.0

5. Other Exclusion Criteria

a) Prisoners or subjects who are involuntarily incarcerated
b) Subjects who are compulsorily detained for treatment of either a psychiatric or physical (eg, infectious disease) illness

Eligibility criteria for this study have been carefully considered to ensure the safety of the study subjects and that the results of the study can be used. It is imperative that subjects fully meet all eligibility criteria.

3.3.3 Women of Childbearing Potential

A woman of childbearing potential (WOCBP) is defined as any female who has experienced menarche and who has not undergone surgical sterilization (hysterectomy or bilateral oophorectomy) and is not postmenopausal. Menopause is defined as 12 months of amenorrhea in a woman over age 45 years in the absence of other biological or physiological causes. In addition, females under the age of 55 years must have a serum follicle stimulating hormone, (FSH) level > 40 mIU/mL to confirm menopause.

Females treated with hormone replacement therapy, (HRT) are likely to have artificially suppressed FSH levels and may require a washout period in order to obtain a physiologic FSH level. The duration of the washout period is a function of the type of HRT used. The duration of the washout period below are suggested guidelines and the investigators should use their judgement in checking serum FSH levels. If the serum FSH level is > 40 mIU/mL at any time during the washout period, the woman can be considered postmenopausal:

- 1 week minimum for vaginal hormonal products (rings, creams, gels)
- 4 week minimum for transdermal products
- 8 week minimum for oral products

Other parenteral products may require washout periods as long as 6 months.

3.4 Concomitant Treatments

3.4.1 Prohibited and/or Restricted Treatments

Refer to Appendix 1 which details prohibited and precautionary therapies during the study, including important information regarding hormonal methods of contraception.

3.4.2 Other Restrictions and Precautions

None.

Approved v3.0 930087119 3.0

3.5 Discontinuation of Subjects following any Treatment with Study Drug

Subjects MUST discontinue investigational product (and non-investigational product at the discretion of the investigator) for any of the following reasons:

- Subject's request to stop study treatment
- Any clinical adverse event (AE), laboratory abnormality or intercurrent illness which, in the opinion of the investigator, indicates that continued participation in the study is not in the best interest of the subject
- Termination of the study by Bristol-Myers Squibb (BMS)
- Loss of ability to freely provide consent through imprisonment or involuntarily incarceration for treatment of either a psychiatric or physical (eg, infectious disease) illness
- Pregnancy (subjects should be discontinued in an appropriate manner)
- Unblinding a subject for any reason (emergency or non-emergency)
- Repeat non-adherence by the subject with the requirements of the protocol or treatment (as determined by Investigator in consultation with the BMS Medical Monitor)
- Evidence of Hepatitis B or C infection
- Confirmed plasma HIV-1 RNA ≥ 1000 c/mL after Week 24
- Confirmed plasma HIV-1 RNA ≥ 200 c/mL after Week 48
- Emergence of genotypic and/or phenotypic resistance to any component of the study treatment regimen at any time after Screening
- Subject requires switching to any other ARV
- Development of pDILI (potential drug induced liver injury)
- Confirmed QTcB or QTcF value > 500 msec
- Confirmed second degree (Type II) or third degree AV block at any time during the study
- New onset of serious mental illness or suicidal ideation that in the opinion of the investigators makes continuation in the study not in the best interest of the subject

All subjects who discontinue study drug should comply with protocol specified follow-up procedures as outlined in Section 5 (ie, perform an Early Termination [ET] visit).The only exception to this requirement is when a subject withdraws consent for all study procedures including post-treatment study follow-up (no such period exists in this study) or loses the ability to consent freely (ie, is imprisoned or involuntarily incarcerated for the treatment of either a psychiatric or physical illness).

If study drug is discontinued prior to the subject's completion of the study, the reason for the discontinuation must be documented in the subject's medical records and entered on the appropriate case report form (CRF) page.

3.6 Post Study Drug Study Follow up

Subjects who discontinue study drug may continue to be followed.

Approved v3.0 930087119 3.0

Subject's contact information will be collected/confirmed throughout the study so that subjects who discontinue study drug may continue to be followed for resolution of a pregnancy or SAE.

Subjects who discontinue the study early, or who reach Week 96 but choose not continue with BMS supplied study drugs via the methods described in Section 3.2, are required to be seen for one visit 12 weeks after end of study treatments.

3.6.1 Withdrawal of Consent

Subjects who request to discontinue study drug will remain in the study and must continue to be followed for protocol specified follow-up procedures. The only exception to this is when a subject specifically withdraws consent for any further contact with him/her or persons previously authorized by subject to provide this information. Subjects should notify the investigator of the decision to withdraw consent from future follow-up **in writing**, whenever possible. The withdrawal of consent should be explained in detail in the medical records by the investigator, as to whether the withdrawal is from further treatment with study drug only or also from study procedures and/or post treatment study follow-up, and entered on the appropriate CRF page. In the event that vital status (whether the subject is alive or dead) is being measured, publicly available information should be used to determine vital status only as appropriately directed in accordance with local law.

3.6.2 Lost to Follow-Up

All reasonable efforts must be made to locate subjects to determine and report their ongoing status. This includes follow-up with persons authorized by the subject as noted above. Lost to follow-up is defined by the inability to reach the subject after a minimum of three documented phone calls, faxes, or emails as well as lack of response by subject to one registered mail letter. All attempts should be documented in the subject's medical records. If it is determined that the subject has died, the site will use permissible local methods to obtain the date and cause of death.

If investigator's use of third-party representative to assist in the follow-up portion of the study has been included in the subject's informed consent, then the investigator may use a Sponsor-retained third-party representative to assist site staff with obtaining subject's contact information or other public vital status data necessary to complete the follow-up portion of the study. The site staff and representative will consult publicly available sources, such as public health registries and databases, in order to obtain updated contact information. If after all attempts, the subject remains lost to follow-up, then the last known alive date as determined by the investigator should be reported and documented in the subject's medical records.

Approved v3.0 930087119 3.0

4 STUDY DRUG

Study drug includes both Investigational [Medicinal] Product (IP/IMP) and Non-investigational [Medicinal] Product (Non-IP/Non-IMP) and can consist of the following:

Table 4-1: Study Drugs for AI468038:

Product Description / Class and Dosage Form	Potency	IMP / Non-IMP	Blinded or Open Label	Packaging/ Appearance	Storage Conditions (per label)
BMS-955176	60 mg[a]	IMP	Blinded	Bottle/ A white to off-white, biconvex, oval shaped film coated tablet	Store at 15 - 25°C Protect from light. Store in a tightly closed container
BMS-955176	120 mg[a]	IMP	Blinded	Bottle/ A white to off-white, biconvex, capsule shaped film coated tablet	Store at 15 - 25°C Protect from light. Store in a tightly closed container
Placebo to match BMS-955176 60 mg[a]	N/A	IMP	Blinded	Bottle/ A white to off-white, biconvex, oval shaped film coated tablet	Store at 15 - 25°C Protect from light. Store in a tightly closed container
Placebo to match BMS-955176 120 mg[a]	N/A	IMP	Blinded	Bottle/ A white to off-white, biconvex, capsule shaped film coated tablet	Store at 15 - 25°C Protect from light. Store in a tightly closed container
BMS-955176	60 mg[a]	IMP	Open Label	Bottle/ A white to off-white, biconvex, oval shaped film coated tablet	Store at 2 - 30°C Protect from light. Store in a tightly closed container
BMS-955176	120 mg[a]	IMP	Open Label	Bottle/ A white to off-white, biconvex, capsule shaped film coated tablet	Store at 2 - 30°C Protect from light. Store in a tightly closed container

Approved v3.0 930087119 3.0

Table 4-1: Study Drugs for AI468038:

Product Description / Class and Dosage Form	Potency	IMP / Non-IMP	Blinded or Open Label	Packaging/ Appearance	Storage Conditions (per label)
Efavirenz (EFV)	600 mg	IMP	Blinded	Bottle/<br>A yellow, modified capsule shaped, film coated tablet	Store at 15 - 25°C
Placebo to match Efavirenz (EFV) 600 mg	N/A	IMP	Blinded	Bottle/<br>A yellow, modified capsule shaped, film coated tablet	Store at 15 - 25°C
Efavirenz (EFV)	600 mg	IMP	Open Label	Bottle/<br>A yellow, modified capsule shaped, film coated tablet	Store at 15 - 25°C
TDF/FTC[b]	300 mg/200 mg	Non-IMP	Open-Label	Various packaging configurations	Refer to label on container or package insert

[a] 180 mg of BMS-955176 active/placebo will be constructed with BMS-955176 60 mg/placebo + BMS-955176 120 mg/placebo

[b] Some regions may be supplied with TD/FTC 245/200 mg; tenofovir disoproxil (TD) 245 mg is equivalent to tenofovir disoproxil fumarate (TDF) 300 mg.[13]

Approved v3.0 930087119 3.0

4.1 Investigational Product

An investigational product, also known as investigational medicinal product in some regions, is defined a pharmaceutical form of an active substance or placebo being tested or used as a reference in a clinical study, including products already with a marketing authorization but used or assembled (formulated or packaged) differently than the authorized form, or used for an unauthorized indication, or when used to gain further information about the authorized form.

The investigational product should be stored in a secure area according to local regulations. It is the responsibility of the investigator to ensure that investigational product is only dispensed to study subjects. The investigational product must be dispensed only from official study sites by authorized personnel according to local regulations.

In this protocol, investigational product(s) is/are: BMS-955176 and EFV. These products will be supplied.

4.2 Non-investigational Product

Other medications used as support or escape medication for preventative, diagnostic, or therapeutic reasons, as components of the standard of care for a given diagnosis, may be considered as non-investigational products.

In this protocol, noninvestigational product(s) is/are: TDF/FTC. This product will be supplied.

4.3 Storage and Dispensing

The product storage manager should ensure that the study drug is stored in accordance with the environmental conditions (temperature, light, and humidity) as determined by BMS. If concerns regarding the quality or appearance of the study drug arise, the study drug should not be dispensed and contact BMS immediately.

Study drug not supplied by BMS will be stored in accordance with the package insert.

Investigational product documentation (whether supplied by BMS or not) must be maintained that includes all processes required to ensure drug is accurately administered. This includes documentation of drug storage, administration and, as applicable, storage temperatures, reconstitution, and use of required processes (eg, required diluents, administration sets).

Storage facilities for controlled substances must be securely locked and substantially constructed, with restricted access to prevent theft or diversion, as applicable by local regulations.

4.4 Method of Assigning Subject Identification

At the start of the screening period, the investigative staff will call the Assignment Center via an Interactive Voice Response System ([IVRS], or its web-based equivalent) designated by the sponsor to enroll the subject and to obtain a subject patient identification number (PID).

For subjects who meet the protocol eligibility criteria, the investigative staff will call the IVRS and subjects will start treatment. Subjects will be randomly assigned 1:1:1:1 to one of

Approved v 3.0 930087119 3.0

4 treatment arms, and stratified by HIV-1 Clade (AE versus Other), as described in Section 3 and as outlined in the AI468038 Study Schematic Figure 3.1.7-1.

Note: All efforts should be made to limit the possibility of randomizing subjects that do not start treatment. If a subject is randomized but does not receive study medication, the BMS study team must be notified immediately.

4.5 Selection and Timing of Dose for Each Subject

Subjects will be randomly assigned 1:1:1:1 to one of four treatment arms as described in Section 3.1, and will begin dosing in a blinded fashion on Day 1 so that they receive the following active doses:

Arm 1: BMS-955176 60 mg QD + TDF/FTC 300/200 mg QD, or

Arm 2: BMS-955176 120 mg QD + TDF/FTC 300/200 mg QD, or

Arm 3: BMS-955176 180 mg QD + TDF/FTC 300/200 mg QD, or

Arm 4: EFV 600 mg QD + TDF/FTC 300/200 mg QD

4.5.1 *Instructions for Dose Administration*

4.5.1.1 *General Instructions*

- Subjects should administer doses of each drug from only one bottle at a time, until that bottle is empty, before another bottle may be opened
- Subjects will be required to complete Dosing Diaries so that drug administration can be accurately accounted. It is important that sites provide instructions to subjects for completion and obtain their acknowledgment that doing so provides critical information for this dose-finding clinical trial
- Dosing times (and study appointment times) must be carefully considered through Week 24 due to the requirements of the PK collection outlined in Section 5.5

4.5.1.2 *Instructions for ALL SUBJECTS for dosing during the blinded portion of the study*

- Morning Dose: In the morning, with a meal, subjects will take one pill from each of the following bottles, which will result in the administration of the total drugs/doses required for the assigned Arms 1-4:
    - Bottle **A** *BMS-955176 60 mg or placebo*
    - Bottle **B** *BMS-955176 120 mg or placebo*
    - the open-label bottle *emtricitabine/tenofovir*
- Evening Dose: At bed time, on an empty stomach, without food, subjects will take one pill from:
    - Bottle C *EFV 600 mg or placebo*

Approved v3.0 930087119 3.0

4.5.1.3 Instructions for dosing during the fully open-label portion of the study

After the BMS-955176 Continuation Dose is selected, all subjects will be switched to a fully open-label regimen.

Subjects in the BMS-955176 arms:

- Morning Dose: In the morning, with a meal, subjects will take one pill daily from each of the following:
  - open-label bottle(s) *BMS-955176 60 mg* or *BMS-955176 120 mg, or both* (depending on the selected Continuation Dose)
  - open-label bottle *emtricitabine/tenofovir*
- Evening Dose: There is no longer an evening dose for subjects on the BMS-955176 arms (subjects in the BMS-955176 arms were taking the EFV placebo at the evening dose during the blinded portion of the study)

Subjects in the EFV arm:

- Subjects will take one pill daily from the open-label bottle labeled *emtricitabine/tenofovir* in a manner consistent with local package insert and as instructed by the Investigator
- Evening Dose: At bed time, on an empty stomach, without food, subjects will take one pill from the open-label bottle labeled *EFV 600 mg*

4.5.2 Dose Modifications

No dose adjustments or changes in intake frequency are allowed for any of the assigned study drugs in the protocol, except in the event of treatment-limiting renal toxicity, dose interval adjustments for TDF/FTC are permitted according to the local package insert/label, and only after the completion of the Week 2 Intensive PK optional Visit, if the subject is inclined to participate.

4.6 Blinding/Unblinding

Blinding of treatment assignment is critical to the integrity of this clinical study. However, in the event of a medical emergency or pregnancy in an individual subject in which knowledge of the investigational product is critical to the subject's management, the blind for that subject may be broken by the investigator. The subject’s safety takes priority over any other considerations in determining if a treatment assignment should be unblinded.

Before breaking the blind of an individual subject's treatment, the investigator should determine that the unblinded information is necessary, ie, that it will alter the subject's immediate management. In many cases, particularly when the emergency is clearly not related to the investigational product, the problem may be properly managed by assuming that the subject is

Approved v3.0 930087119 3.0

receiving active product. It is highly desirable that the decision to unblind treatment assignment be discussed with the Medical Monitor, but the investigator always has ultimate authority for the decision to unblind. The Principal Investigator should only call in for emergency unblinding AFTER the decision to discontinue the subject has been made.

For this study, the method of unblinding for emergency purposes is IVRS.

For information on how to unblind in case of an emergency, consult the IVRS manual.

In cases of accidental unblinding, contact the Medical Monitor and ensure every attempt is made to preserve the blind.

Any request to unblind a subject for non-emergency purposes should be discussed with the Medical Monitor.

For analysis of pharmacokinetic samples, the Bioanalytical Sciences Group at BMS, and/or the designated vendor performing the analysis will be unblinded to the randomized treatment assignments in order to determine the appropriate analyses for each PK sample.

Access to the unblinded data will be restricted to a limited number of BMS and vendor personnel involved in the development of models for population pharmacokinetics, exposure-response relationships, and viral kinetics. This will include one or more pharmacokineticists, pharmacometricians, statisticians, and data programmers. Analysis results will not be revealed to the study team until after the interim data base lock at Week 24. Provisions will be taken to ensure the security of the data and that no other individuals should be unblinded.

4.7 Treatment Compliance

Treatment Adherence to the treatment regimen will be critical to the conduct of this study. Adherence will be evaluated by the investigative staff at every treatment visit (including telephone contact visits) through interviews with the subjects and through examination of returned medication. It is expected that site staff attempt to have subjects maintain 90% treatment compliance or greater. Subjects should be instructed to bring all unused study medication back in the original container to each visit. Site staff are required to review Dosing Diaries completed by the subject, and to reinforce their use.

4.8 Destruction of Study Drug

For this study, study drugs (those supplied by BMS or sourced by the investigator) such as partially used study drug containers, vials and syringes may be destroyed on site.

Any unused study drugs can only be destroyed after being inspected and reconciled by the responsible Study Monitor unless study drug containers must be immediately destroyed as required for safety, or to meet local regulations (eg, cytotoxics or biologics).

On-site destruction is allowed provided the following minimal standards are met:

- On-site disposal practices must not expose humans to risks from the drug

Approved v3.0 930087119 3.0

- On-site disposal practices and procedures are in agreement with applicable laws and regulations, including any special requirements for controlled or hazardous substances
- Written procedures for on-site disposal are available and followed. The procedures must be filed with the site's SOPs and a copy provided to BMS upon request
- Records are maintained that allow for traceability of each container, including the date disposed of, quantity disposed, and identification of the person disposing the containers. The method of disposal, ie, incinerator, licensed sanitary landfill, or licensed waste disposal vendor must be documented
- Accountability and disposal records are complete, up-to-date, and available for the Monitor to review throughout the clinical trial period

If conditions for destruction cannot be met the responsible Study Monitor will make arrangements for return of study drug.

It is the investigator's responsibility to arrange for disposal of all empty containers, provided that procedures for proper disposal have been established according to applicable federal, state, local, and institutional guidelines and procedures, and provided that appropriate records of disposal are kept.

4.9 Return of Study Drug

If study drug will not be destroyed upon completion or termination of the study, all unused and/or partially used study drug that was supplied by BMS must be returned to BMS. The return of study drug will be arranged by the responsible Study Monitor.

It is the investigator's responsibility to arrange for disposal of all empty containers, provided that procedures for proper disposal have been established according to applicable federal, state, local, and institutional guidelines and procedures, and provided that appropriate records of disposal are kept.

Approved v3.0 930087119 3.0

5 STUDY ASSESSMENTS AND PROCEDURES

5.1 Flow Chart/Time and Events Schedule

Table 5.1-1: Screening Procedural Outline (AI468038)

Procedure	Screening Visit (42-day screening period)	Notes
**Eligibility Assessments**		
Informed Consent	X	
Call IVRS to Enroll the subject; PID assigned	X	
Inclusion/Exclusion Criteria	X	
Medical History	X	
AIDS History	X	
**Safety Assessments**		
Full Physical Examination	X	See Section 5.3.1 for requirements
Vital Signs & Physical Measurements	X	See Section 5.3.1 for requirements
Pre-treatment events	X	CDC Class C events with onset during the Screening period
Serious Adverse Events Assessment	X	All SAEs that occur after the ICF has been signed should be reported
ECG	X	See Section 5.3.4 for requirements
Pregnancy Test	X	WOCBP<br>For females under age 55, an FSH level must be on record to confirm she is not a WOCBP if pregnancy testing is not being performed.<br>If positive urine, request serum hCG quant. on lab requisition.
**Laboratory Assessments (see Appendix 5 for full details)**		
Fasting Chemistry and Lipid Panel	X	Fasting overnight

Approved V3.0 930087119 3.0

**Table 5.1-1: Screening Procedural Outline (AI468038)**		
**Procedure**	**Screening Visit (42-day screening period)**	**Notes**
Hematology	X	
Urinalysis	X	
Plasma HIV-1 RNA	X	
CD4+ and CD8+ T-cell count	X	
HBV Surface Antigen	X	
HCV Serology	X	Positive HCV Ab will reflex to HCV RNA
Urine toxicology (drugs of abuse)	X	Could aid in the selection of appropriate study candidates.
Pharmacodiagnostic (PDx) sample[a]	X	Plasma collection to be banked for potential future use in development of novel predictive assay(s)
Resistance Testing (HIV-1 Drug Resistance)		
GenoSure	X	Complete set of test results may take up to 4 weeks.
PhenoSense Gag	X	
Next Generation Seq. - Qs Gag	X	
Exploratory Resistance (HIV-1 Drug Resistance)	X	Molecular analysis at BMS WFD Discovery of baseline resistant samples and baseline sensitive controls in cases of subsequent on-treatment virologic failure

[a] By definition, a pharmacodiagnostic sample (PDx) is a pre-treatment test to determine whether or not a patient is likely to respond to a drug (ie, a predictive test). Based on the results of clinical studies with BMS-955176, BMS may have to develop a PDx assay. Thus, PDx samples obtained at Screening in this study would be used for that sole purpose.

Approved v3.0 930087119 3.0

Table 5.1-2: Short-term Procedural Outline (AI468038)

Procedure	In-Clinic Visit<br>Day 1	In-Clinic Visit<br>Optional Week 2 for Intensive PK<br>(Day 12-16)	In-Clinic Visits<br>Weeks 4, 8, 12, 16, 24, 32, 40, 48, 60, 72, 84, 96, and/or Early Termination (ET)<br>(± 5 days)	Phone Visits<br>Weeks 20, 28, 36, 44, 54, 66, 78, 90<br>(± 5 days)	Post-dosing Follow-up Visit (12 weeks after treatment discontinuation, at Week 96 or Early Term)	Notes
**Eligibility Assessments**						
Inclusion/Exclusion Criteria	X					
**Non-Laboratory Safety Assessments**						
Full Physical Examination			WK 12, 24, 48, 96/ET			See Section 5.3.1 for requirements
Targeted Physical Examination	X		WK 4, 8, 16, 32, 40, 60, 72, 84		X	See Section 5.3.1 for requirements
Vital Signs & Physical Measurements	X		X		X	See Section 5.3.1 for requirements
Adherence Assessment			X	X		Including review of Dosing Diaries
Pre-treatment Events	X					See Table 5.1-1
Adverse Events	X	X	X		X<br>(SAE only)	Serious and Non-serious AEs
Concomitant Medications	X	X	X			See Section 5.3.3
ECG	X		WK 4, 12, 24, 48, 96/ET			See Section 5.3.4 for requirements
Pregnancy Test	X	X	X		X	For females under age 55, an FSH level must be on record to confirm she is not a WOCBP if

Approved v3.0 930087119 3.0

Table 5.1-2: Short-term Procedural Outline (AI468038)

Procedure	In-Clinic Visit<br>Day 1	In-Clinic Visit<br>Optional Week 2 for Intensive PK<br>(Day 12-16)	In-Clinic Visits<br>Weeks 4, 8, 12, 16, 24, 32, 40, 48, 60, 72, 84, 96, and/or Early Termination (ET)<br>(± 5 days)	Phone Visits<br>Weeks 20, 28, 36, 44, 54, 66, 78, 90<br>(± 5 days)	Post-dosing Follow-up Visit (12 weeks after treatment discontinuation, at Week 96 or Early Term)	Notes
						pregnancy testing is not being performed<br>If positive urine, request serum hCG quant. on lab requisition.
Provide WOCBP with Home Pregnancy test kit(s) to be used during the in-clinic visit interval			WK 16, 24, 32, 40, 48, 60, 72, 84			Provide One Kit at Weeks 16 - 40 Provide Two Kits at Weeks 48 - 84<br>WOCBP subjects perform test Q4 weeks at home and report results to site.
**Laboratory Assessments for Safety and Efficacy and Other Endpoints (See Appendix 5 for full details)**						
Fasting Chemistry	X		X		X	Fasting overnight
Fasting Lipid Panel	X		WK 4, 12, 24, 48, 96/ET			Fasting overnight
Hematology	X		X		X	
Urinalysis	X		X		X	
Plasma HIV-1 RNA	X	X	X			If collecting an HIV-1 RNA at an UNSCHEDULED visit, also collect samples for Resistance and Exploratory Resistance Testing

Approved v3.0 930087119 3.0

Table 5.1-2: Short-term Procedural Outline (AI468038)

Procedure	In-Clinic Visit<br>Day 1	In-Clinic Visit<br>Optional Week 2 for Intensive PK<br>(Day 12-16)	In-Clinic Visits<br>Weeks 4, 8, 12, 16, 24, 32, 40, 48, 60, 72, 84, 96, and/or Early Termination (ET)<br>(± 5 days)	Phone Visits<br>Weeks 20, 28, 36, 44, 54, 66, 78, 90<br>(± 5 days)	Post-dosing Follow-up Visit (12 weeks after treatment discontinuation, at Week 96 or Early Term)	Notes
CD4 and CD8 T-cell count	X		X			
HBV Surface Antigen			WK 48 and 96/ET			
HCV Serology			WK 48 and 96/ET			
Resistance Testing (HIV-1 Drug Resistance)						
PhenoSense GT	X		X			Samples stored and tested if needed (ie, analyses of subjects if deemed clinically relevant)<br>See Section 5.4.2.2
PhenoSense Gag	X		X			
Next Generation Seq. - Qs Gag	X		X			
Exploratory Resistance (HIV-1 Drug Resistance)	X		X			Samples stored and tested retrospectively if needed (ie, exploratory analyses for subjects if deemed clinically relevant)
Intensive PK sample collection		X				Subset of 12 subjects in each arm<br>Use of PK Tool/Job Aid for data collection recommended<br>See Section 5.5.1 for requirements
Sparse PK sample collection			WK 4, 8, 12, 16, 24			See Section 5.5.2 for

Approved v3.0 930087119 3.0

Table 5.1-2: Short-term Procedural Outline (AI468038)

Procedure	In-Clinic Visit<br>Day 1	In-Clinic Visit<br>Optional Week 2 for Intensive PK<br>(Day 12-16)	In-Clinic Visits<br>Weeks 4, 8, 12, 16, 24, 32, 40, 48, 60, 72, 84, 96, and/or Early Termination (ET)<br>(± 5 days)	Phone Visits<br>Weeks 20, 28, 36, 44, 54, 66, 78, 90<br>(± 5 days)	Post-dosing Follow-up Visit (12 weeks after treatment discontinuation, at Week 96 or Early Term)	Notes
						requirements
Backup Serum and Plasma Sample	X		X			Samples stored and tested if needed
**Clinical Drug Supply**						
Call IVRS to Randomize	X					
Dispense Study Drug	X		X			There is no dispensation at Week 96 or ET.

Approved v3.0 930087119 3.0

5.1.1 Retesting During Screening or Lead-in Period

Retesting of laboratory parameters and/or other assessments within any single Screening or Lead-in period will be permitted (in addition to any parameters that require a confirmatory value). The Screening Period for this study is 42 days.

Any new result will override the previous result (ie, the most current result prior to Randomization) and is the value by which study inclusion will be assessed, as it represents the subject's most current, clinical state.

Laboratory parameters and/or assessments that are included in Table 5.1-1, Screening Procedural Outline may be repeated in an effort to find all possible well-qualified subjects. Consultations with the Medical Monitor may be needed to identify whether repeat testing of any particular parameter is clinically relevant (eg, a previously failed inclusion criterion).

Rescreening is different than Retesting. Rescreening is the process of Re-enrollment and requires that all procedures be repeated in an entirely new screening period. Rescreening will also be allowed. If it is clinically reasonable, and upon Investigator assessment (and in consultation with the BMS Medical Monitor, if necessary), a subject may be rescreened multiple times during the entire course of the enrollment period. Therefore, subjects may be Rescreened once, if, in the opinion of the Investigator, a current clinical outlook of the subjects seems favorable for study inclusion (and if the patient never received study medication in this trial).

The assigned PID for the subject must be Screen Failed in the IVRS. A new call must be made to the IVRS for the assignment of a new PID for the subject, and all Screening parameters must be done again in reference to the new PID (See Section 3.3.1, Inclusion Criteria 2f). Subject must also be re-consented with the new PID.

5.2 Study Materials

The sponsor will provide each investigative site with the following:

- BMS-955176 Investigator Brochure (IB) and any relevant safety addenda or updates
- Protocol and any Amendments to the Protocol
- Instructions for completing electronic Case Report Forms (eCRFs)
- Laboratory Manual from the central laboratory
- ECG machines and manual
- IVRS Worksheets to complete when calling the IVRS center to enroll, randomize, and discontinue subjects
- PK Tools/Job Aids that may be used for detailed instruction about the PK visits, and as a comprehensive source for documents of date/times of dosing and blood sampling

Approved v3.0 930087119 3.0

- Dosing Diaries
    - Completion by subjects is required
    - Should include daily dose of study medications administered by subject, modified or missed
    - Site staff should review the diaries with the subject at each visit, and, in combination with detailed questioning, should be able to provide comprehensive information in the case report form, noting discrepancies in the subject's file. Dosing Diaries should be maintained in the subject's study file.

5.3 Safety Assessments

The investigative team should follow the protocol-specified scheduled of safety-related measurements. Only data for the procedures and assessments specified should be submitted to BMS on the case report form. Additional procedures and assessments may be performed as part of standard of care, however, data for these assessments should remain in the subject's medical record and should not be submitted to BMS, unless specifically requested (ie, as part of an SAE).

5.3.1 Vital Signs and Physical Examinations

The schedule of vital signs, physical examinations, and targeted physical examinations is provided in Section 5.1 (Flow Chart/Time and Events Schedule). Vital signs include heart rate, blood pressure, respiration rate, and temperature and should be measured after the subject has been sitting/resting for at least 5 minutes. Physical measurements include height and weight. Targeted physical examinations will include examination of the heart, lungs, skin, abdomen, any symptomatic organ system, and general appearance.

5.3.2 Adverse Events

Subjects will be closely monitored throughout the study for any new or ongoing HIV-related diagnoses (Appendix 2) and/or adverse events. CDC Class C events that occur from the Screening Visit through Day 1 (prior to dosing), will be recorded as Pre-treatment Events. All events that occur after dosing on Day 1 will be recorded on the appropriate Adverse Event eCRF. Additional information on Adverse Events is provided in Section 6.

5.3.3 Concomitant Medication Assessment

All medications taken from the Screening Visit throughout the duration of the study will be reported. In addition, any prior therapy with antiretroviral drugs (if applicable) will be reported (See Appendix 1 for Prohibited and Precautionary Therapies).

5.3.4 Electrocardiograms

The schedule of electrocardiograms (ECGs) is provided in Section 5.1 (Flow Chart/Time and Events Schedule). ECG machines will be provided by a central vendor who will also perform the read/interpretation of the output.

Approved v3.0 930087119 3.0

5.4 Efficacy Assessments

5.4.1 Primary Efficacy Assessment

The primary assessment for efficacy is HIV-1 RNA through Week 24.

5.4.1.1 Guidelines for Confirmatory Testing of Plasma HIV-1 RNA and Resistance testing

A confirmatory HIV-1 RNA viral load should be obtained when:

- HIV-1 RNA ≥ 40 c/mL if prior suppression < 40 c/mL or
- > 1 $\log_{10}$ c/mL increase in HIV-1 RNA at anytime above nadir level where nadir is ≥ 40 c/mL

All efforts should be made to collect this sample within 2-4 weeks from the collection of the original sample.

When collecting a blood sample for HIV-1 RNA testing at an Unscheduled visit, samples should also be collected for the sets of Resistance and Exploratory Resistance Tests, so that the samples are available should resistance testing be required or deemed necessary based on the result of the HIV-1 RNA test.

Table 5.4.1.1-1 describes the management of detectable HIV RNA-1

Table 5.4.1.1-1: Management of Detectable HIV-1 RNA, based on Confirmed (2-4 weeks from original sample) or Consecutive HIV-1 RNA Result[a]

**Day 1 through Week 24**	
40 - 399 c/mL	Reinforce Adherence
≥ 400 c/mL	Resistance testing should occur for all subjects with confirmed or consecutive HIV-1 RNA ≥ 400 copies/mL from Week 4 through Week 96.<br>Consider possible discontinuation of subject, in consultation with BMS Medical Monitor, and/or reinforce adherence.
**After Week 24 through Week 48**	
40 - 399 c/mL	Reinforce Adherence
400 - 999 c/mL	Resistance testing will be performed.<br>If resistance has developed, subject must be discontinued.<br>If resistance has not developed, consider possible discontinuation of subject, in consultation with BMS Medical Monitor, and/or reinforce adherence.

Approved v3.0 930087119 3.0

Table 5.4.1.1-1:	Management of Detectable HIV-1 RNA, based on Confirmed (2-4 weeks from original sample) or Consecutive HIV-1 RNA Result[a]
≥ 1000 c/mL	Resistance testing will be performed.<br>Regardless of result of resistance tests, subject must be discontinued (see Section 3.5).
**After Week 48**	
< 200 c/mL	Reinforce Adherence
≥ 200 c/mL	Subject must be discontinued (see Section 3.5).<br>Resistance testing should occur for all subjects with confirmed or consecutive HIV-1 RNA ≥ 400 copies/mL from Week 4 through Week 96.

[a] When discontinuation is required or otherwise warranted and resistance results are needed, subject may continue on study medication/on study until resistance testing results are available.

5.4.1.2 Protocol Defined Virologic Failure

Protocol Defined Virologic Failure (PDVF) is defined by a subject meeting one of the following criteria:

1) Confirmed ≥ 40 copies/mL if prior suppression to < 40 copies/mL or > 1 $\log_{10}$ c/mL increase in HIV-1 RNA at anytime above nadir

In addition to the clinical management outlined in section 5.4.1.1, samples meeting criteria for PDVF will also be sent for resistance and exploratory resistance testing.

5.4.2 Secondary Efficacy Assessments

5.4.2.1 CD4+ and CD8+ T-Cells

CD4+ and CD8+ T-cells counts and percentages will be assessed using flow cytometry. The schedule of assessments is provided in Section 5.1 (Flow Chart/Time and Events Schedule). Procedures for samples collection and processing are provided in the central clinical laboratory manual.

5.4.2.2 Drug Resistance Testing

Plasma samples for viral drug resistance testing will be collected at Screening for all subjects and the HIV-1 drug resistance genotype will be analyzed to rule out resistance to any component of the study regimen or specific resistance mutations as outlined in Section 3.3.2, Exclusionary Criteria. At subsequent visits, samples for emergent drug resistance testing (both genotypic and phenotypic) will be collected and stored to be tested as outlined in Section 5.4.1.

Approved v3.0 930087119 3.0

5.5 Pharmacokinetic Assessments

It is extremely important to record the exact dose and time of the dose(s) taken the day prior to the visit/collection, and the exact date and time of the sample collection, even if drawn slightly off-schedule.

5.5.1 Intensive PK Assessment

A subset of subjects (approximately 12 subjects per treatment arm) will participate in an optional Intensive PK assessment at Week 2 (window Day 12 -16).

Intensive PK sample collection in this study will provide for the assessment of BMS-955176, TDF and FTC.

Intensive PK sampling begins with a pre-AM dose (0 hour) sampling, ie, prior to the administration of the morning doses of the blinded study drug and TDF/FTC on the day of the visit. The sampling should also begin 24 hours after the morning doses of the blinded study drug and TDF/FTC that were taken the day prior to the visit.

The subsequent 11 time points include samplings through Hour 12 (to be drawn prior to the administration of the evening dose of EFV/placebo). The subject will either stay overnight or will return to the clinic so that the final sample can be collected at Hour 24.

It is critical to capture the exact date and time of each PK sample collection, even if drawn slightly off-schedule. There is no specified collection window end for which any one time point should be abandoned as the schedule progresses. If a sample collection time point is missed/late and the next collection time point has not yet been reached, collect the missed time point, and record the exact time of that collection, then get back on track for the next time point/on-time collection.

Approved v3.0 930087119 3.0

Table 5.5.1-1 lists the sampling schedule to be followed for the assessment of intensive pharmacokinetics. Further details of PK blood collection and sample processing will be provided in the central clinical laboratory manual.

Table 5.5.1-1: AI468038 Intensive Pharmacokinetic Sampling Schedule at Week 2

	Time (Event)	Time (Relative to Dosing) Hour: Min	PK Blood Sample
Study Week 2 (window Day 12-16)	0 (morning pre-dose)	00:00	X
	0.5 Hr	00:30	X
	1 Hr	01:00	X
	1.5 Hr	01:30	X
	2 Hr	02:00	X
	4 Hr	04:00	X
	4.5 Hr	04:30	X
	5 Hr	05:00	X
	6 Hr	06:00	X
	8 Hr	08:00	X
	12 Hr (evening pre-dose)	12:00	X
	24 Hr (morning pre-dose)	24:00	X

5.5.2 *Sparse PK Assessments*

All subjects will provide Sparse PK samples (as part of the regular blood collection) for the assessment of BMS-955176, EFV, TDF and FTC at visit Weeks 4-24.

Of the five visits (Week 4 - 24), it is requested that the following guidelines are followed:

- At any one visit Week 4 through Week 24, the Sparse PK must be collected:
  - Approximately 24 hours (between 20 and 28 hours) *after* the doses of the BMS-955176 blinded study drug + TDF/FTC that were taken the morning before, and *before* the morning dose is taken on the day of the visit AND
  - 12 hours (between 10 and 14 hours) after the evening dose of EFV/placebo was taken the day before the visit
- At the remaining four visits Week 4 through Week 24, the blood collections may be done without any specific consideration to timing of previous dose administration (taken either the day before the visit or on the day of the visit), though it is critical that the date and time of

Approved v3.0 930087119 3.0

both the most previous morning doses of the blinded study drug and TDF/FTC and the evening dose of EFV/placebo taken the day before the visit) are recorded in the eCRF so that the exact interval between doses and blood sampling can be calculated for accurate PK analysis.

5.6 Biomarker Assessments

Samples will be collected at the screening visit for HIV-1 Gag sequencing, phenotypic susceptibility (PhenoSense Gag) and potential pharmacodiagnostic analysis as specified in Table 5.1-1. These samples will be analyzed, if deemed clinically relevant, as a predictive marker of clinical response.

Any remaining specimens that are available after completion of these designated analyses may be used in the future for identification of potentially predictive or pharmacodynamic markers of drug activity or to enhance the understanding around disease biology, except where prohibited by local laws or regulations.

5.7 Outcomes Research Assessments

Not applicable.

5.8 Other Assessments

Back-up plasma and serum samples will be obtained for current/future evaluation of the efficacy, safety and tolerability of BMS-955176.

Should any new safety signal develop during the course of the ongoing analysis of the Phase 2a trial, appropriate measures for evaluation and management will be incorporated into the design of the Phase 2b trial via a protocol amendment.

5.9 Results of Central Assessments

The following describes the centrally assessed parameters and the timing with which they will be shared with investigators, if pertinent. Some parameters are relevant to ongoing subject management during the study and will be provided to the site for such purpose, while others are not relevant to subject management during the study and results may only be shared in a summarized way at the end of the study.

- Samples sent to the central lab vendors for safety and efficacy assessments and that are tested real time will be provided to the sites as soon as results are available
- The results of the read of each ECG will be sent to the site by the central ECG vendor as soon as results are available
- Samples collected on-treatment for resistance testing will be tested if deemed clinically relevant (eg, if the development of resistance is suspected). If tested, results will be reported to the site

Approved v3.0 930087119 3.0

- Other samples (including but not limited to biomarker assessments, exploratory resistance, pharmacodiagnostics) may not be tested immediately, and may only be tested if deemed clinically relevant. Results may be suppressed from laboratory reports and may not be provided to the sites
- Individual PK results will not be reported to the site; the overall PK assessments will be included in the CSR

6 ADVERSE EVENTS

An ***Adverse Event (AE)*** is defined as any new untoward medical occurrence or worsening of a preexisting medical condition in a clinical investigation subject administered study drug and that does not necessarily have a causal relationship with this treatment. An AE can therefore be any unfavorable and unintended sign (such as an abnormal laboratory finding), symptom, or disease temporally associated with the use of study drug, whether or not considered related to the study drug.

The causal relationship to study drug is determined by a physician and should be used to assess all adverse events (AE). The causal relationship can be one of the following:

> Related: There is a reasonable causal relationship between study drug administration and the AE.
>
> Not related: There is not a reasonable causal relationship between study drug administration and the AE.

The term "reasonable causal relationship" means there is evidence to suggest a causal relationship.

Adverse events can be spontaneously reported or elicited during open-ended questioning, examination, or evaluation of a subject. (In order to prevent reporting bias, subjects should not be questioned regarding the specific occurrence of one or more AEs).

6.1 Serious Adverse Events

A S***erious Adverse Event (SAE)*** is any untoward medical occurrence that at any dose:

- results in death
- is life-threatening (defined as an event in which the subject was at risk of death at the time of the event; it does not refer to an event which hypothetically might have caused death if it were more severe)
- requires inpatient hospitalization or causes prolongation of existing hospitalization (see **NOTE** below)
- results in persistent or significant disability/incapacity
- is a congenital anomaly/birth defect
- is an important medical event (defined as a medical event(s) that may not be immediately life-threatening or result in death or hospitalization but, based upon appropriate medical and

Approved v3.0 930087119 3.0

scientific judgment, may jeopardize the subject or may require intervention [eg, medical, surgical] to prevent one of the other serious outcomes listed in the definition above). Examples of such events include, but are not limited to, intensive treatment in an emergency room or at home for allergic bronchospasm; blood dyscrasias or convulsions that do not result in hospitalization). Potential drug induced liver injury (DILI) is also considered an important medical event. (See Section 6.6 for the definition of potential DILI.)

Suspected transmission of an infectious agent (eg, pathogenic or nonpathogenic) via the study drug is an SAE.

Although pregnancy, overdose, cancer, and potential drug induced liver injury (DILI) are not always serious by regulatory definition, these events must be handled as SAEs. (See Section 6.1.1 for reporting pregnancies).

NOTE:

The following hospitalizations are not considered SAEs in BMS clinical studies:

- a visit to the emergency room or other hospital department < 24 hours, that does not result in admission (unless considered an important medical or life-threatening event)
- elective surgery, planned prior to signing consent
- admissions as per protocol for a planned medical/surgical procedure
- routine health assessment requiring admission for baseline/trending of health status (eg, routine colonoscopy)
- medical/surgical admission other than to remedy ill health and planned prior to entry into the study. Appropriate documentation is required in these cases
- admission encountered for another life circumstance that carries no bearing on health status and requires no medical/surgical intervention (eg, lack of housing, economic inadequacy, caregiver respite, family circumstances, administrative reason)
- Admission for administration of anticancer therapy in the absence of any other SAEs (applies to oncology protocols)

6.1.1 Serious Adverse Event Collection and Reporting

Sections 5.6.1 and 5.6.2 in the Investigator Brochure (IB) represent the Reference Safety Information to determine expectedness of serious adverse events for expedited reporting. Following the subject's written consent to participate in the study, all SAEs, whether related or not related to study drug, must be collected, including those thought to be associated with protocol-specified procedures. All SAEs must be collected that occur during the screening period and within 30 days of discontinuation of dosing.

The investigator should report any SAE that occurs after these time periods and that is believed to be related to study drug or protocol-specified procedure.

An SAE report should be completed for any event where doubt exists regarding its seriousness.

Approved v3.0 930087119 3.0

If the investigator believes that an SAE is not related to study drug, but is potentially related to the conditions of the study (such as withdrawal of previous therapy or a complication of a study procedure), the relationship should be specified in the narrative section of the SAE Report Form.

SAEs, whether related or not related to study drug, and pregnancies, must be reported to BMS (or designee) within 24 hours. SAEs must be recorded on the SAE Report Form; pregnancies on a Pregnancy Surveillance Form (electronic or paper forms). The preferred method for SAE data reporting collection is through the eCRF. The paper SAE/pregnancy surveillance forms are only intended as a back-up option when the eCRF system is not functioning. In this case, the paper forms are to be transmitted via email or confirmed facsimile (fax) transmission to:

SAE Email Address: Refer to Contact Information list.

SAE Facsimile Number: Refer to Contact Information list.

For studies capturing SAEs through electronic data capture (EDC), electronic submission is the required method for reporting. The paper forms should be used and submitted immediately, only in the event the electronic system is unavailable for transmission. When paper forms are used, the original paper forms are to remain on site.

SAE Telephone Contact (required for SAE and pregnancy reporting): Refer to Contact Information list.

If only limited information is initially available, follow-up reports are required. (Note: Follow-up SAE reports should include the same investigator term(s) initially reported.)

If an ongoing SAE changes in its intensity or relationship to study drug or if new information becomes available, a follow-up SAE report should be sent within 24 hours to the BMS (or designee) using the same procedure used for transmitting the initial SAE report.

All SAEs should be followed to resolution or stabilization.

6.2 Nonserious Adverse Events

A ***nonserious adverse event*** is an AE not classified as serious.

6.2.1 Nonserious Adverse Event Collection and Reporting

The collection of nonserious AE information should begin at initiation of study drug. Nonserious AE information should also be collected from the start of a placebo lead-in period or other observational period intended to establish a baseline status for the subjects.

Nonserious AEs should be followed to resolution or stabilization, or reported as SAEs if they become serious (see Section 6.1.1). Follow-up is also required for nonserious AEs that cause interruption or discontinuation of study drug and for those present at the end of study treatment as appropriate. All identified nonserious AEs must be recorded and described on the nonserious AE page of the CRF (paper or electronic).

Completion of supplemental CRFs may be requested for AEs and/or laboratory abnormalities that are reported/identified during the course of the study.

Approved v3.0 930087119 3.0

6.3 Laboratory Test Result Abnormalities

The following laboratory test result abnormalities should be captured on the nonserious AE CRF page or SAE Report Form (paper or electronic) as appropriate:

- Any laboratory test result that is clinically significant or meets the definition of an SAE
- Any laboratory test result abnormality that required the subject to have study drug discontinued or interrupted
- Any laboratory test result abnormality that required the subject to receive specific corrective therapy

It is expected that wherever possible, the clinical rather than laboratory term would be used by the reporting investigator (eg, anemia versus low hemoglobin value).

6.4 Pregnancy

If, following initiation of the study drug, it is subsequently discovered that a study subject is pregnant or may have been pregnant at the time of study exposure, including during at least 5 half lives after product administration, the investigator must immediately notify the BMS Medical Monitor/designee of this event and complete and forward a Pregnancy Surveillance Form to BMS Designee within 24 hours and in accordance with SAE reporting procedures described in Section 6.1.1.

The study drug will be permanently discontinued in an appropriate manner (eg, dose tapering if necessary for subject safety).

Protocol-required procedures for study discontinuation and follow-up must be performed on the subject unless contraindicated by pregnancy (eg, x-ray studies). Other appropriate pregnancy follow-up procedures should be considered if indicated.

Follow-up information regarding the course of the pregnancy, including perinatal and neonatal outcome and, where applicable, offspring information must be reported on the Pregnancy Surveillance Form.

Any pregnancy that occurs in a female partner of a male study participant should be reported to BMS. Information on this pregnancy will be collected on the Pregnancy Surveillance Form.

6.5 Overdose

An overdose is defined as the accidental or intentional administration of any dose of a product that is considered both excessive and medically important.

All occurrences of overdose must be reported as an SAE (see Section 6.1.1 for reporting details).

Approved v3.0 930087119 3.0

6.6 Potential Drug Induced Liver Injury (DILI)

Wherever possible, timely confirmation of initial liver-related laboratory abnormalities should occur prior to the reporting of a potential DILI event. All occurrences of potential DILIs, meeting the defined criteria, must be reported as SAEs (see Section 6.1.1 for reporting details).

Potential drug induced liver injury in HIV-1 mono-infected subjects is defined as:

1. AT (ALT or AST) elevation > 3 times upper limit of normal (ULN)

AND

2. Total bilirubin > 2 times ULN, without initial findings of cholestasis (elevated serum alkaline phosphatase),

AND

3. No other immediately apparent possible causes of AT elevation and hyperbilirubinemia, including, but not limited to, viral hepatitis, pre-existing chronic or acute liver disease, or the administration of other drug(s) known to be hepatotoxic

6.7 Other Safety Considerations

Any significant worsening noted during interim or final physical examinations, electrocardiogram, x-ray filming, any other potential safety assessment required or not required by protocol should also be recorded as a nonserious or serious AE, as appropriate, and reported accordingly.

6.7.1 Toxicity Management

The link to the current grading system for specific AEs and laboratory abnormalities is located in Appendix 3 (DAIDS; See Appendix 3).

6.7.1.1 Management of Elevations in Liver Transaminases

The following Table 6.7.1.1-1 summarizes the management of elevations in liver transaminases:

Table 6.7.1.1-1: Management of Elevations in Liver Transaminases Grade Level of AST or ALT Recommendations[a]

Grade	Recommendation
Grade1	None
Grade 2	none
Grade 3	Confirm elevation, evaluate for potential causes (including but not limited to alcohol or other substance abuse, concomitant medications, reactivation of existing or de novo infection with hepatitis viruses) consult with BMS Medical Monitor or designate as soon as possible.
Grade 4	Interrupt all study medications, evaluate for potential causes (including but not limited to alcohol or other substance abuse, concomitant medications, reactivation of existing or de novo infection

Approved v3.0 930087119 3.0

Table 6.7.1.1-1: Management of Elevations in Liver Transaminases Grade Level of AST or ALT Recommendations[a]

Grade	Recommendation
	with hepatitis viruses), monitor values frequently and consult with BMS Medical Monitor or designate as soon as possible. If Grade 4 is deemed related to study medication the subject must be discontinued from the study. Otherwise, if reinstitution of study therapy is considered, please obtain approval from the BMS Medical Monitor or designate.

[a] If persisting (> Grade 1/2), evaluate for alternative etiologies, including alcohol use and viral hepatitis

6.7.1.2 Management of Renal Toxicity

Serum phosphate levels and creatinine clearance (CrCl; CCL: as calculated by the Cockcroft Gault Equation [see Appendix 4] or by eGFR) should be monitored and managed as described in the Viread local package insert/label.[15] Dose interval adjustments of Viread are permitted, as described in Section 4.5.2.

6.7.1.3 Gastrointestinal Toxicity Evaluation and Management Plan

Pre-clinical toxicology studies in rats and dogs (see Section 1.4.1.2) have suggested a potential for GI related toxicity with BMS-955176. This section provides general guidance to the Investigator on the evaluation and management of primarily upper gastrointestinal symptoms. The Investigator may contact the Medical Monitor to discuss evaluation and management (including interruption of ARVs or discontinuation of a subject) of any GI symptoms throughout the trial.

Table 6.7.1.3-1: GI Toxicity Evaluation and Management

Tool	Recommendation
**HISTORY**	**For symptoms of all grades, a thorough history forms the foundation of proper evaluation and management. The following are potential manifestations of some GI clinical syndromes that may occur (possibly in combination) during the clinical trial.**
Nausea and Vomiting	The investigator should attempt to identify the etiology of these symptoms (and whether it is intraperitoneal, extraperitoneal, medication related, infection related, or due to a metabolic disorder).[16] Medications can cause nausea and vomiting acutely.
Dyspepsia	The Investigator should identify the presence of red flags (odynophagia, unexplained weight loss, recurrent vomiting, GI bleeding, jaundice, palpable mass or adenopathy, or family history of GI malignancy). Symptoms of dyspepsia could include early satiety, bloating, or belching. Additionally, atypical symptoms of dyspepsia could include: pharyngitis, asthma, bronchitis, hoarseness, chest pain, or abdominal pain.
Ulcerative Disease	Symptoms suggestive of ulceration often are intermittent over a period of weeks to months and may be relieved by eating or antacid use;[17] penetrating ulcers become more

Approved v3.0 930087119 3.0

Table 6.7.1.3-1: GI Toxicity Evaluation and Management

Tool	Recommendation
	acute with localized pain and may not improve with food.[18] The development of perforation may be indicated by severe diffuse abdominal pain.
Other Clinical Syndromes	Additional diagnostic criteria for other GI disorders potentially encountered in the clinical trial are available elsewhere.[19]
**PHYSICAL EXAMINATION**	**Physical examination should complement elements obtained from the history.[17] Acutely, the investigator may assess for signs of intravascular volume depletion (eg, orthostasis) and/or aspiration of vomitus as appropriate. Abdominal tenderness and guarding may indicate inflammation. The presence of fecal blood can indicate mucosal damage (eg, from an ulcer). Complete evaluation of dyspepsia should include an oral examination (poor dentition or pharyngeal erythema) and lungs for wheezing.**
**DIAGNOSTIC EVALUATION AND MANAGEMENT**	**A major goal in the diagnostic evaluation of a subject with upper GI symptoms is to quickly arrive at a final diagnosis without exposing the subject to unnecessary (invasive) testing; Investigators should exercise good clinical judgment[18] in this regard. A major goal of therapy is directed at correcting the underlying identifiable medical or surgical abnormalities. Consultation (eg, gastroenterologist) is recommended as clinically indicated.**
Grade 1 symptoms	Subjects may be treated symptomatically. If subjects develop dyspepsia alone, generally only limited and direct diagnostic testing should be performed.[17] If the subject has dyspepsia they should limit alcohol, caffeine, chocolate, tobacco, other contributing concomitant medications (eg, NSAIDs) and eating directly before bedtime. A variety of OTC medications are available to address constipation and diarrhea as indicated. Please refer to Appendix 1 for prohibited and precautionary medications.

Approved v 3.0 930087119 3.0

Table 6.7.1.3-1: GI Toxicity Evaluation and Management

Tool	Recommendation
Grade 2 symptoms[a]	Diagnostic testing may include but is not limited to the following (as clinically indicated):<br>• Serum chemistries and assessment of hemoglobin if not recently performed<br>• Testing for *Helicobacter pylori*<br>• Serologies (eg, celiac disease)<br>• PCR for viruses (eg, CMV)<br>• Iron panel or Vitamin B12 level<br>For subjects who have dyspepsia, a 4 week trial of a PPI may be attempted prior to testing for *H. pylori*. Note subjects must be off PPI therapy for 2 weeks prior to testing for *H. pylori* as PPI's can reduce bacterial load. During this 2-week period, an H2 receptor antagonist may be used.[18] Alternatively, Investigators can immediately test for *H. pylori* upon presentation. In either case when a negative result is encountered and a high clinical pre-test probability exists, a second test can be performed. For those who are *H. pylori* positive, combination treatment with antibacterials and an acid-suppressing agent is indicated. In cases of dyspepsia unresponsive to therapy, upper endoscopy may be indicated if not performed earlier.<br>The use of antiemetic's (eg, Prochlorperazine) can be utilized as indicated. Management should be targeted at addressing the underlying pathology.
Grade 3 symptoms[a]	Diagnostic testing may include but is not limited to the following (as clinically indicated):<br>• The testing outlined above in Grade 2<br>• A fasting serum gastrin level can be obtained in cases of known ulcers refractory to therapy, a family history of the disease, or when surgery is required; of note, *H. pylori* can increase gastrin levels.[18]<br>• A barium swallow to detect ulcers<br>• CT to identify gastrointestinal inflammation and a penetrating or perforated ulcer.<br>• Upper endoscopy with biopsy as indicated in order to evaluate dyspepsia further (eg, mucosal injury, new onset unexplained dyspepsia in subjects > 55 y/o, or the presence of red flags).<br>Management should be targeted at addressing the underlying pathology.

Approved v 3.0 930087119 3.0

Table 6.7.1.3-1: GI Toxicity Evaluation and Management

Tool	Recommendation
Grade 4 symptoms[a]	Diagnostic testing may include but is not limited to the following (as clinically indicated):<br>• The testing outlined above in Grade 2 and Grade 3<br>• An acute abdominal series<br>• If a perforated ulcer is clinically suspected, surgical consultation may be necessary<br>Initial management can include correction of hemodynamic and electrolyte abnormalities as clinically indicated. After stabilization, management should be targeted at addressing the underlying pathology.

[a] For Grade 2-4 symptoms if any ARV is thought to have a direct causal relationship to the patient's gastrointestinal symptoms, the Investigator should consider discontinuing the subject from the study and performing an evaluation/management plan incorporating elements above. The Investigator can consider interruption of the potential offending ARV(s) but must balance this with the increased probability of development of viral resistance/lack of efficacy. As stated above, prior to discontinuing the subject from the study, attempts should be made to discuss with the BMS Medical Monitor unless the safety of the subject is acutely at risk.

7 DATA MONITORING COMMITTEE AND OTHER EXTERNAL COMMITTEES

Not applicable

8 STATISTICAL CONSIDERATIONS

8.1 Sample Size Determination

This is an estimation study, without statistical testing, and hence there are no power considerations.

It is expected that the response rate for the primary endpoint will be somewhere around 80%. With this response rate, and 50 subjects per arm, an exact 95% confidence interval would run from roughly 66% to 90%.

8.2 Populations for Analyses

The following definitions are used in this document:

- Enrolled subjects: Subject who signed an informed consent form and were assigned a Patient Identification number (PID)
- Randomized subjects: Enrolled subjects who received a treatment assignment from the IVRS
- Treated subjects: Randomized subjects who received at least 1 dose of BMS-955176 or EFV. (Also referred to as the mITT analysis set)

Approved v3.0 930087119 3.0

8.3 Endpoints

8.3.1 Primary Endpoint(s)

The primary endpoint is the proportion of subjects with plasma HIV-1 RNA < 40 c/mL at Week 24. This will be assessed with the FDA snapshot algorithm. This uses the last on-treatment plasma HIV-1 RNA measurement within an FDA-specified visit window to determine response.

8.3.2 Secondary Endpoint(s)

- The antiviral efficacy will be determined by the proportion of subjects with plasma HIV-1 RNA < 40 c/mL at Weeks 48 and 96 using the FDA snapshot algorithm
- The antiviral efficacy will also be assessed by the proportion of subjects with plasma HIV-1 RNA < 200 c/mL at Weeks 24, 48, and 96 using the FDA snapshot algorithm approach with positive response defined as HIV-1 RNA < 200 c/mL
- The emergence of HIV drug resistance among samples selected for drug resistance testing will be assessed using the most recent version of the IAS-USA list of HIV-1 drug resistance mutations
- Changes from baseline in $\log_{10}$ HIV-1 RNA and in CD4+ T-cell counts, and changes in the percentage of CD4+ T-cells over time will be assessed using on-treatment laboratory results and pre-specified visit windows
- The frequency of SAEs and AEs leading to discontinuation (DC) will be tabulated directly from the case report forms (CRFs). The summary will count the number of subjects that have at least one event
- The occurrence of new AIDS defining events (CDC Class C events) will be tabulated from the CRFs. The summary will count the number of subjects that have at least one event
- The steady-state plasma PK of BMS-955176 will be assessed using the intensive PK data, collected at Week 2 from a subset of subjects

8.4 Analyses

In general, categorical variables are tabulated with counts and percents. Continuous variables are summarized with univariate statistics (eg, mean, median, standard error).

Longitudinal analyses use pre-defined visit week windows. Unless otherwise specified, windows around planned measurement times are constructed based on the midpoint between planned study visits (ie, half the duration of time between study visits), and data are summarized at each scheduled visit.

For the calculation of descriptive statistics of observed data, subjects must have a baseline measurement to be evaluable for longitudinal tabulations of parameter values and changes from baseline.

Approved v 3.0 930087119 3.0

Tabulations of the following endpoints present the number of unique subjects with an event: protocol deviations; interruptions of study therapy; non-study medications; adverse events; and laboratory abnormalities. Thus, multiple occurrences of the same event are counted only once per subject.

8.4.1 Demographics and Baseline Characteristics

The following demographic and baseline characteristics will be summarized by treatment arm and overall using the treated subjects:

- Demographics: age, race, ethnicity, gender, geographic region
- Disease characteristics at baseline: plasma HIV-1 RNA level, CD4+ T-cell counts and percentages, CD8+ T-cell counts, HIV-1 subtype
- Laboratory tests at baseline
- Pre-treatment CDC Class C AIDS events
- Prior medications

8.4.2 Efficacy Analyses

The efficacy analyses will be based on the treated subjects.

8.4.2.1 Primary Efficacy Analyses

The primary efficacy endpoint is the proportion of subjects with plasma HIV-1 RNA < 40 c/mL at the Week 24 snapshot. This endpoint is assessed with the FDA snapshot algorithm. The primary analysis will be based on a modified ITT (mITT) approach. A sensitivity analysis will be conducted using an observed values approach. The two approaches will be implemented as follows:

- Modified ITT: The numerator will be based on subjects with plasma HIV-1 RNA < 40 c/mL at Week 24. The denominator will be based on all treated subjects
- Observed values: Similar to the mITT approach, the numerator will be based on subjects with plasma HIV-1 RNA < 40 c/mL at Week 24. However, the denominator will be based on the treated subjects with plasma HIV-1 RNA at Week 24

Response rates will be tabulated by treatment arm with exact binomial 95% confidence intervals.

Subgroup summaries will be provided to examine the impact of baseline viral load and HIV-1 Clade AE (Clade AE versus Other) for both the mITT and the observed values approach. Other subgroup summaries may be provided to examine the impact of other important covariates such as CD4+ count, sex, geographic region, etc.

At Week 24, a Time to Loss of Virologic Response (TLOVR) analysis will be conducted as a sensitivity analysis that compliments the snapshot analysis. The following definition for virologic rebound will be used:

Approved v3.0 930087119 3.0

"For subjects that have been confirmed as having reached virologic suppression, by having two consecutive HIV-1 RNA readings below the assay limit of detection (40 copies/mL), virologic rebound is defined as confirmed HIV-1 RNA levels above the limit of detection. For levels above the limit of detection, confirmation consists of either two consecutive readings, or a single reading followed by loss to follow-up."

8.4.2.2 Secondary Efficacy Analyses

The following secondary endpoints will be summarized by treatment arm:

- Proportion of subjects with HIV-1 RNA < 40 c/mL at Week 48 and Week 96 using mITT and observed values
- Proportion of subjects with HIV-1 RNA < 200 c/mL at Week 24, 48, and 96 using mITT and observed values
- Change from baseline in $\log_{10}$ HIV-1 RNA and in CD4+ T-cell counts, and changes in the percentage of CD4+ T-cells over time
- Newly emergent genotypic substitutions (using all on-treatment isolates) will be tabulated by treatment arm
- The newly emergent phenotypic resistance profile (using all on-treatment isolates) will be tabulated by treatment arm

8.4.3 Safety Analyses

The investigators will determine the intensity of adverse events (AEs) and the relationship of AEs to study therapy. The investigators' terms will be coded and grouped by system organ class using the latest version of the Medical Dictionary for Regulatory Activities (MedDRA) in production at BMS. AEs will be presented by system organ class and preferred term. Presentations will include both non-serious and serious adverse events, unless specified otherwise. If a subject had an adverse event with different intensities over time, then only the greatest intensity will be reported.

Deaths will be listed for enrolled subjects without regard to onset.

In analyses of fasting lipids over time, values will be excluded after the start of serum lipid reducing agents.

The frequency of the following safety events will be summarized by treatment arm for treated subjects:

- SAEs
- AEs leading to discontinuation of study therapy
- AEs by intensity
- CDC Class C AIDS events
- Laboratory abnormalities by toxicity grade

Approved v3.0 930087119 3.0

8.4.4 Pharmacokinetic Analyses

The following PK parameters will be summarized for BMS-955176, TDF and/or FTC by treatment arm:

- C_{max}: maximum observed plasma concentration
- T_{max}: time of maximum observed plasma concentration
- C_{tau}: observed plasma concentration at the end of a dosing interval (eg, concentration at 24 hours)
- C_0: observed pre-dose plasma concentration
- AUC(TAU) : area under the concentration-time curve in one dosing interval

8.4.4.1 Sparse Pharmacokinetic Analyses

Sparse pharmacokinetic data will be used in population PK, PK/PD and PK/VK analyses.

8.4.4.2 PK/PD and PK/VK Analyses

PK data obtained from this study will be pooled with data from other studies to perform an integrated population PK analysis, exposure-response analyses for selected safety and efficacy endpoints, and viral kinetic modeling of BMS-955176 in combination with other ARVs to support the on-going development of BMS-955176. These analyses will facilitate optimal dose selection for future Phase 3 studies.

The population PK, exposure-response, and viral kinetic analyses will be reported separately.

8.4.5 Biomarker Analyses

Not applicable.

8.4.6 Outcomes Research Analyses

Not applicable.

8.4.7 Other Analyses (including Virologic Futility)

Virologic Futility:

An analysis of virologic futility will be performed at Week 24. This analysis will be conducted to evaluate whether any BMS-955176 arm shows significantly worse antiviral efficacy (HIV-1 RNA < 40 c/mL using the FDA snapshot algorithm) than the EFV containing arm. The comparison of Arms 1-3 (containing BMS-955176) to Arm 4 (containing EFV) will be made with one-sided, Fisher's exact tests, conducted at the 0.01 probability level.

Approved v3.0 930087119 3.0

8.5 Interim Analyses

An interim analysis will be conducted when approximately 50% of the subjects have completed 24 weeks of therapy. Access to the unblinded data will be restricted to a limited number of BMS and vendor personnel involved in the development of models for population pharmacokinetics, exposure-response relationships, and viral kinetics.

An interim analysis will be conducted after the last randomized subject is on study therapy for 24 weeks.

An interim analysis will be conducted after the last randomized subject is on study therapy for 48 weeks.

The final analysis will be conducted after the last randomized subject is on study therapy for 96 weeks.

9 STUDY MANAGEMENT

9.1 Compliance

9.1.1 Compliance with the Protocol and Protocol Revisions

The study shall be conducted as described in this approved protocol. All revisions to the protocol must be discussed with, and be prepared by, BMS. The investigator should not implement any deviation or change to the protocol without prior review and documented approval/favorable opinion from the IRB/IEC of an amendment, except where necessary to eliminate an immediate hazard(s) to study subjects.

If a deviation or change to a protocol is implemented to eliminate an immediate hazard(s) prior to obtaining IRB/IEC approval/favorable opinion, as soon as possible the deviation or change will be submitted to:

- IRB/IEC for review and approval/favorable opinion
- BMS
- Regulatory Authority(ies), if required by local regulations

Documentation of approval signed by the chairperson or designee of the IRB(s)/IEC(s) must be sent to BMS.

If an amendment substantially alters the study design or increases the potential risk to the subject: (1) the consent form must be revised and submitted to the IRB(s)/IEC(s) for review and approval/favorable opinion; (2) the revised form must be used to obtain consent from subjects currently enrolled in the study if they are affected by the amendment; and (3) the new form must be used to obtain consent from new subjects prior to enrollment.

If the revision is done via an administrative letter, investigators must inform their IRB(s)/IEC(s).

Approved v3.0 930087119 3.0

9.1.2 Monitoring

BMS representatives will review data centrally to identify potential issues to determine a schedule of on-site visits for targeted review of study records.

Representatives of BMS must be allowed to visit all study site locations periodically to assess the data quality and study integrity. On site they will review study records and directly compare them with source documents, discuss the conduct of the study with the investigator, and verify that the facilities remain acceptable. Certain CRF pages and/or electronic files may serve as the source documents:

In addition, the study may be evaluated by BMS internal auditors and government inspectors who must be allowed access to CRFs, source documents, other study files, and study facilities. BMS audit reports will be kept confidential.

The investigator must notify BMS promptly of any inspections scheduled by regulatory authorities, and promptly forward copies of inspection reports to BMS.

9.1.2.1 Source Documentation

The Investigator is responsible for ensuring that the source data are accurate, legible, contemporaneous, original and attributable, whether the data are hand-written on paper or entered electronically. If source data are created (first entered), modified, maintained, archived, retrieved, or transmitted electronically via computerized systems (and/or any other kind of electronic devices) as part of regulated clinical trial activities, such systems must be compliant with all applicable laws and regulations governing use of electronic records and/or electronic signatures. Such systems may include, but are not limited to, electronic medical/health records (EMRs/EHRs), adverse event tracking/reporting, protocol required assessments, and/or drug accountability records).

When paper records from such systems are used in place of electronic format to perform regulated activities, such paper records should be certified copies. A certified copy consists of a copy of original information that has been verified, as indicated by a dated signature, as an exact copy having all of the same attributes and information as the original.

9.1.3 Investigational Site Training

Bristol-Myers Squibb will provide quality investigational staff training prior to study initiation. Training topics will include but are not limited to: GCP, AE reporting, study details and procedure, electronic CRFs, study documentation, informed consent, and enrollment of WOCBP.

9.2 Records

9.2.1 Records Retention

The investigator must retain all study records and source documents for the maximum period required by applicable regulations and guidelines, or institution procedures, or for the period

Approved v3.0 930087119 3.0

specified by BMS, whichever is longer. The investigator must contact BMS prior to destroying any records associated with the study.

BMS will notify the investigator when the study records are no longer needed.

If the investigator withdraws from the study (eg, relocation, retirement), the records shall be transferred to a mutually agreed upon designee (eg, another investigator, IRB). Notice of such transfer will be given in writing to BMS.

9.2.2 Study Drug Records

It is the responsibility of the investigator to ensure that a current disposition record of study drug (inventoried and dispensed) is maintained at the study site to include the investigational product and the non-investigational product(s). Records or logs must comply with applicable regulations and guidelines and should include:

- amount received and placed in storage area
- amount currently in storage area
- label identification number or batch number
- amount dispensed to and returned by each subject, including unique subject identifiers
- amount transferred to another area/site for dispensing or storage
- nonstudy disposition (eg, lost, wasted)
- amount destroyed at study site, if applicable
- amount returned to BMS
- retain samples for bioavailability/bioequivalence, if applicable
- dates and initials of person responsible for Investigational Product dispensing/accountability, as per the Delegation of Authority Form

BMS will provide forms to facilitate inventory control if the investigational site does not have an established system that meets these requirements.

9.2.3 Case Report Forms

An investigator is required to prepare and maintain adequate and accurate case histories designed to record all observations and other data pertinent to the investigation on each individual treated or entered as a control in the investigation. Data that are derived from source documents and reported on the CRF must be consistent with the source documents or the discrepancies must be explained. Additional clinical information may be collected and analyzed in an effort to enhance understanding of product safety. CRFs may be requested for AEs and/or laboratory abnormalities that are reported or identified during the course of the study.

For sites using the BMS electronic data capture tool, electronic CRFs will be prepared for all data collection fields except for fields specific to SAEs and pregnancy, which will be reported on the paper or electronic SAE form and Pregnancy Surveillance form, respectively. Spaces may be

Approved v3.0 930087119 3.0

left blank only in those circumstances permitted by study-specific CRF completion guidelines provided by BMS.

The confidentiality of records that could identify subjects must be protected, respecting the privacy and confidentiality rules in accordance with the applicable regulatory requirement(s).

The investigator will maintain a signature sheet to document signatures and initials of all persons authorized to make entries and/or corrections on CRFs.

The completed CRF, including any paper or electronic SAE/pregnancy CRFs, must be promptly reviewed, signed, and dated by the investigator or qualified physician who is a subinvestigator and who is delegated this task on the Delegation of Authority Form. For electronic CRFs, review and approval/signature is completed electronically through the BMS electronic data capture tool. The investigator must retain a copy of the CRFs including records of the changes and corrections.

Each individual electronically signing electronic CRFs must meet BMS training requirements and must only access the BMS electronic data capture tool using the unique user account provided by BMS. User accounts are not to be shared or reassigned to other individuals.

9.3 Clinical Study Report and Publications

A Signatory Investigator must be selected to sign the clinical study report.

For this protocol, the Signatory Investigator will be selected as appropriate based on the following criteria:

- External Principal Investigator designated at protocol development
- National Coordinating Investigator
- Study Steering Committee chair or their designee
- Subject recruitment (eg, among the top quartile of enrollers)
- Involvement in trial design
- Regional representation (eg, among top quartile of enrollers from a specified region or country)
- Other criteria (as determined by the study team)

The data collected during this study are confidential and proprietary to BMS. Any publications or abstracts arising from this study require approval by BMS prior to publication or presentation and must adhere to BMS's publication requirements as set forth in the approved clinical trial agreement (CTA). All draft publications, including abstracts or detailed summaries of any proposed presentations, must be submitted to BMS at the earliest practicable time for review, but at any event not less than 30 days before submission or presentation unless otherwise set forth in the CTA. BMS shall have the right to delete any confidential or proprietary information contained in any proposed presentation or abstract and may delay publication for up to 60 days for purposes of filing a patent application.

Approved v3.0 930087119 3.0

10 GLOSSARY OF TERMS

Term	Definition
Complete Abstinence	If one form of contraception is required, Complete Abstinence is defined as complete avoidance of heterosexual intercourse and is an acceptable form of contraception for all study drugs. Female subjects must continue to have pregnancy tests. Acceptable alternate methods of highly effective contraception must be discussed in the event that the subject chooses to forego complete abstinence.<br><br>If two forms of contraception is required, Complete abstinence is defined as complete avoidance of heterosexual intercourse and is an acceptable form of contraception for all study drugs. Subjects who choose complete abstinence are not required to use a second method of contraception, but female subjects must continue to have pregnancy tests. Acceptable alternate methods of highly effective contraception must be discussed in the event that the subject chooses to forego complete abstinence.<br><br>**Expanded definition** Complete abstinence as defined as complete avoidance of heterosexual intercourse is an acceptable form of contraception for all study drugs. This also means that abstinence is the preferred and usual lifestyle of the patient. This does not mean periodic abstinence (eg, calendar, ovulation, symptothermal, profession of abstinence for entry into a clinical trial, post-ovulation methods) and withdrawal, which are not acceptable methods of contraception. Subjects who choose complete abstinence are not required to use a second method of contraception, but female subjects must continue to have pregnancy tests. Acceptable alternate methods of highly effective contraception must be discussed in the event that the subject chooses to forego complete abstinence

Approved v3.0 930087119 3.0

11 LIST OF ABBREVIATIONS

Term	Definition
3TC	Lamivudine
AE	adverse event
AI	accumulation index
AIDS	Acquired Immunodeficiency Syndrome
AI_AUC	AUC Accumulation Index; ratio of AUC(TAU) at steady state to AUC(TAU) after the first dose
AI_C_{max}	C_{max} Accumulation Index; ratio of C_{max} at steady state to C_{max} after the first dose
AI_C_{tau}	C_{tau} Accumulation Index; ratio of C_{tau} at steady state to C_{tau} after the first dose
ALT	alanine aminotransferase
ARV	antiretroviral
ART	Antiretroviral therapy
AST	aspartate aminotransferase
ATV	atazanavir
ATV/r	atazanavir boosted with ritonavir
AUC	area under the concentration-time curve
AUC(INF)	area under the concentration-time curve from time zero extrapolated to infinite time
AUC(0-T)	area under the concentration-time curve from time zero to the time of the last quantifiable concentration
AUC(TAU)	area under the concentration-time curve in one dosing interval
A-V	atrioventricular
β-HCG	beta-human chorionic gonadotrophin
BA/BE	bioavailability/bioequivalence
BID, bid	bis in die, twice daily
BCRP	Breast cancer reactive protein
BDC	Bile duct-cannulated
BMI	body mass index
BMS	Bristol-Myers Squibb

Approved v3.0 930087119 3.0

Term	Definition
BP	blood pressure
BVM	bevirimat
c	copies
c/mL	copies per milliliter
C	Celsius
C12	concentration at 12 hours
C24	concentration at 24 hours
CA	capsid
cART	Combination antiretroviral therapy
Cavg	average concentration
Cexpected-tau	expected concentration in a dosing interval
CD	Cluster designation (CD4; CD8)
CDC	Centers for Disease Control
CFC	corrected fold change
CFR	Code of Federal Regulations
CI	confidence interval
CrCl	creatinine clearance
CLR	renal clearance
C_{max}, CMAX	maximum observed concentration
C_{min}, CMIN	trough observed concentration
CMV	cytomegalovirus
CNS	Central nervous system
CRC	Clinical Research Center
CRF	Case Report Form, paper or electronic
C_{ss},avg	average steady-state plasma concentration
CSR	Clinical study report
Ct	Expected concentration at a certain time, usually at the end of an expected future dosing interval (eg, concentration at 24 hours, concentration at 12 hours, etc.)
C_{tau}	Concentration in a dosing interval (eg, concentration at 24 hours, concentration at 12 hours, etc.)

Approved v 3.0 930087119 3.0

Term	Definition
C_{trough}	Trough observed plasma concentration
CT	Computed tomography
CTA	clinical trial agreement
CTX	Cross-linked C-telopeptide of Type 1 collagen
CYP	cytochrome p-450
D/C	discontinue
DDI	drug-drug interaction
DHHS	Department of Health and Human Services
dL	deciliter
EC	Ethics committee
EC	effective concentration
ECG	electrocardiogram
eCRF	Electronic Case Report Form
EDC	Electronic Data Capture
EFD	Embryo-fetal development
EFV	efavirenz
eg	exempli gratia (for example)
E-R	exposure-response
ESR	Expedited Safety Report
ET	Early termination or End of Treatment
EU	European Union
FAHI	Functional Assessment of HIV Infection
FDA	Food and Drug Administration
FDC	Fixed dose combination
FSH	follicle stimulating hormone
FTC	Emtricitabine
g	gram
GFR	glomerular filtration rate
GCP	Good Clinical Practice
GGT	gamma-glutamyl transferase

Approved v 3.0 930087119 3.0

Term	Definition
GFR	glomerular filtration rate
GSH	glutathione
h; hr	hour
HAART	highly active antiretroviral therapy
HBsAg	hepatitis B surface antigen
HBV	hepatitis B virus
HCG	Human chorionic gonadotropin
HCV	hepatitis C virus
HCO3-	bicarbonate
HIPAA	Health Insurance Portability and Accountability Act
HIV	Human Immunodeficiency Virus
HR	heart rate
HRT	hormone replacement therapy
HS	Human serum
HuSA	Human serum albumin
IAS	International AIDS Society
IB	Investigator brochure
IC	Inhibitory concentration
ICD	International Classification of Diseases
ICF	informed consent form
ICH	International Conference on Harmonisation
ie	id est (that is)
IEC	Independent Ethics Committee
IMP	investigational medicinal products
IND	Investigational New Drug Exemption
INI	Integrase inhibitor
IP	investigational product
IRB	Institutional Review Board
IU	International Unit
IUD	intrauterine device

Approved v3.0 930087119 3.0

Term	Definition
IV	intravenous
IVRS	interactive voice response system
GALT	Gut associated lymphoid tissue
GI	gastrointestinal
kg	kilogram
L	liter
MAD	multiple ascending dose
MC	micronized crystalline
mg	milligram
MI	Maturation inhibitor
MIC	minimum inhibitory concentration
min	minute
mITT	Modified Intent to Treat
mL	milliliter
mmHg	millimeters of mercury
msec	millisecond
MOA	mechanism of action
μg	microgram
μM	micromolar
N	number of subjects or observations
N/A	not applicable
ng	nanogram
nM	nanomolar
NIMP	non-investigational medicinal products
NNRTI	Non- nucleoside reverse transcriptase inhibitor
NOAEL	No observed adverse effect level
NOEL	No observed effect level
NRTI	nucleoside reverse transcriptase inhibitor
NSAID	nonsteroidal anti-inflammatory drug
pDILI	potential drug induced liver injury

Approved v3.0 930087119 3.0

**Term**	**Definition**
PBMC	peripheral blood mononuclear cells
PCR	Polymerase chain reaction
PD	pharmacodynamics
PDVF	Protocol-defined virologic criteria
PI	protease inhibitor
PK	pharmacokinetics
PPI	proton pump inhibitor
PR	atrial depolarization to ventricular depolarization
PT	prothrombin time
PTT	partial thromboplastin time
QC	quality control
QD, qd	quaque die, once daily
QRS	interval representing the time for ventricular depolarization
QT	Duration of ventricular electrical activity
QTcF	QT corrected for heart rate using Frederica's formula
RBC	red blood cell
RNA	ribonucleic acid
RTV	ritonavir
SAD	single ascending dose
SDD	spray-dried dispersion
SAE	serious adverse event
SAP	statistical analysis plan
SD	standard deviation
SOP	Standard Operating Procedures
SP1	spacer peptide 1
Subj	subject
STR	single tablet regimen
t	temperature
T	time
TAMs	Thymidine analog mutations

Approved v3.0 930087119 3.0

Term	Definition
TD	Tenofovir disoproxil
TDF	Tenofovir disoproxil fumarate
TD/FTC	Truvada (TD + FTC 245 mg/ 200 mg)
TDF/FTC	Truvada (TDF + FTC 300 mg/ 200 mg)
TAO	Trial Access Online, the BMS implementation of an EDC capability
T-HALF	Half life
T-HALFeff_AUC	Effective elimination half life that explains the degree of AUC accumulation observed
T_{max}, TMAX	time of maximum observed concentration
TR_AUC(0-T)	AUC(0-T) treatment ratio
TR_AUC(INF)	AUC(INF) treatment ratio
TR_Cmax	Cmax treatment ratio
UGT	UDP-glucuronosyltransferase
ULN	upper limit of normal
US; USA	United States; United States of America
VF	virologic failure
VK	Viral kinetics
VLP	Virus-like particles
WBC	white blood cell
WFD	Wallingford, Connecticut, USA
WHO	World Health Organization
Wk or WK	week
WOCBP	women of childbearing potential

Approved v3.0 930087119 3.0

12 REFERENCES

1 WHO HIV Department. Global Summary of the AIDS Epidemic 2013. Accessed Dec. 29, 2014 . Available at: http://www.who.int/hiv/data/epi_core_dec2014.png?ua=1

2 Panel on Antiretroviral Guidelines for Adults and Adolescents. Guidelines for the use of antiretroviral agents in HIV-1-infected adults and adolescents. Department of Health and Human Services. Accessed Nov 30, 2013 http://aidsinfo.nih.gov/contentfiles/lvguidelines/AdultandAdolescentGL.pdf.

3 United States Department of Health and Human Services, Food and Drug Administration, Center for Drug Evaluation and Research. Guidance for Industry Human Immunodeficiency Virus-1 Infection: Developing Antiretroviral Drugs for Treatment. (June 2013). Revision 1

4 European Medicines Agency. Guideline on the Clinical Development of Medicinal Products for the Treatment of HIV Infection. (Sept 13). EMEA/CPMP/EWP/633/02; Rev 3

5 Gupta S.K., Lombaard J., Echevarria J., et. al. HIV NRTI BMS-986001 in Antiretroviral-Naive Subjects: Week 24/49 Analyses. ICAAC 2014 Oral Abstract: H-642

6 Study AI468001 Randomized, Double-Blinded, Placebo-Controlled, Single and Multiple Ascending Dose Study to Evaluate the Safety, Tolerability, and Pharmacokinetics of BMS-955176 in Healthy Subjects. Document Control No. 930071044

7 Study AI468002 Randomized, Placebo-Controlled, Multiple-Dose Study to Evaluate the Pharmacodynamics, Safety and Pharmacokinetics of BMS-955176 (Double-Blinded) and BMS-955176 with Atazanavir +/- Ritonavir (Open-Labelled) in HIV-1 Infected Subjects. Draft Document Control No. 930087017

8 European AIDS Clinical Society. European Guidelines for treatment of HIV-infected adults in Europe V 7.1 (Nov 2014). Accessed December 2014. EACS Guidelines

9 BMS-955176 Investigator Brochure, Version 3, April 17, 2014 DCN 930056146

10 Partial response to FDA “May Proceed” Letter dated 27 Sep 2013 for IND 118,936. Bristol-Myers Squibb Company; Jan 2014. Document Control No. 930076507 2.0. Insert this additional reference: “Evaluation of cross-resistance of HIV-1 Maturation Inhibitor BMS-955176 toward HIV-1 protease inhibitor resistant viruses. Bristol-Myers Squibb Company; 30-Sept-2014. Document Control No. 930083565.”

11 Evaluation of Cross Resistance of HIV-1 Maturation Inhibitor BMS-955176 Toward HIV-1 Protease Inhibitor Resistant Viruses. Document Control No. 930083565

12 Sustiva USPI. Accessed January 6, 2014. http://packageinserts.bms.com/pi/pi_sustiva.pdf

Approved v 3.0 930087119 3.0

13 Truvada (TDF/FTC) USPI. Accessed January 6, 2014. http://www.gilead.com/~/media/Files/pdfs/medicines/hiv/truvada/truvada_pi.PDF

14 Evaluation of Cross-Resistance of HIV-1 Maturation Inhibitor BMS-955176 Toward HIV-1 Protease Inhibitor Resistant Viruses. DCN 930083565

15 Gilead Sciences. Prescribing Information for TDF. Available at: http://www.gilead.com/~/media/Files/pdfs/medicines/liver-disease/viread/viread_pi.pdf Accessed Dec 31, 2014

16 Hasler WL Nausea, Vomiting, and Indigestion: Introduction. Chapter 39. Harrisons Principles of Internal Medicine 18th edition. 2012. McGraw Hill

17 Hasler WL and Owyang C Approach to the Patient with Gastrointestinal Disease. Chapter 290. Harrisons Principles of Internal Medicine 18th edition. 2012. McGraw Hill

18 Soll AH and Graham DY. Peptic Ulcer Disease. Chapter 40. Textbook of Gastroenterology. 5th edition. 2009. Blackwell Publishing

19 Rome Foundation. Rome III Diagnostic Criteria for Functional Gastrointestinal Disorders. Accessed Dec 22 2014. Available at: http://www.romecriteria.org/assets/pdf/19_RomeIII_apA_885-898.pdf

Approved v3.0 930087119 3.0

APPENDIX 1 LISTINGS OF PROHIBITED AND PRECAUTIONARY THERAPIES

General Notes:

- Guidelines for the use of drugs with established or other potentially significant drug interactions listed in the Package Inserts of the marketed ARV agents used by subjects participating in this study (Efavirenz/Sustiva®, TDF/FTC) should be followed.
- Medications listed in the Package Inserts as contra-indicated with the other marketed ARV agents used by subjects participating in this study are not permitted.
- Any immunizations deemed appropriate by the subject's physician are permitted provided that the immunization is given > 4 weeks from any HIV-1 RNA measurement.
- A subject may not be co-enrolled in a concomitant trial unless it is approved by the Medical Monitor prior to randomization.

Prohibited Therapies

Drugs that should not be administered throughout the duration of the study:

Anticonvulsants: Carbamazepine, Phenobarbital, Phenytoin	Use with EFV may result in decreased concentrations of anticonvulsants or EFV.
Oral Antifungals: Itraconazole, Posaconazole, and Voriconazole	Use with EFV can result in decreased concentrations of antifungals
Antimycobacterials: Rifampin, Rifapentine, Rifabutin	Use with BMS-955176 can result in decreased concentrations of BMS-955176
St. John's wort	Use with EFV may result in loss of antiviral therapeutic effect
GI motility agent: Cisapride	Potential for serious and/or life threatening reactions such as cardiac arrhythmias
Pimozide	Potential for serious and/or life threatening reactions such as cardiac arrhythmias
Zetia (ezetimibe)	Ezetimibe is a substrate of OATP1B1 (of which BMS-955176 is an inhibitor in vitro).
HMG-CoA Reductase Inhibitors: Lovastatin, Simvastatin, Atorvastatin, Pitavastatin, Rosuvastatin, Pravastatin	HMG-CoA Reductase Inhibitors are substrates of OATP1B1/B3 (of which BMS-955176 is an inhibitor in vitro)
All drugs with antiretroviral activity other than those considered study therapy	Any drugs with antiretroviral activity not considered study therapy may interfere with the assessments of the study.

Approved v 3.0 930087119 3.0

Precautionary Therapies

Drugs that should be administered with caution during the study:

Hormonal Contraceptives	Hormonal Contraceptives cannot be relied upon as a highly effective method of contraception.<br>See Protocol Section 3.3.1, for more information on Highly Effective Methods of Contraception.
Antidepressants: Bupropion and Sertaline	Use with EFV can result in decreased concentrations of Bupropion or Sertaline
Macrolides: Clarithromycin	Use with EFV can result in decreased concentrations of Clarithromycin
Antimalarials: Artemether/Lumefantrine, Atovaquone/Proguanil	Use with EFV can result in decreased concentrations of Antimalarials.
Calcium Channel Blockers	Use with EFV may result in decreased concentrations of CCB's.
Benzodiazepines: Triazolam and Midazolam	Use with EFV can result in increased concentrations of Benzodiazepines.
Dexamethasone	Use with EFV may result in reduced levels of EFV.
Avanafil	Use with EFV may result in decreased levels of Avanafil

Approved v 3.0 930087119 3.0

APPENDIX 2 AIDS-DEFINING DIAGNOSES

I. PARASITIC INFECTIONS

Pneumocystis carinii (PC)

1011 PC pneumonia histologically proven.

1012 PC pneumonia, clinical diagnosis by the following specifications and confirmed HIV infection:

A history of dyspnea on exertion or non-productive cough of recent onset (within the past 3 months).

AND

Chest X-ray evidence of diffuse bilateral interstitial or gallium scan evidence of diffuse bilateral pulmonary disease;

AND

Arterial blood gas analysis showing an arterial pO2 of < 70 mmHg or a low respiratory diffusing capacity (< 80% of predicted values) or an increase in the alveolar-arterial oxygen tension gradient;

AND

Successful response to appropriate therapy and no evidence of pneumonias of other etiologies.

1013 Pneumocystis carinii, histologically proven, at a site other than lungs.

Toxoplasmosis (in patients > 1 month old)

1021 Toxoplasmosis, clinical diagnosis (of brain only) by the following specifications and confirmed HIV infection:

Recent onset of a neurologic disease consistent with toxoplasmosis;

AND

Brain imaging evidence of a mass lesion (on computed tomography, nuclear magnetic resonance of radiography enhanced by injection of contrast medium);

AND

Serum antibody to toxoplasmosis and successful response to therapy for toxoplasmosis.

1022 Toxoplasmosis, of brain or internal organs other than liver, spleen or lymph nodes. Proven by microscopy.

Isosporiasis

1031 Isosporiasis causing chronic diarrhea of > 1 month. Proven by microscopy.

Approved v3.0 930087119 3.0

Cryptosporidiosis

1041 Cryptosporidiosis causing chronic diarrhea of > 1 month. Proven by microscopy.

II. FUNGAL INFECTIONS

Candidiasis

2011 Candidiasis, Esophageal, definitive diagnosis by the following specifications:

Gross inspection by endoscopy or autopsy or by microscopy (histology or cytology) on a specimen obtained directly from the tissues affected (including scrapings from the mucosal surface), not from a culture.

2012 Candidiasis, Esophageal, presumptive diagnosis by the following specifications and confirmed HIV infection:

Recent onset of retrosternal pain on swallowing:

AND

Oral candidiasis diagnosed by the gross appearance of white patches or plaques on an erythematous base OR by the microscopic appearance of fungal mycelial filaments in an uncultured specimen scraped from the oral mucosa;

AND

Response to appropriate therapy.

2013 Candidiasis, Bronchial/Pulmonary, definitive diagnosis by the following specifications; Gross inspection by endoscopy or autopsy or by microscopy (histology or cytology) on a specimen obtained directly from the tissues affected (including scrapings from the mucosal surface), not from a culture.

Cryptococcosis

2022 Cryptococcosis, Extra-pulmonary, proven by microscopy (histology or cytology), culture or detection of antigen in a specimen obtained directly from the tissues affected or a fluid from those tissues.

Histoplasmosis

2031 Histoplasmosis, Disseminated (at a site other than or in addition to lungs or cervical or hilar lymph nodes), proven by microscopy (histology or cytology), culture or detection of antigen in a specimen obtained directly from the tissues affected or a fluid from those tissues.

Coccidioidomycosis

2041 Coccidioidoidomycosis, Disseminated (at a site other than or in addition to lungs or cervical or hilar lymph nodes), proven by microscopy (histology or cytology), culture or detection of antigen in a specimen obtained directly from the tissues affected or a fluid from those tissues.

Approved v3.0 930087119 3.0

2042 Coccidioidomycosis, clear reactivation of prior infection, proven by microscopy (histology or cytology), culture or detection of antigen in a specimen obtained directly from the tissues affected or a fluid from those tissues.

III. BACTERIAL INFECTIONS

Mycobacterium

3001 Mycobacterium (unidentified species). Presumptive diagnosis, by the following specifications and confirmed HIV infection.

Acid fast bacilli (AFB) positive stain of specimen obtained from endoscopic biopsy or from a normal sterile site other than lungs, skin or cervical or hilar lymph nodes. Species NOT identified by culture.

Mycobacterium tuberculosis

3011 Mycobacterium tuberculosis, Pulmonary, definitive diagnosis proven by culture, without evidence of upper respiratory infection symptoms of Mycobacterium tuberculosis that could account for the positive culture.

3012 Mycobacterium tuberculosis, definitive diagnosis proven by culture, of at least one extra pulmonary site regardless of concurrent pulmonary involvement.

3013 Mycobacterium tuberculosis, Disseminated, definitive diagnosis proven by culture.

Mycobacterium avium intracellulare

3022 MAI in Blood, proven by culture.

3023 MAI Colitis, proven by histology and culture. (This does not include MAI of the stool alone).

3024 MAI, Disseminated, at a site other than or in addition to lungs or cervical or hilar lymph nodes, proven by culture.

Mycobacterium Kansasii, Mycobacterium Scrofulaceum and Other Atypical Mycobacterium

3032 M. Kansasii, in Blood, proven by culture.

3033 M. Kansasii Colitis, proven by histology and culture. (NOT including positive M. Kansasii of stool alone).

3034 M. Kansasii, Disseminated, at a site other than or in addition to lungs, or cervical or hilar lymph nodes, proven by culture.

3035 M. Scrofulaceum or other Atypical Mycobacterium, proven by culture.

Salmonella

3041 Salmonella, recurrent Bacteremia (non-typhoid), proven by culture.

Approved v3.0 930087119 3.0

IV. VIRAL INFECTIONS

Cytomegalovirus

4011 CMV, Pneumonitis, pathologically or histologically confirmed. Serum antibody titer and culture alone is not sufficient for the diagnosis.

4012 CMV, Esophagitis, as diagnosed by histology, pathology or culture of an esophageal lesion. Serum antibody titer and culture of other than esophageal tissue is not sufficient for the diagnosis.

4013 CMV, Retinitis as evidenced by a characteristic appearance on serial ophthalmoscopic examinations (eg, discrete patches of retinal whitening with distinct borders, spreading in a centrifugal manner, following blood vessels, progressing over several months, frequently associated with retinal vasculitis, hemorrhage, and necrosis). Resolution of active disease leaves retinal scarring and atrophy with retinal pigment epithelial mottling.

4014 CMV, Colitis, as diagnosed by histology, pathology or culture of a colonic lesion. Serum antibody titer and culture of other than colonic tissue is not sufficient for the diagnosis.

4015 CMV, Encephalitis, as diagnosed by histology, pathology or culture of brain tissue or CSF. Serum antibody titer and culture of other than brain tissue or CSF is not sufficient for the diagnosis.

Herpes Simplex (in patients > 1 month old).

4021 HSV, Disseminated (but not encephalitis alone), proven by microscopy (histology or cytology), culture or detection of antigen in a specimen obtained directly from affected tissues.

4022 HSV, Esophagitis, as diagnosed by microscopy (histology or cytology), culture or detection of antigen in a biopsy specimen obtained directly from affected tissue. Serological measurement and culture from other than the affected tissue is not sufficient for the diagnosis.

4023 HSV, Bronchitis, as diagnosed by microscopy (histology or cytology), culture or detection of antigen in a biopsy specimen obtained directly from affected tissue. Serological measurement and culture from other than the affected tissue is not sufficient for the diagnosis.

4024 HSV, Pneumonitis, as diagnosed by microscopy (histology or cytology), culture or detection of antigen in a biopsy specimen obtained directly from affected tissue. Serological measurement and culture from other than the affected tissue is not sufficient for diagnosis.

4025 HSV, GI, other than mouth, throat, or peri-rectal, as diagnosed by microscopy (histology or cytology), culture or detection of antigen in a biopsy specimen obtained directly from affected tissue. Serological measurement and culture from other than the affected tissue is not sufficient for diagnosis.

Approved v3.0 930087119 3.0

4026 HSV, Mucocutaneous, ulcers persisting for ≥ 1 month despite appropriate therapy, as diagnosed by microscopy (histology or cytology), culture or detection of antigen in a biopsy specimen obtained directly from affected tissue. Serological measurement and culture from other than the affected tissue is not sufficient for the diagnosis.

Progressive Multifocal Leukoencephalopathy

4041 Progressive Multifocal Leukoencephalopathy, proven by microscopy.

VI. NEOPLASTIC DISEASES

Kaposi's Sarcoma

6011 Kaposi's sarcoma, Mucocutaneous, proven by microscopy.

6012 Kaposi's sarcoma. Mucocutaneous, presumptive diagnosis with characteristic gross appearance and confirmed HIV infection.

6013 Kaposi's sarcoma, Visceral.

6014 Kaposi's sarcoma, other than above.

Lymphoma of the Brain

6021 Primary Lymphoma of the brain at any age, proven by microscopy.

Non-Hodgkins Lymphoma

6031 Small Non-cleaved lymphoma (either Burkitt or non-Burkitt type).

6032 Immunoblastic sarcoma, equivalent to any of the following, although not necessarily all in combination: Immunoblastic lymphoma, large-cell lymphoma, diffuse histocytic lymphoma.

Cervical Carcinoma

6041 Histologically proven invasive carcinoma of the cervix.

VII. OTHER CONDITIONS

HIV Dementia/Motor Defects

7011 HIV Dementia, clinical findings of disabling cognitive and/or motor dysfunction interfering with occupation or activities of daily living progressing over weeks to months, in the absence of a concurrent illness or condition other than HIV infection that could explain the findings. Method to rule out such concurrent illnesses and conditions must include cerebrospinal fluid examination and either brain imaging (computed tomography or magnetic resonance) or autopsy.

Slim Disease or HIV Wasting Syndrome

7021 HIV Wasting Syndrome, findings of profound involuntary weight loss > 10% of baseline body weight plus either chronic diarrhea (at least two loose stools per day for ≥ 30 days) or chronic weakness and documented fever (for ≥ 30 days, intermittent to constant) in the

Approved v3.0 930087119 3.0

absence of a concurrent illness or condition other than HIV infection that could explain the findings (eg, cancer, tuberculosis, cryptosporidiosis, or other specific enteritis).

7061 Recurrent pneumonia, acute onset within 12 months of most recent episode.

Approved v3.0 930087119 3.0

APPENDIX 3 DAIDS TOXICITY GRADES

Division of AIDS (DAIDS) Table for Grading the Severity of Adult and Pediatric Adverse Events

Version 2.0 - November 2014

http://rsc.tech-res.com/safetyandpharmacovigilance/gradingtables.aspx

Approved v 3.0 930087119 3.0

APPENDIX 4 COCKCROFT-GAULT EQUATION TO CALCULATE SERUM CREATINE CLEARANCE

Online calculator:

http://nephron.com/cgi-bin/CGSI.cgi

Manual calculation:

Male:

$$\text{Estimated Creatinine Clearance} = \frac{(140\text{-age in years}) \times \text{Body Weight (kg)}}{72 \times \text{Serum Creatinine (mg/dL)}}$$

Female:

$$\text{Estimated Creatinine Clearance} = \frac{(140\text{-age in years}) \times \text{Body Weight (kg)} \times 0.85}{72 \times \text{Serum Creatinine (mg/dL)}}$$

1 pound = 0.4536 kilograms

Approved v3.0 930087119 3.0

APPENDIX 5 LABORATORY ASSESSMENTS

HEMATOLOGY		
WBC	Screening	Day 1, Week 4, Week 8, Week 12, Week 16, Week 24, Week 32, Week 40, Week 48, Week 60, Week 72, Week 84, Week 96, Early Term
RBC		
Hemoglobin		
Hematocrit		
Platelets		
Absolute Differential		
CHEMISTRY		
Glucose	Screening	Day 1, Week 4, Week 8, Week 12, Week 16, Week 24, Week 32, Week 40, Week 48, Week 60, Week 72, Week 84, Week 96, Early Term
Total Protein		
Albumin		
CK		
Total Bilirubin		
Direct Bilirubin		
Indirect Bilirubin		
AST		
ALT		
Alkaline Phosphatase		
LDH		
Amylase		
Lipase		
Electrolytes (Sodium, Potassium, Chloride, Bicarbonate)		
Calcium		
Phosphorous		
Creatinine + eGFR		
Uric Acid		
BUN		

Approved v3.0 930087119 3.0

LIPIDS		
Cholesterol	Screening	Day 1, Week 4, Week 12, Week 24
HDL		
LDL Calculated		
Triglycerides		
OTHER SERUM TESTS		
FSH	Screening	Optional; upon request
HCG	Optional; upon request	Optional; upon request
URINE		
Urine Pregnancy test (if positive, request serum HCG)	Screening	Every 4 weeks (either done at in-clinic visits, or at home)
Urine Toxicology (drugs of abuse)	Screening	
Macroscopic Urinalysis (no microscopic)	Screening	Day 1, Week 4, Week 8, Week 12, Week 16, Week 24, Week 32, Week 40, Week 48, Week 60, Week 72, Week 84, Week 96, Early Term
SEROLOGY		
HIV-1 RNA	Screening	Day 1, Week 4, Week 8, Week 12, Week 16, Week 24, Week 32, Week 40, Week 48, Week 60, Week 72, Week 84, Week 96, Early Term
HbSAg	Screening	Week 48, Week 96, Early Term
HCV Ab (if positive, reflex to HCV RNA)	Screening	Week 48, Week 96, Early Term
RESISTANCE		
Specimens for HIV Resistance testing at Monogram	Screening	Day 1, Week 4, Week 8, Week 12, Week 16, Week 24, Week 32, Week 40, Week 48, Week 60, Week 72, Week 84, Week 96, Early Term
Specimens for HIV Exploratory Resistance testing at BMS	Screening	Day 1, Week 4, Week 8, Week 12, Week 16, Week 24, Week 32, Week 40, Week 48, Week 60, Week 72, Week 84, Week 96, Early Term

Approved v3.0 930087119 3.0

PHARMACOKINETICS		
Intensive PK		Week 2 (optional)
Sparse PK		Week 4, Week 8, Week 12, Week 16, Week 24
IMMUNE PANEL		
%CD3+/CD4+	Screening	Day 1, Week 4, Week 8, Week 12, Week 16, Week 24, Week 32, Week 40, Week 48, Week 60, Week 72, Week 84, Week 96, Early Term
%CD3+/CD8+		
Absolute CD3/CD4		
Absolute CD3/CD8		
OTHER		
Pharmacodiagnostic (PDx)	Screening	
Plasma & Serum Back-ups	Screening	Day 1, Week 4, Week 8, Week 12, Week 16, Week 24, Week 32, Week 40, Week 48, Week 60, Week 72, Week 84, Week 96, Early Term

Approved v 3.0 930087119 3.0
