# Supplementary material for: Safety, efficacy, and dose response of the maturation inhibitor GSK3532795 (formerly known as BMS-955176) plus tenofovir/emtricitabine once daily in treatment-naive HIV-1-infected adults: Week 24 primary analysis from a randomized Phase IIb trial
Source: PLoS One. 2018 Oct 23;13(10):e0205368. doi: 10.1371/journal.pone.0205368 (PMC6198970; doi:10.1371/journal.pone.0205368)
Supplement: S1 Table — (A) Onset and duration of diarrhoea. EFV, efavirenz; FTC, emtricitabine; Max, maximum; Min, minimum; Q1, the first quartile; Q3, the third quartile; SD, standard deviation; TDF, tenofovir disoproxil fumarate. (DOCX) [file pone.0205368.s003.docx]

**S1 Table. (A) Onset and duration of diarrhoea.**

|  | **GSK3532795 + TDF/FTC** | **EFV  600 mg + TDF/FTC**  **(N=53)** | **Total N=206** |
| --- | --- | --- | --- |

|  | **60 mg**  **(N=50)** | **120 mg**  **(N=52)** | **180 mg**  **(N=51)** |  |  |
| --- | --- | --- | --- | --- | --- |

| Total participants with an event (n, %) | 19 (38.0) | 19 (36.5) | 31 (60.8) | 6 (11.3) | 75 (36.4) |
| --- | --- | --- | --- | --- | --- |
| **Time to onset of first event (Days)** | | | | | |
| Mean | 48.4 | 19.9 | 11.3 | 73.0 | 27.8 |
| Median | 7.0 | 2.0 | 2.0 | 24.5 | 2.0 |
| Min, Max | 1, 220 | 1, 215 | 1, 89 | 1, 238 | 1, 238 |
| Q1, Q3 | 2.0, 84.0 | 1.0, 7.0 | 1.0, 8.0 | 2.0, 148.0 | 1.0, 25.0 |
| SD | 71.0 | 49.8 | 22.4 | 98.5 | 55.6 |
| **Categorisation (%)** | | | | | |
| Day 1–7 | 10 (52.6) | 15 (78.9) | 23 (74.2) | 3 (50.0) | 51 (68.0) |
| Day 8–14 | 1 (5.3) | 0 | 2 (6.5) | 0 | 3 (4.0) |
| Day 14–21 | 1 (5.3) | 0 | 1 (3.2) | 0 | 2 (2.7) |
| Day 21–28 | 1 (5.3) | 0 | 2 (6.5) | 0 | 3 (4.0) |
| >Day 28 | 6 (31.6) | 4 (21.1) | 3 (9.7) | 3 (50.0) | 16 (21.3) |
| **Duration of first event (Days)** | | | | | |
| Mean | 82.5 | 104.2 | 85.1 | 35.8 | 85.3 |
| Median | 33.0 | 62.0 | 45.0 | 9.5 | 49.0 |
| Min, Max | 3.288 | 1, 276 | 1, 290 | 3, 125 | 1, 290 |
| Q1, Q3 | 6.0, 178.0 | 29.0, 197.0 | 18.0, 157.0 | 4.0, 64.0 | 14.0, 163.0 |
| SD | 93.2 | 98.8 | 94.7 | 49.5 | 92.6 |
| **Categorisation (%)** | | | | | |
| Day 1–3 | 1 (5.3) | 4 (21.1) | 5 (16.1) | 1 (16.7) | 11 (14.7) |
| Day 4–7 | 4 (21.1) | 0 | 1 (3.2) | 2 (33.3) | 7 (9.3) |
| Day 8–14 | 1 (5.3) | 0 | 1 (3.2) | 1 (16.7) | 3 (4.0) |
| Day 15–21 | 1 (5.3) | 0 | 1 (3.2) | 0 | 2 (2.7) |
| Day 22–28 | 1 (5.3) | 0 | 4 (12.9) | 0 | 5 (6.7) |
| >28 Days | 11 (57.9) | 15 (78.9) | 19 (61.3) | 2 (33.3) | 47 (62.7) |

EFV, efavirenz; FTC, emtricitabine; Max, maximum; Min, minimum; Q1, the first quartile;
Q3, the third quartile; SD, standard deviation; TDF, tenofovir disoproxil fumarate.
