# Supplementary material for: Safety, efficacy, and dose response of the maturation inhibitor GSK3532795 (formerly known as BMS-955176) plus tenofovir/emtricitabine once daily in treatment-naive HIV-1-infected adults: Week 24 primary analysis from a randomized Phase IIb trial
Source: PLoS One. 2018 Oct 23;13(10):e0205368. doi: 10.1371/journal.pone.0205368 (PMC6198970; doi:10.1371/journal.pone.0205368)
Supplement: S2 Table — (A) Treatment exposure. AUC, area under curve; CV, coefficient of variation; FTC, emtricitabine; TDF, tenofovir disoproxil fumarate. (DOCX) [file pone.0205368.s004.docx]

**S2 Table. (A) Summary of BMS-955176 PK Parameters (non-compartmental methods).**

| **Parameter, geometric mean [N] (% CV)** | **GSK3532795 +TDF/FTC** |
| --- | --- |

|  | **60 mg** | **120 mg** | **180 mg** |
| --- | --- | --- | --- |
| Cmax (ng/mL) | 1945.342 [8] (16.0) | 3162.161 [6] (28.8) | 4645.266 [10] (16.2) |
| Tmax (h) | 3.669 [8]  (49.00) | 4.422 [6]  (11.85) | 4.881 [10]  (51.02) |
| Ctau (ng/mL) | 1100.138 [9] (15.1) | 1656.578 [6] (39.7) | 2705.751 [10] (26.8) |
| C0 (ng/mL) | 1065.102 [8] (25.2) | 1800.952 [6] (33.4) | 2728.671 [10] (17.8) |
| AUC(TAU) (ng·h/mL) | 34226.751 [8] (18.73) | 55251.956 [6] (32.88) | 87128.359 [10] (20.79) |

AUC, area under curve; CV, coefficient of variation; FTC, emtricitabine;
TDF, tenofovir disoproxil fumarate.
