# Supplementary material for: Safety, efficacy, and dose response of the maturation inhibitor GSK3532795 (formerly known as BMS-955176) plus tenofovir/emtricitabine once daily in treatment-naive HIV-1-infected adults: Week 24 primary analysis from a randomized Phase IIb trial
Source: PLoS One. 2018 Oct 23;13(10):e0205368. doi: 10.1371/journal.pone.0205368 (PMC6198970; doi:10.1371/journal.pone.0205368)
Supplement: S3 Table — (A) Dose proportionality assessment for GSK3532795 Cmax and AUC(TAU) in the evaluable population. AUC, area under curve calculated by linear trapezoidal rule from time zero to the end of the dosing interval at steady state; CI, confidence interval; Cmax, maximum serum concentration. (DOCX) [file pone.0205368.s005.docx]

**S3 Table. (A) Dose proportionality** **assessment for GSK3532795 Cmax and AUC(TAU) in the evaluable population.**

| **Parameter** | **Estimated slope** | **90% CI of the slope** |
| --- | --- | --- |

| Cmax (ng/mL) | 0.788 | (0.621, 0.995) |
| --- | --- | --- |
| AUC(_tau_) **(**ng·h/mL) | 0.843 | (0.648, 1.037) |

AUC, area under curve calculated by linear trapezoidal rule from time zero to the end of the dosing interval at steady state; CI, confidence interval; C_max_, maximum serum concentration.
