# Supplementary material for: Safety, efficacy, and dose response of the maturation inhibitor GSK3532795 (formerly known as BMS-955176) plus tenofovir/emtricitabine once daily in treatment-naive HIV-1-infected adults: Week 24 primary analysis from a randomized Phase IIb trial
Source: PLoS One. 2018 Oct 23;13(10):e0205368. doi: 10.1371/journal.pone.0205368 (PMC6198970; doi:10.1371/journal.pone.0205368)
Supplement: S4 Table — (A) Parameter estimates from the final GSK3532795 population PK model. CIs are derived from the bootstrap. CI, confidence interval; CL, clearance; CV, coefficient of variation; RSE, residual standard error; PK, pharmacokinetic; SD, standard deviation. (DOCX) [file pone.0205368.s006.docx]

**S4 Table. (A) Parameter estimates from the final GSK3532795 population PK model. CIs are derived from the bootstrap.**

| **Description** | **Model** | **Estimate** | **RSE** | **95% CI** | **Variability** |
| --- | --- | --- | --- | --- | --- |

| Clearance | CL ~ *θ*_1_ - e^ƞ1^ | 2.24 *L/h* | 3.79 | (2.08, 2.45) |  |
| --- | --- | --- | --- | --- | --- |
| Volume of distribution | V ~ *θ*_2_ - e^ƞ2^ | 5.58 *L* | 16.7 | (4.05, 9.21) |  |
| First-order absorption rate | Ka ~ *θ*_3_ - e ^ƞ3^ | 0.0236 *1/h* | 12.2 | (0.0172, 0.0318) |  |
| Black or African-American race effect on CL | AR_CL_ ~ *θ*_4_ | 0.768 | 7.34 | (0.661, 0.891) |  |
| Other race effects on CL | OR_CL_ ~ *θ*_5_ | 0.983 | 7.60 | (0.857, 1.15) |  |
| Grade 1 diarrhoea effect on CL | Dia1_CL_ ~ *θ*_6_ | 1.00 | 4.24 | (0.921, 1.09) |  |
| Grade 2 or greater diarrhoea effect on CL | Dia2_CL_ ~ *θ*_7_ | 1.18 | 8.11 | (1.01, 1.40) |  |
| Interindividual variance of CL | IIV_CL_ ~ Ω_1,1_ | 0.0610 | 16.9 | (0.0390, 0.0816) | %CV = 25.1 |
| Interindividual variance of V | IIV_V_ ~ Ω_2,2_ | 0.462 | 43.3 | (6.46e-10, 0.850) | %CV = 76.6 |
| Interindividual variance of Ka | IIV_Ka_ ~ Ω_3,3_ | 0.164 | 43.5 | (1.00e-06, 0.452) | %CV = 42.2 |
| Interoccasion variance of F1 | IOV_F1_ ~ Ω_4,4_ | 0.0512 | 18.0 | (1.77e-06, 0.0728) | %CV = 22.9 |
| Proportional residual error | err_prop_ ~ ∑_1,1_ | 0.0165 | 25.7 | (0.00819, 0.0463) | %CV = 12.9 |
| Additive residual error | err_add_ ~ ∑_2,2_ | 37400 | 21.7 | (20100, 58900) | SD = 193 |

CI, confidence interval; CL, clearance; CV, coefficient of variation; RSE, residual standard error; PK, pharmacokinetic; SD, standard deviation.
