# Supplementary material for: Safety, efficacy, and dose response of the maturation inhibitor GSK3532795 (formerly known as BMS-955176) plus tenofovir/emtricitabine once daily in treatment-naive HIV-1-infected adults: Week 24 primary analysis from a randomized Phase IIb trial
Source: PLoS One. 2018 Oct 23;13(10):e0205368. doi: 10.1371/journal.pone.0205368 (PMC6198970; doi:10.1371/journal.pone.0205368)
Supplement: S1 Fig — (A) Fraction of participants with viral load <40 copies/mL at Week 24 versus steady state AUC: mITT population. (B) Exposure-response relationship for Grade 1 GI AEs. (C) Exposure-response relationship for Grade 2 GI AEs. Mean response is plotted at the mean of the exposure quantile. The observed values are also plotted against individual exposures, represented by black filled circles. The dashed blue line represents a loess smooth of the individual data, with a shaded blue region representing the 95% CI of the loess smooth. Range of exposures within a quartile group is shown at the bottom of the figure as a horizontal line with vertical ticks representing minimum and maximum values. The values along the horizontal line indicate the number of individuals within each exposure quartile/covariate group. The horizontal bars at the top of the figure show the overall PK variability in exposure following 60 mg, 120 mg, or 180mg GSK3532795. (DOCX) [file pone.0205368.s007.docx]

**S1 Fig.** **(A) Fraction of participants with viral load <40 copies/mL at Week 24 versus steady state AUC: mITT population. (B) Exposure-response relationship for Grade 1 GI AEs. (C) Exposure-response relationship for Grade 2 GI AEs.**

A)


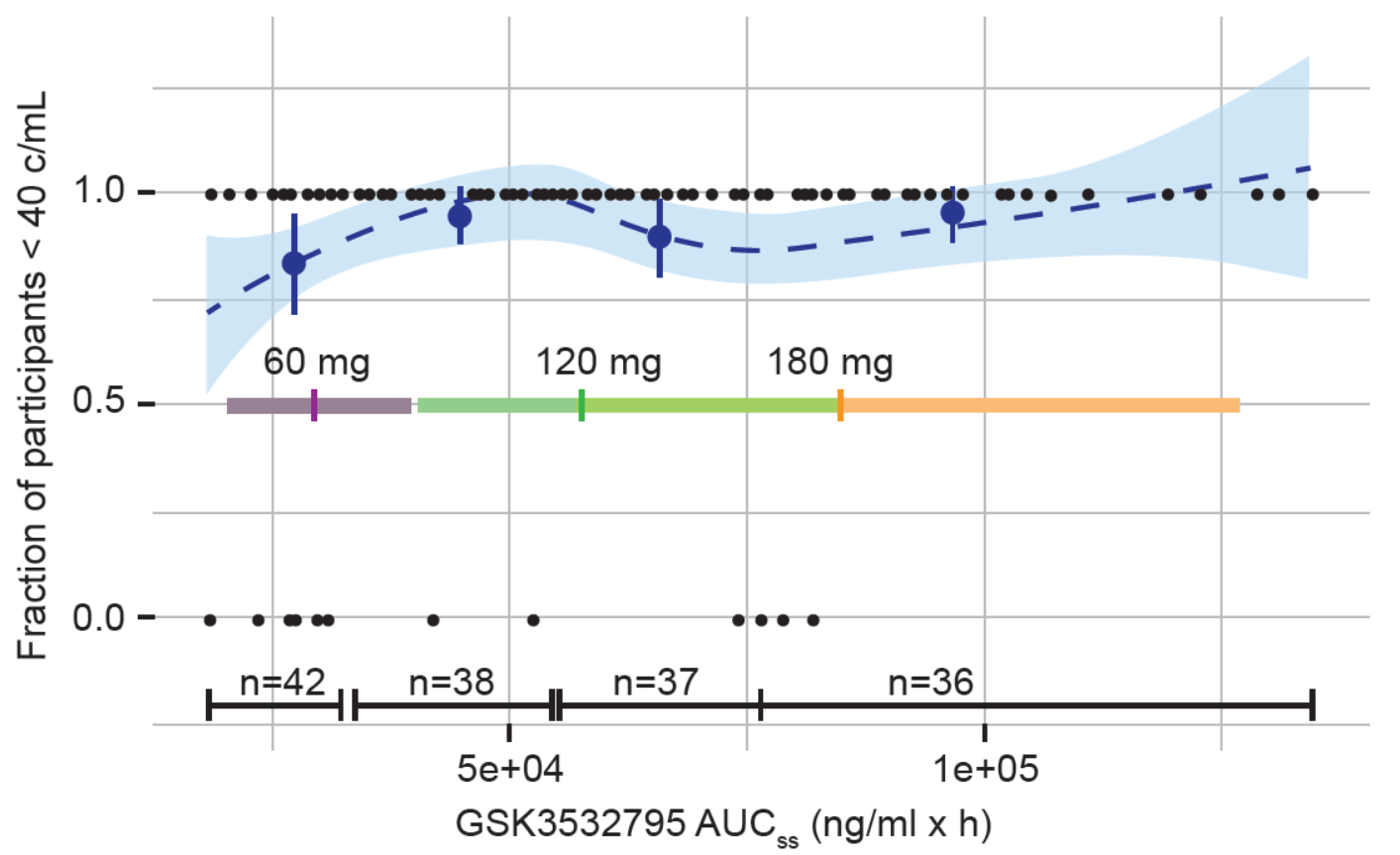


B)


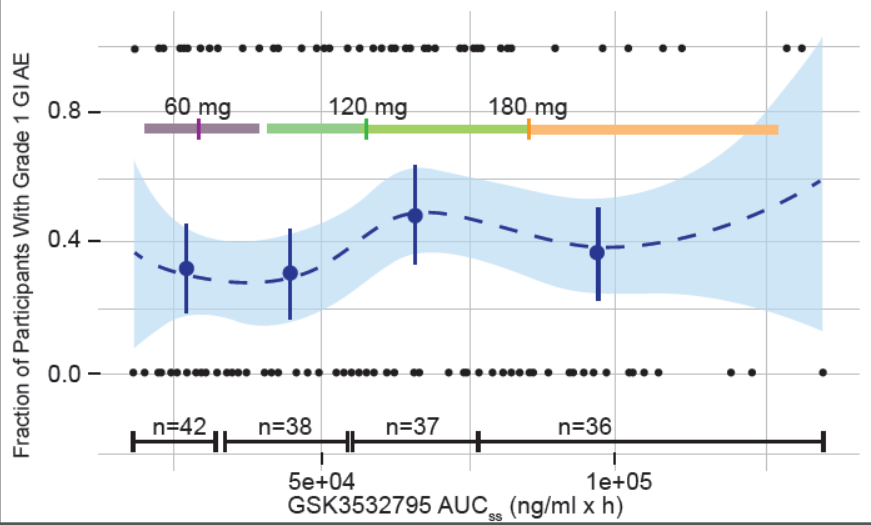


C)


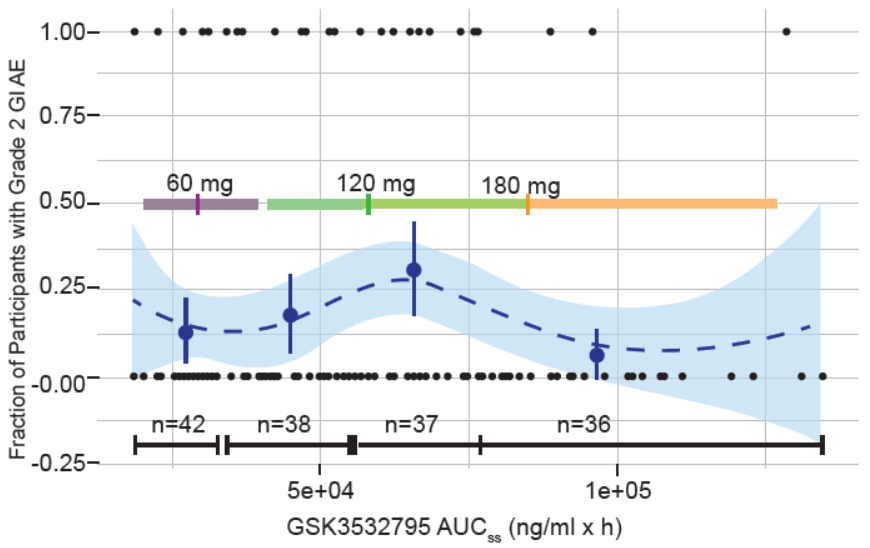


Mean response is plotted at the mean of the exposure quantile. The observed values are also plotted against individual exposures, represented by black filled circles. The dashed blue line represents a loess smooth of the individual data, with a shaded blue region representing the 95% CI of the loess smooth. Range of exposures within a quartile group is shown at the bottom of the figure as a horizontal line with vertical ticks representing minimum and maximum values. The values along the horizontal line indicate the number of individuals within each exposure quartile/covariate group. The horizontal bars at the top of the figure show the overall PK variability in exposure following 60 mg, 120 mg, or 180mg GSK3532795.
