# Supplementary material for: Safety, efficacy, and dose response of the maturation inhibitor GSK3532795 (formerly known as BMS-955176) plus tenofovir/emtricitabine once daily in treatment-naive HIV-1-infected adults: Week 24 primary analysis from a randomized Phase IIb trial
Source: PLoS One. 2018 Oct 23;13(10):e0205368. doi: 10.1371/journal.pone.0205368 (PMC6198970; doi:10.1371/journal.pone.0205368)
Supplement: S1 Text — (DOCX) [file pone.0205368.s008.docx]

**Supporting information**

**Additional methods**

Population pharmacokinetic (PK) analyses were conducted via nonlinear mixed-effects modelling using nonlinear mixed effects modeling (NONMEM) software, version 7, level 2.0 (ICON Development Solutions, Hanover, MD). Assessment of model adequacy and decision about increasing model complexity was driven by the data and guided by goodness-of-fit criteria, including visual inspection of diagnostic scatter plots (observed versus predicted concentration, residual/weighted residual versus predicted concentration or time and histograms of individual random effects, for example), successful convergence of the minimization routine, plausibility of parameter estimates, precision of parameter estimates, correlation between model parameter estimation errors < 0.95, and the Akaike information criterion (AIC), given the minimum objective function value and number of estimated parameters[1,2]. An exploratory investigation of covariate-parameter relationships was undertaken as part of the population PK analysis. Predictive performance was assessed using stratified non-parametric bootstrapping (n=500). Following the construction of a suitable PK model, individual-specific steady-state exposure metrics were derived for exposure-response investigation. An exploratory plotting analysis was performed to investigate exposure–response relationships for efficacy and safety endpoints, where endpoints including fraction of participants with <40 c/mL at Week 24 (modified intent-to-treat population) and percentage of participants with Grade 1, 2, 3, and 4 gastrointestinal events (adverse events >7 days) were analysed as a function of exposure quantiles.
